# Influence of Methoxy Substitution Pattern on Cascade Biotransformation of 4′-Hydroxychalcones by Entomopathogenic Fungi

**DOI:** 10.3390/ijms27167463

**Published:** 2026-08-20

**Authors:** Paweł Chlipała, Julia Bienia, Tomasz Tronina, Jerzy Ł. Wiśniewski, Tomasz Janeczko

**Affiliations:** 1 Department of Food Chemistry and Biocatalysis, Faculty of Biotechnology and Food Science, Wrocław University of Environmental and Life Sciences, 50-375 Wrocław, Poland; 125375@student.upwr.edu.pl (J.B.); tomasz.tronina@upwr.edu.pl (T.T.); 2 Department of Biochemistry, Molecular Biology and Biotechnology, Faculty of Chemistry, Wrocław University of Science and Technology, Łukasiewicza 2, 50-371 Wrocław, Poland; jerzy.wisniewski@pwr.edu.pl

**Keywords:** entomopathogenic fungi, chalcones, hydroxychalcones, biotransformation, cascade metabolism, dihydrochalcones, methylglucosylation, *O*-demethylation

## Abstract

4′-Hydroxymethoxychalcones represent structurally diverse chalcone derivatives that are attractive substrates for microbial functionalization; however, the impact of methoxy substitution position on their cascade biotransformation by fungi remains poorly understood. In this study, three regioisomeric 4′-hydroxymethoxychalcones, featuring *ortho*-, *meta*-, or *para*-methoxy groups on ring B, were transformed using eight entomopathogenic fungal strains belonging to the genera *Beauveria*, *Isaria*, and *Metarhizium*. Metabolic profiles were monitored over a 10-day period using ultra-high-performance liquid chromatography coupled with diode-array detection (UHPLC-DAD), and the structures of the major products were elucidated primarily by one- and two-dimensional nuclear magnetic resonance (NMR) spectroscopy and further supported by high-resolution electrospray ionization quadrupole time-of-flight mass spectrometry (HR-ESI-QTOF-MS). The investigated microorganisms catalyzed multistep transformations encompassing the reduction of the α,β-unsaturated carbonyl system, methylglucosylation, *O*-demethylation, and the formation of secondary polar metabolites. Although ene-reduction constituted the predominant initial reaction for all substrates, the relative distribution of subsequent metabolites varied depending on both the fungal strain and, to a lesser extent, the position of the methoxy group. The ortho-methoxy derivative exhibited the highest propensity for *O*-demethylation; the meta-substituted substrate generated the most heterogeneous secondary metabolite profiles, whereas the para-methoxy analogue showed the most consistent accumulation of methylglucosylated dihydrochalcones. These findings indicate that methoxy substitution position does not alter the common core biotransformation pathway, but can modulate the relative efficiency of individual steps and the extent of secondary metabolism.

## 1. Introduction

Chalcones (1,3-diaryl-2-propen-1-ones) constitute an important class of naturally occurring and synthetic α,β-unsaturated ketones [1,2,3]. Hydroxy-substituted chalcones are widely represented among natural polyphenolic compounds. In particular, 2′-hydroxychalcones are important intermediates in flavonoid biosynthesis, as the 2′-hydroxy group enables intramolecular cyclization to the corresponding flavanone scaffold [4,5]. Their characteristic structural feature, consisting of two aromatic rings linked by an α,β-unsaturated carbonyl system, provides a highly reactive conjugated scaffold susceptible to nucleophilic addition and enzymatic transformation [6,7,8]. Due to the presence of this electrophilic enone moiety, chalcones readily undergo interactions with biological nucleophiles and serve as attractive substrates for both chemical and biocatalytic modifications [9,10,11]. Chalcone derivatives exhibit a broad spectrum of biological activities, including anti-inflammatory, antioxidant, antimicrobial, antimalarial, and anticancer effects [12,13,14,15,16]. Their structurally accessible α,β-unsaturated carbonyl system and substituted aromatic rings also make them attractive substrates for chemical and biocatalytic functionalization, including ene-reduction, hydroxylation, *O*-demethylation, glycosylation, and cyclization, thereby providing access to structurally diverse dihydrochalcones, flavanones, and conjugated derivatives [9,17,18,19].

Hydroxy-substituted chalcones bearing a 4′-hydroxy group are well represented among natural products [20]. Particularly relevant examples occur in the red resins collectively known as ‘dragon’s blood’, obtained from several *Dracaena* species, where structurally diverse chalcones and dihydrochalcones constitute characteristic flavonoid components [21,22]. Among these compounds, hydroxy-methoxylated analogues represent particularly attractive substrates for microbial metabolism studies [23]. The presence and position of methoxy substituents substantially influence electronic distribution, steric accessibility, and reactivity of the α,β-unsaturated carbonyl system, thereby affecting susceptibility toward enzymatic reduction, oxidative transformations, and glycosylation reactions [24,25,26]. In particular, regioisomeric methoxy substitution may critically determine the balance between competing metabolic pathways and influence stability of intermediate metabolites formed during fungal biotransformation [27,28]. The biological relevance of methoxy topology in 4′-hydroxychalcones was also recently demonstrated in integrated cytotoxicity, erythrocyte compatibility, hemoglobin-binding, antioxidant, and in silico profiling, where the position and number of methoxy substituents shaped both potency and interactions with blood components [29].

Microbial biotransformation has emerged as an efficient and sustainable approach for selective structural modification of natural products. Compared with conventional synthetic methodologies, filamentous fungi frequently enable highly regioselective and chemoselective transformations under mild reaction conditions. Entomopathogenic fungi, including representatives of the genera *Beauveria*, *Isaria*, and *Metarhizium*, possess versatile enzymatic systems capable of catalyzing multistep cascade reactions involving oxidoreductases, transferases, and hydrolytic enzymes [19,30,31,32]. These microorganisms are additionally recognized for their ability to mimic mammalian phase I and phase II metabolism, making them valuable biocatalytic models for generation of structurally diverse metabolites [33].

A particularly important role in metabolism of α,β-unsaturated carbonyl compounds is played by enzymes belonging to the Old Yellow Enzyme (OYE) family. These flavin-dependent ene-reductases catalyze stereoselective NAD(P)H-dependent reduction of activated C=C bonds, leading to formation of saturated derivatives such as dihydrochalcones [34]. Reduction of the α,β-unsaturated system substantially alters molecular geometry and electronic distribution, which may subsequently influence downstream enzymatic transformations including glycosylation, hydroxylation, and *O*-demethylation reactions. Consequently, fungal metabolism of chalcones frequently proceeds through complex cascade pathways rather than simple single-step conversions [19,24,35].

Glycosylation significantly alters the physicochemical properties of flavonoids by increasing their hydrophilicity and aqueous solubility, while generally decreasing lipophilicity and membrane permeability. Moreover, attachment of a sugar moiety may affect chemical stability, protein binding, bioavailability, and biological activity [25,36,37]. In fungal metabolism, glycosylation frequently functions as a detoxification mechanism [27]; however, it may also generate metabolites with altered biological activity and improved pharmacokinetic properties [38,39,40]. Particularly interesting are methylglucosylated derivatives produced by entomopathogenic fungi, as these relatively uncommon conjugates are difficult to obtain using conventional synthetic approaches and may exhibit distinct biological and physicochemical characteristics compared with their parent aglycones [41,42,43].

Although fungal biotransformations of chalcones have been investigated previously, most studies have focused primarily on isolation and structural characterization of final metabolites [30,44,45]. In contrast, considerably less attention has been devoted to the progression of multistep metabolic pathways, the formation of intermediate products, and competition between parallel enzymatic pathways [19,44]. Furthermore, the influence of methoxy substitution pattern on pathway selectivity and metabolite distribution in fungal chalcone metabolism remains poorly understood.

Our previous studies demonstrated that non-conventional yeast strains can efficiently reduce 4′-hydroxychalcones to the corresponding 4′-hydroxydihydrochalcones, confirming the high susceptibility of this scaffold to whole-cell ene-reduction [46,47]. However, yeast-mediated transformations were mainly directed toward the formation of reduced products, whereas entomopathogenic filamentous fungi are known to possess broader metabolic potential, including glycosylation and other phase-I- and phase-II-like modifications of flavonoid compounds.

We hypothesized that the position of the methoxy substituent within ring B would modulate the direction of whole-cell cascade metabolism by affecting the balance between ene-reduction, glycosylation, *O*-demethylation, and secondary oxidative or conjugative transformations. To verify this hypothesis, three regioisomeric 4′-hydroxymethoxychalcones bearing methoxy groups at the *ortho*-, *meta*-, and *para*-positions of ring B were selected as model substrates. Their biotransformations were investigated using selected entomopathogenic fungal strains: *Beauveria bassiana* KCh BBT, *B*. *bassiana* KCh J1, *B. bassiana* KCh J1.5, *Beauveria caledonica* KCh J3.3, *Isaria fumosorosea* KCh J2, *Isaria tenuipes* MU35, *Isaria farinosa* KCh KW1.1, and *Metarhizium robertsii* MU4. The study focused on mapping dominant metabolic routes and identifying major products formed through ene-reduction, methylglucosylation, *O*-demethylation, and secondary metabolism. This approach allowed comparison of the metabolic plasticity of different entomopathogenic strains and clarified how the methoxy substitution pattern influences the direction of cascade biotransformation.

## 2. Results and Discussion

### 2.1. General Metabolic Trends

**Scheme 1:**
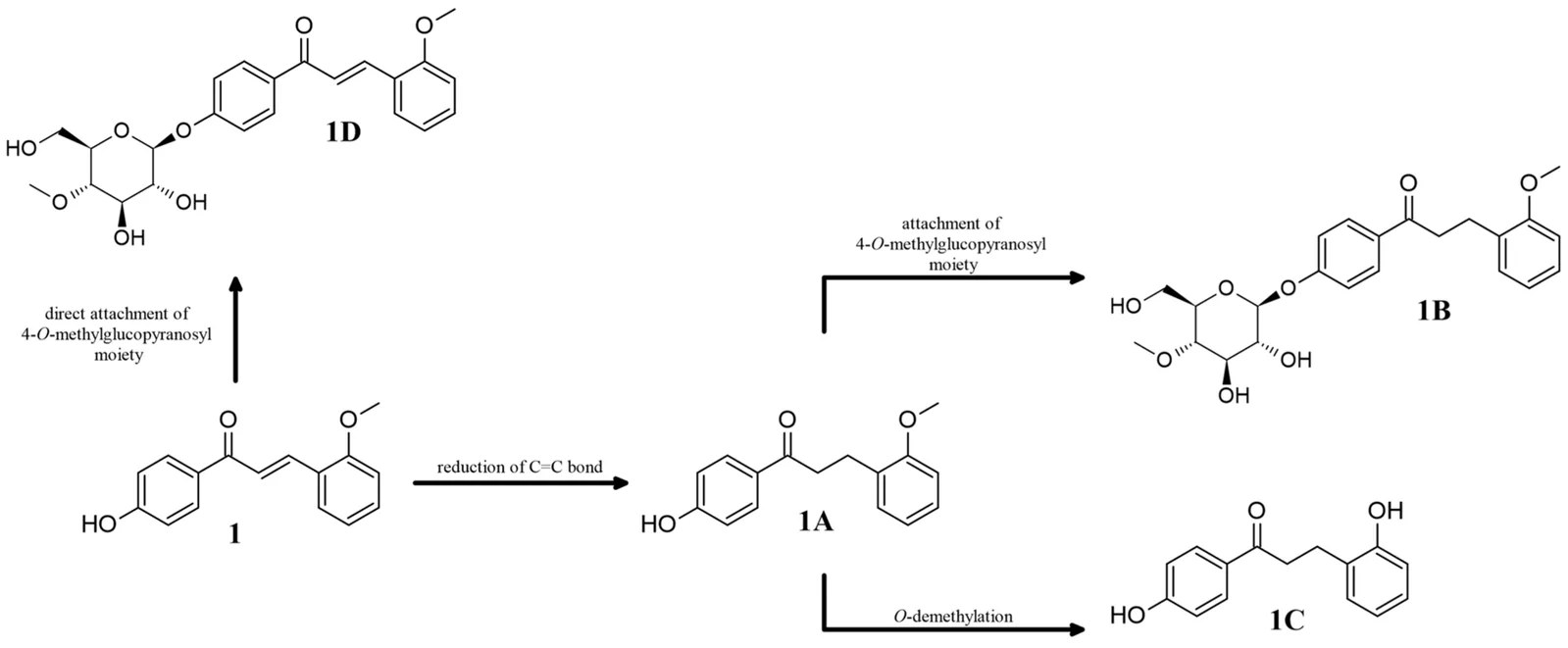
Proposed cascade biotransformation pathways of (**1**) in entomopathogenic filamentous fungi.

**Scheme 2:**
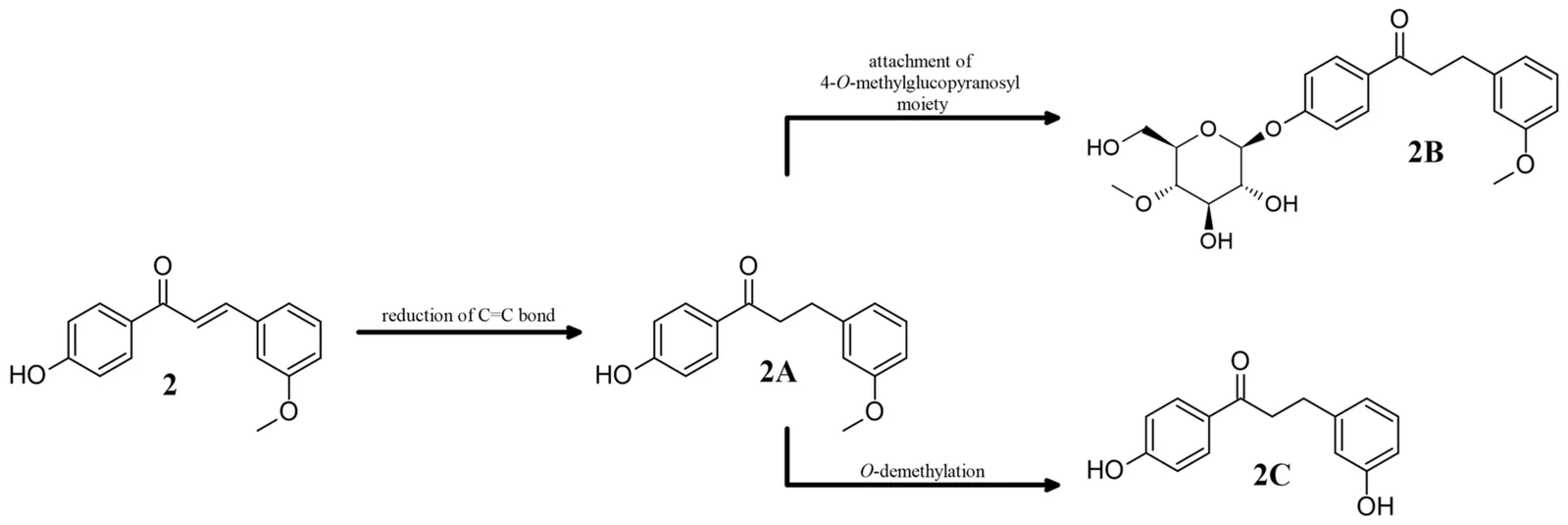
Proposed cascade biotransformation pathways of (**2**) in entomopathogenic filamentous fungi.

**Scheme 3:**
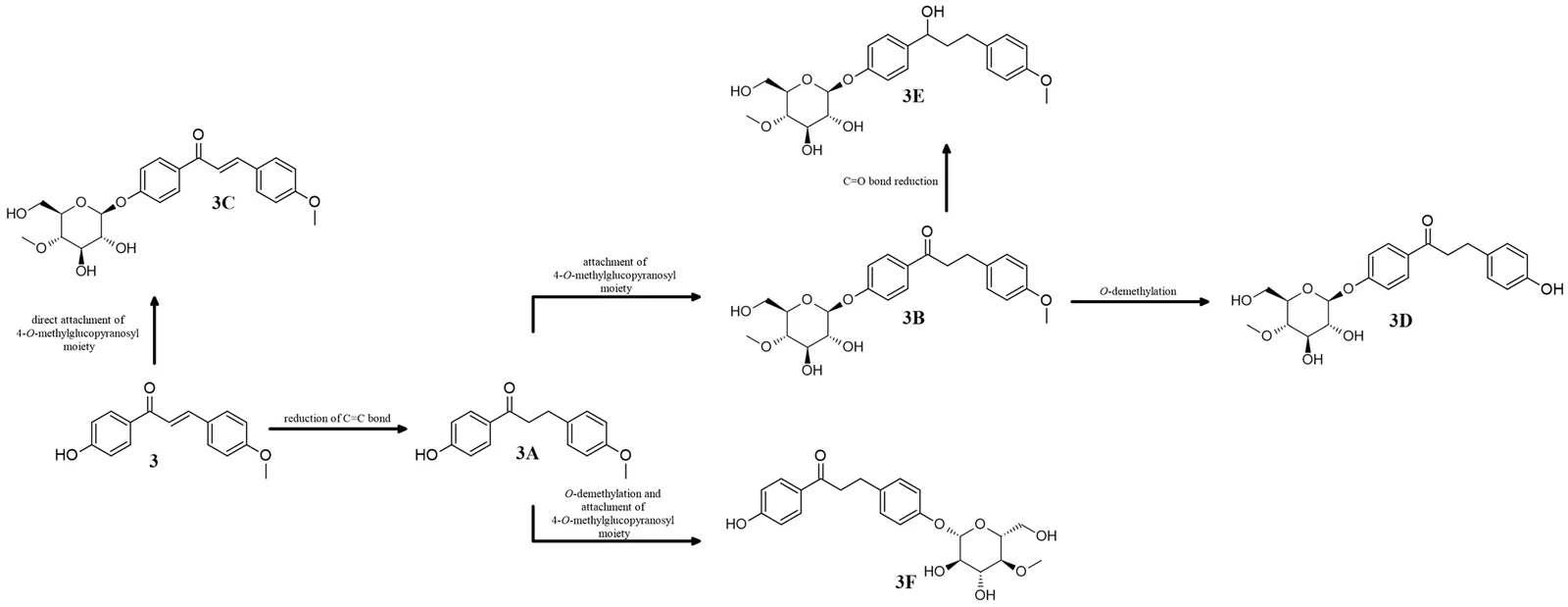
Suggested cascade biotransformation pathways of (**3**) in entomopathogenic filamentous fungi.

**Figure 1:**
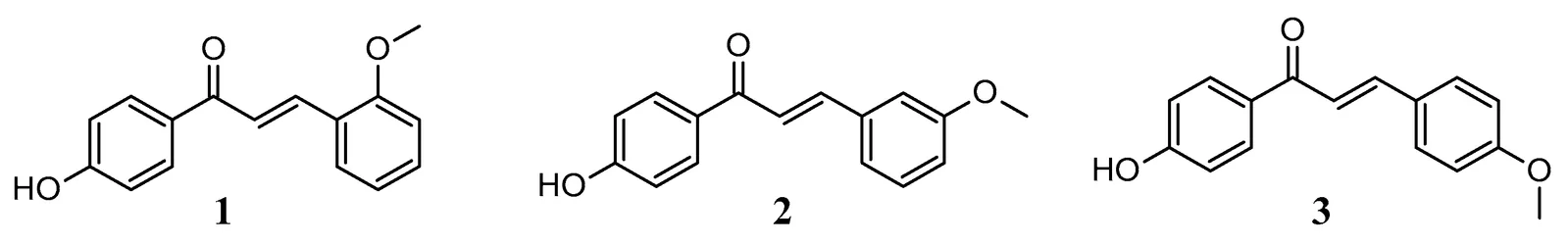
Chemical structures of the investigated 4′-hydroxymethoxychalcones: 4′-hydroxy-2-methoxychalcone (**1**), 4′-hydroxy-3-methoxychalcone (**2**), and 4′-hydroxy-4-methoxychalcone (**3**).

The biotransformation of three regioisomeric 4′-hydroxymethoxychalcones (**1**–**3**; Figure 1) was investigated using eight entomopathogenic fungal strains over a 10-day cultivation period. Metabolic profiles were monitored by ultra-high-performance liquid chromatography coupled with diode-array detection (UHPLC-DAD) analysis of culture extracts collected after 1, 3, 7, and 10 days. In accordance with the exploratory design of the screening experiment, the chromatographic data were interpreted qualitatively and semi-quantitatively, with emphasis on the disappearance of the parent substrate signal, appearance of characteristic metabolite classes, and selection of representative strain–substrate systems for preparative identification. Compounds isolated on the preparative scale were identified by nuclear magnetic resonance (NMR) spectroscopy and subsequently used as chromatographic references. Signals matching these products in both retention time and UV-DAD spectral profile were assigned accordingly, whereas additional signals were described tentatively according to their chromatographic and spectral characteristics. Relative peak–area comparisons were restricted to closely related metabolites displaying comparable DAD spectral profiles and are reported only as uncorrected relative detector–response ratios; they do not represent absolute concentrations, molar product distributions, conversion values, or biotransformation yields. To facilitate direct comparison of the strain- and time-dependent metabolic profiles, the complete UHPLC-DAD screening results for all three substrates, eight fungal strains, and four sampling points (1, 3, 7, and 10 days) are summarized in Table S2. The detected substrates and biotransformation products are denoted using the compound numbering applied in Scheme 1, Scheme 2 and Scheme 3, and the predominant components of each chromatographic profile are indicated. Representative time-course UHPLC-DAD chromatograms and wavelength-resolved DAD maps are provided in Figures S40–S45. The investigated strains generated diverse metabolic profiles depending on both fungal strain and methoxy substitution pattern, with the main observed transformations including reduction of the α,β-unsaturated system, methylglucosylation, *O*-demethylation, and formation of secondary polar metabolites.

The present study demonstrates that entomopathogenic filamentous fungi are versatile biocatalysts for the cascade transformation of hydroxy-methoxylated chalcones. The investigated substrates differed only in the position of the methoxy group on ring B. Although the major biotransformation sequence was broadly conserved across all three regioisomers, methoxy position moderately affected the relative distribution of products and the extent of secondary transformations. Similar strain- and substrate-dependent behavior has previously been observed during the fungal transformation of methylflavonoids, halogenated flavonoids, and prenylated chalcones, where relatively small structural changes determined whether reduction, glycosylation, hydroxylation, oxidation, or cyclization became the dominant pathway [19,30].

Reduction of the α,β-unsaturated carbonyl system was one of the earliest and most common transformations observed for the investigated hydroxychalcones. This is consistent with previous reports showing that chalcone derivatives are readily converted into the corresponding dihydrochalcones by microbial whole-cell systems. Previous research indicated that 2′-hydroxychalcones bearing bromine atoms at different positions were converted into their corresponding dihydrochalcones, while other microbial systems were also able to hydrogenate activated C=C bonds with high apparent transformation capacity [24]. Therefore, the reduction of substrates **1**–**3** observed in the present study should be interpreted as a typical primary metabolic response of fungal cells toward the electrophilic chalcone scaffold.

Formation of dihydrochalcone derivatives was supported by NMR analysis of isolated or enriched metabolites, which showed disappearance of the characteristic trans-olefinic proton signals and appearance of aliphatic methylene signals corresponding to the dihydrochalcone scaffold. In several strain–substrate systems, reduced intermediates were followed by accumulation of methylglucosylated dihydrochalcones as dominant late-stage metabolite classes, which is consistent with previous reports on fungal glycosylation of flavonoid scaffolds [19,30,35]. The sequence and relative abundance of reduction, glycosylation, and *O*-demethylation products differed substantially between fungal strains and substrate regioisomers.

The position of the methoxy substituent moderately influenced the relative distribution of metabolic products and the extent of secondary transformations. The *ortho*-methoxy derivative displayed the highest tendency toward secondary *O*-demethylation, whereas the *para*-substituted analogue showed the most consistent reduction–glycosylation sequence. The *meta*-methoxy substrate generated the most heterogeneous secondary metabolite profiles. These differences suggest that methoxy position may modulate substrate recognition and/or susceptibility of the α,β-unsaturated system toward fungal oxidoreductases, while its direct influence on subsequent glycosylation appears less pronounced.

Several fungal strains additionally produced highly polar secondary metabolites during prolonged cultivation. Based on retention behavior and UV spectral characteristics, these compounds were tentatively assigned as hydroxylated, demethylated, or further glycosylated derivatives of dihydrochalcones. Their formation indicates that fungal metabolism of hydroxy-methoxychalcones proceeds through complex multistep cascade pathways rather than simple single-step conversions.

Interestingly, selected strains, particularly *Beauveria bassiana* KCh J1.5 and *Isaria fumosorosea* KCh J2, generated metabolites tentatively identified as chalcone glycosides retaining the α,β-unsaturated system. This observation suggests that glycosylation may directly compete with ene-reductase activity and, in certain strains, may occur prior to reduction of the C=C bond. Such pathway divergence highlights the substantial metabolic plasticity of entomopathogenic fungi and demonstrates pronounced strain-dependent differences in enzymatic selectivity.

### 2.2. Biotransformation of the 4′-Hydroxy-2-methoxychalcone

Biotransformation of the *ortho*-methoxy-substituted chalcone (**1**) revealed substantial strain-dependent differences in the balance between reduction, glycosylation, and secondary oxidative metabolism. In most investigated fungal strains, reduction of the α,β-unsaturated system represented the dominant initial metabolic transformation, leading to formation of the corresponding dihydrochalcone which was already detected at the first 24 h of cultivation. Simultaneously, several strains exhibited pronounced glycosylation tendency, resulting in early accumulation of methylglucosylated dihydrochalcone derivatives as major metabolites. The suggested metabolic routes of (**1**) are summarized in Scheme 1.

The most pronounced methylglucosylation-oriented profile was observed for *Beauveria caledonica* KCh J3.3 and *Beauveria bassiana* KCh BBT, in which methylglucosylated dihydrochalcone (**1B**) became the dominant metabolite class after 24 h. Only minor amounts of intermediate dihydrochalcone (**1A**) and residual substrate were detected during early cultivation stages, whereas prolonged incubation resulted in marked depletion of the parent substrate signal and predominance of methylglucosylated products without formation of significant secondary metabolites. Similar metabolic behavior was observed for *Isaria tenuipes* MU35, although trace amounts of unreacted substrate (**1**) persisted slightly longer during cultivation.

The structures of the major biotransformation products were established by ^1^H and ^13^C NMR spectroscopy supported by COSY, HSQC, and HMBC experiments. Reduction of the α,β-unsaturated chalcone system was confirmed by disappearance of the characteristic trans-olefinic proton doublets and appearance of aliphatic methylene signals corresponding to the dihydrochalcone scaffold. Glycosylated products were identified by the presence of anomeric proton and carbon signals together with characteristic sugar resonances. Methylglucosylated derivatives additionally displayed methoxy resonances assigned to the 4″-*O*-methylglucopyranosyl moiety. *O*-Demethylated products were recognized by disappearance of the corresponding aromatic methoxy resonance and changes in the aromatic carbon pattern. These types of biotransformations were previously reported [19]. The ^1^H NMR data of the major products are presented below, and the corresponding ^13^C NMR assignments are summarized in Table 1.

4′-hydroxy-2-methoxychalcone (**1**): ^1^H NMR (600 MHz, DMSO-d_6_) δ (ppm): 10.42 (s, 1H, C-4′-OH), 8.02–8.06 (m, 2H, H-2′ and H-6′), 7.99 (d, 1H, J = 15.6 Hz, H-β), 7.94 (dd, 1H, J = 7.7, 1.6 Hz, H-6), 7.85 (d, 1H, J = 15.8 Hz, H-α), 7.43 (ddd, 1H, J = 8.2, 7.5, 1.6 Hz, H-4), 7.10 (d, 1H, J = 8.2 Hz, H-3), 7.02 (t, 1H, J = 7.5 Hz, H-5), 6.87–6.92 (m, 2H, H-3′ and H-5′), 3.89 (s, 3H, C-2-OCH_3_).

4′-hydroxy-2-methoxydihydrochalcone (**1A**): ^1^H NMR (600 MHz, DMSO-d_6_) δ (ppm): 10.33 (s, 1H, C-4′-OH), 7.83–7.86 (m, 2H, H-2′ and H-6′), 7.18 (td, 1H, J = 7.4, 1.7 Hz, H-4), 7.17 (dd, 1H, J = 7.4, 0.7 Hz, H-6), 6.94 (dd, 1H, J = 8.6, 0.8 Hz, H-3), 6.85 (td, 1H, J = 7.4, 1.0 Hz, H-5), 6.81–6.84 (m, 2H, H-3′ and H-5′), 3.78 (s, 3H, C-2-OCH_3_), 3.12–3.16 (m, 2H, H-α), 2.83–2.87 (m, 2H, H-β). HRESI-MS: [M + H]^+^; *m*/*z* 257.1193 (calcd. 257.1172; mass error = +8.1 ppm).

4′-O-β-D-(4″-O-methylglucopyranosyl)-2-methoxydihydrochalcone (**1B**): ^1^H NMR (600 MHz, DMSO-*d*_6_) δ (ppm): 7.92–7.96 (m, 2H, H-2′ and H-6′), 7.16–7.20 (m, 2H, H-4 and H-6), 7.08–7.12 (m, 2H, H-3′ and H-5′), 6.95 (dd, 1H, *J* = 8.6, 0.8 Hz, H-3), 6.85 (td, 1H, J = 7.4, 1.0 Hz, H-5), 5.44 (d, 1H, *J* = 5.3 Hz, C-2″-OH), 5.28 (d, 1H, *J* = 5.5 Hz, C-3″-OH), 5.03 (d, 1H, *J* = 7.8 Hz, C-1″), 4.71 (dd, 1H, *J* = 6.1, 5.3 Hz, C-6″-OH), 3.78 (s, 3H, C-2-OCH_3_), 3.63 (ddd, 1H, *J* = 11.6, 4.6, 1.6 Hz, one of C-6″), 3.50 (ddd, 1H, *J* = 11.6, 6.2, 4.9 Hz, one of C-6″), 3.46 (s, 3H, C-4″-OCH_3_), 3.41–3.45 (m, 2H, C-3″ and C-5″), 3.27 (ddd, *J* = 8.9, 8.1, 5.2 Hz, C-2″), 3.18–3.22 (m, 2H, H-α), 3.05 (t, 1H, *J* = 9.4 Hz, C-4″), 2.85–2.89 (m, 2H, H-β). HRESI-MS: [M + H]^+^; *m*/*z* 433.1870 (calcd. 433.1857; mass error = +3.0 ppm).

2,4′-dihydroxydihydrochalcone (**1C**): ^1^H NMR (600 MHz, Acetone-*d*_6_) δ (ppm): 7.93–7.95 (m, 2H, H-2′ and H-6′), 7.16 (dd, 1H, J = 7.5, 1.4 Hz, H-6), 7.03 (td, 1H, J = 7.8, 1.7 Hz, H-4), 6.90–6.93 (m, 2H, H-3′ and H-5′), 6.84 (dd, 1H, J = 8.0, 1.1 Hz, H-3), 6.76 (td, 1H, J = 7.4, 1.1 Hz, H-5), 3.24–3.28 (m, 2H, H-α), 2.95–2.99 (m, 2H, H-β).

For clarity and to facilitate direct comparison of structural changes occurring during biotransformation, the ^13^C NMR data of the parent substrates are presented together with those of the major metabolites. Although the spectra of the substrates were reported previously, their inclusion in Table 1 provides reference values for diagnostic carbon shifts associated with reduction of the α,β-unsaturated system, glycosylation, methylglucosylation, and *O*-demethylation.

Previously reported substrate data are included as reference values to facilitate direct comparison with biotransformation products and to highlight diagnostic changes associated with C=C reduction, glycosylation, methylglucosylation, and *O*-demethylation.

The structure of the reduced product **1A** was confirmed by the disappearance of trans-olefinic proton doublets characteristic of substrate **1** and the appearance of aliphatic methylene signals at δ 3.12–3.16 and 2.83–2.87 ppm. Formation of the methylglucosylated derivative **1B** was supported by the presence of the anomeric proton signal at δ 5.03 ppm and an additional methoxy signal of the sugar moiety at δ 3.46 ppm. *O*-Demethylation leading to **1C** was confirmed by the absence of the C-2-OCH_3_ singlet and the corresponding changes in the aromatic proton pattern.

In contrast, selected strains displayed evidence of competing metabolic pathways. Particularly in *Beauveria bassiana* KCh J1.5 and *Isaria fumosorosea* KCh J2, metabolites tentatively identified as chalcone glycosides retaining the α,β-unsaturated system (**1D**) were detected based on their UV spectral characteristics and chromatographic behavior. Their formation suggests that glycosylation may directly compete with ene-reductase activity and, in these strains, may occur prior to reduction of the activated C=C bond. This effect was especially pronounced in *I. fumosorosea* KCh J2, in which considerable amounts of unreacted chalcone substrate remained detectable even after prolonged cultivation, indicating relatively weak ene-reductase activity compared with highly active glycosylating strains.

The *ortho*-methoxy derivative additionally exhibited the highest susceptibility toward secondary *O*-demethylation reactions among all investigated regioisomers. Formation of demethylated dihydrochalcone derivatives was particularly evident in cultures of *B*. *bassiana* KCh J1 and *Isaria farinosa* KCh KW1.1, where gradual accumulation of more polar metabolites was observed during prolonged incubation. The structure of the major demethylated metabolite was confirmed by preparative isolation followed by NMR spectroscopic analysis. Several strains additionally generated highly polar secondary metabolites displaying UV spectra characteristic of the dihydrochalcone scaffold, suggesting activation of further oxidative and conjugative metabolic pathways following initial reduction and glycosylation steps.

Overall, the metabolic profile of (**1**) indicates that the ortho-methoxy substrate undergoes competing reduction, methylglucosylation, and *O*-demethylation pathways in a strain-dependent manner. Compared with the other regioisomers, it showed a greater tendency toward *O*-demethylation and some secondary transformations, while the overall core biotransformation pathway remained similar.

### 2.3. Biotransformation of the 4′-Hydroxy-3-methoxychalcone

Biotransformation of the meta-methoxy-substituted chalcone (**2**) revealed highly dynamic and strain-dependent cascade metabolism involving early reduction of the α,β-unsaturated system, followed by glycosylation, *O*-demethylation, and formation of secondary polar metabolites. In contrast to the *ortho*-substituted analogue, the parent chalcone signal decreased markedly in most investigated cultures, indicating that *meta*-methoxy substitution favors reduction-oriented metabolism. The suggested metabolic routes of (**2**) are summarized in Scheme 2.

The initial reduction step leading to formation of the corresponding dihydrochalcone represented the dominant metabolic transformation in nearly all investigated strains. Particularly pronounced formation of the reduced product was observed in cultures of *Beauveria bassiana* KCh J1, *Isaria tenuipes* MU35, and *Metarhizium robertsii* MU4, where only trace amounts or no detectable parent substrate signal were observed after 24 h of cultivation. In several strains, transient accumulation of the intermediate dihydrochalcone was subsequently followed by pronounced glycosylation tendency, leading to formation of methylglucosylated dihydrochalcones as dominant late-stage metabolites.

The most pronounced methylglucosylation-oriented profile was observed for *B. bassiana* KCh J1.5, *B. bassiana* KCh BBT, and *B. caledonica* KCh J3.3. In these strains, methylglucosylated dihydrochalcones became the predominant metabolites after 3 days of cultivation, whereas intermediate dihydrochalcones were either absent or present only in trace amounts. Particularly in *B. bassiana* KCh J1.5, prolonged cultivation additionally resulted in accumulation of highly polar metabolites displaying UV spectra characteristic of the dihydrochalcone scaffold, suggesting activation of further oxidative or conjugative metabolic pathways following glycosylation.

Several fungal strains additionally exhibited pronounced *O*-demethylation activity. The pronounced *O*-demethylation profile was observed in cultures of *Isaria farinosa* KCh KW1.1 and *B. bassiana* KCh J1, where gradual accumulation of demethylated dihydrochalcone derivatives occurred during prolonged incubation. In *I. farinosa* KCh KW1.1, the relative abundance of the demethylated metabolite increased between days 1 and 3 and was accompanied by formation of glycosylated derivatives during later cultivation stages. Furthermore, several highly polar metabolites displaying UV spectra similar to those of dihydrochalcones were observed in cultures of *M. robertsii* MU4, *I. fumosorosea* KCh J2, and *I. tenuipes* MU35, indicating development of complex secondary metabolism following initial reduction and glycosylation reactions. ^13^C NMR data of the parent substrate and the major metabolites are presented in Table 2.

4′-hydroxy-3-methoxychalcone (**2**): ^1^H NMR (600 MHz, DMSO-*d*_6_) δ (ppm): 10.43 (s, 1H, C-4′-OH), 8.04–8.12 (m, 2H, H-2′ and H-6′), 7.92 (d, 1H, J = 15.6 Hz, H-α), 7.65 (d, 1H, J = 15.6 Hz, H-β), 7.46 (t, 1H, J = 1.9 Hz, H-2), 7.41 (d, 1H, J = 7.7 Hz, H-6), 7.36 (t, 1H, J = 7.8 Hz, H-5), 7.01 (ddd, 1H, J = 8.0, 2.4, 0.8 Hz, H-4), 6.88–6.92 (m, 2H, H-3′ and H-5′), 3.83 (s, 3H, C-3-OCH_3_).

4′-hydroxy-3-methoxydihydrochalcone (**2A**): ^1^H NMR (600 MHz, DMSO-*d*_6_) δ (ppm): 10.36 (s, 1H, C-4′-OH), 7.84–7.88 (m, 2H, H-2′ and H-6′), 7.17 (t, 1H, J = 7.9 Hz, H-5), 6.81–6.85 (m, 4H, H-2, H-6, H-3′ and H-5′), 6.73 (ddd, 1H, J = 8.0, 2.4, 0.8 Hz, H-4), 3.72 (s, 3H, C-3-OCH_3_), 3.21–3.25 (m, 2H, H-α), 2.86–2.89 (m, 2H, H-β). HRESI-MS: [M + H]^+^; *m*/*z* 257.1181 (calcd. 257.1172; mass error = +3.4 ppm).

4′-*O*-β-D-(4″-*O*-methylglucopyranosyl)-3-methoxydihydrochalcone (**2B**): ^1^H NMR (600 MHz, DMSO-*d*_6_) δ (ppm): 7.93–7.98 (m, 2H, H-2′ and H-6′), 7.18 (t, 1H, J = 7.9 Hz, H-5), 7.08–7.12 (m, 2H, H-3′ and H-5′), 6.82–6.86 (m, 2H, H-2 and H-6), 6.74 (ddd, 1H, J = 8.1, 2.4, 0.8 Hz, H-4), 5.46 (d, 1H, *J* = 5.3 Hz, C-2″-OH), 5.29 (d, 1H, *J* = 5.5 Hz, C-3″-OH), 5.03 (d, 1H, *J* = 7.8 Hz, C-1″), 4.71 (dd, 1H, *J* = 6.3, 5.1 Hz, C-6″-OH), 3.72 (s, 3H, C-3-OCH_3_), 3.62 (ddd, 1H, *J* = 11.5, 4.8, 1.5 Hz, one of C-6″), 3.50 (ddd, 1H, *J* = 11.6, 6.4, 4.9 Hz, one of C-6″), 3.46 (s, 3H, C-4″-OCH_3_), 3.39–3.45 (m, 2H, C-3″ and C-5″), 3.28–3.34 (m, 2H, H-α), 3.27 (ddd, *J* = 9.0, 7.9, 5.2 Hz, C-2″), 3.05 (t, 1H, *J* = 9.3 Hz, C-4″), 2.86–2.92 (m, 2H, H-β). HRESI-MS: [M + H]^+^; *m*/*z* 433.1863 (calcd. 433.1857; mass error = +1.4 ppm).

3,4′-dihydroxydihydrochalcone (**2C**): ^1^H NMR (600 MHz, Acetone-*d*_6_) δ (ppm): 7.91–7.94 (m, 2H, H-2′ and H-6′), 7.08 (t, 1H, J = 7.9 Hz, H-5), 6.90–6.93 (m, 2H, H-3′ and H-5′), 6.76 (br s, 1H, H-2), 6.75 (d, 1H, *J* = 7.5 Hz, H-6), 6.65 (dd, 1H, *J* = 8.0, 2.4, Hz, H-4), 3.20–3.25 (m, 2H, H-α), 2.90–2.93 (m, 2H, H-β). HRESI-MS: [M + H]^+^; *m*/*z* 243.1018 (calcd. 243.1016; mass error = +0.9 ppm).

Interestingly, unlike 4′-hydroxy-2-methoxychalcone (**1**), the *meta-*substituted substrate showed markedly lower persistence of the chalcone form during cultivation, while no substantial accumulation of putative chalcone glycosides retaining the α,β-unsaturated system was observed. This observation is consistent with a greater relative tendency toward ene-reduction than direct chalcone glycosylation for the meta-substituted substrate under the applied conditions. Simultaneously, the broad diversity of secondary metabolites detected during prolonged cultivation demonstrates that *meta*-substituted hydroxychalcones undergo highly complex and competing cascade biotransformation pathways in cultures of entomopathogenic fungi.

### 2.4. Biotransformation of the 4′-Hydroxy-4-methoxychalcone

Biotransformation of the *para*-methoxy-substituted chalcone followed the same general cascade pattern observed for the other regioisomers, involving early reduction of the α,β-unsaturated system followed by methylglucosylation of the resulting dihydrochalcone. The suggested metabolic routes of **3** are summarized in Scheme 3.

Thus, the major metabolic route led from the parent chalcone to 4′-hydroxy-4-methoxydihydrochalcone and subsequently to 4′-*O*-β-D-(4″-*O*-methylglucopyranosyl)-4-methoxydihydrochalcone. This confirms that, despite differences in the relative abundance of individual metabolite classes, ene-reduction followed by methylglucosylation represents a common transformation pathway for hydroxy-methoxychalcones in cultures of entomopathogenic fungi. The major products and representative secondary metabolites formed from the *para*-substituted substrate were characterized by NMR spectroscopy, as detailed below.

Among the investigated fungal strains, the *para*-methoxy-substituted substrate showed a clear tendency toward early double bond reduction followed by strain-dependent glycosylation and secondary metabolism. Numerical ratios given below refer exclusively to uncorrected relative DAD peak areas of closely related metabolites exhibiting comparable spectral profiles; they do not represent absolute concentrations or molar product distributions. In *Beauveria bassiana* KCh J1, the corresponding dihydrochalcone (**3A**) was the only detectable product after 24 h. During prolonged incubation, a demethylated dihydrochalcone derivative gradually accumulated. After 10 days, the uncorrected relative DAD peak–area ratio of **3A** to the demethylated dihydrochalcone was approximately 71:29, with 3A remaining the predominant product. This indicates that in this strain *O*-demethylation represents a secondary transformation occurring after efficient reduction of the α,β-unsaturated system.

A markedly different profile was observed for *Beauveria bassiana* KCh J1.5, where only glycosylated reduced products were detected from the first day of biotransformation. The methylglucosylated dihydrochalcone (**3B**) and a second glycosylated dihydrochalcone (possibly **3D**) showed uncorrected relative DAD peak–area ratios of approximately 66:34 after 24 h and 59:41 from day 3 onward, with no major changes during further cultivation. This indicates that conjugation of the reduced intermediate had already occurred by the first 24-h sampling point and that relatively limited secondary metabolism followed during prolonged cultivation. In *Beauveria caledonica* KCh J3.3, residual *cis*- and *trans*-chalcone forms were still visible after 24 h, although the dihydrochalcone was already the predominant metabolite and methylglucosylated product was also detected. From day 3, the methylglucosylated dihydrochalcone became the main product, and the metabolic profile remained largely unchanged after 7 and 10 days.

The remaining strains generated more heterogeneous profiles. In *Isaria tenuipes* MU35, the dihydrochalcone predominated during the initial stage, accompanied by residual cis- and trans-substrate forms. After 3 days, the substrate signal was no longer evident, while only minor amounts of demethylated and methylglucosylated dihydrochalcone derivatives were observed; both metabolite classes increased slightly during prolonged incubation. *Metarhizium robertsii* MU4 also produced the dihydrochalcone as the major early metabolite, together with residual substrate, demethylated product, methylglucosylated dihydrochalcone, and a more polar compound displaying UV characteristics consistent with a chalcone-type scaffold. After 3 days, methylglucosylated dihydrochalcone became the dominant product, whereas after 7 and 10 days the methylglucosylated metabolite (**3B**) and the more polar derivative showed an uncorrected relative DAD peak–area ratio of approximately 78:22.

In *Beauveria bassiana* KCh BBT, the reduced aglycone predominated after 24 h, while methylglucosylated dihydrochalcone became the major product after 3 days. A more polar metabolite with UV characteristics of a dihydrochalcone scaffold was also detected, and the profile remained stable during further incubation. In *Isaria farinosa* KCh KW1.1, the dihydrochalcone was the dominant early product, accompanied by residual cis- and trans-substrate forms. After 3 days, the reduced aglycone (**3A**) still predominated, while minor amounts of a demethylated product and the methylglucosylated dihydrochalcone (**3B**), together with traces of a putative chalcone glycoside (**3C**), were observed. During prolonged cultivation, methylglucosylated dihydrochalcone became the main product, indicating delayed but efficient conjugation. In *Isaria fumosorosea* KCh J2, the early profile contained residual substrate, predominant dihydrochalcone, and a small amount of methylglucosylated dihydrochalcone. From day 3, the methylglucosylated derivative became the major metabolite, while a more polar glycosidic derivative appeared after 7 days and increased slightly after 10 days, although the methylglucosylated dihydrochalcone remained the dominant product.

Overall, the metabolic profile of 4′-hydroxy-4-methoxychalcone shows that *para*-methoxy substitution favors efficient reduction of the α,β-unsaturated system followed by accumulation of methylglucosylated dihydrochalcone derivatives. Direct glycosylation of the unreduced chalcone was only marginally observed, whereas secondary transformations, including *O*-demethylation and formation of more polar glycosidic or dihydrochalcone-like metabolites, were strongly strain-dependent. Thus, compared with the *ortho-* and *meta*-methoxy analogues, the *para*-substituted substrate showed a more consistent reduction–methylglucosylation pathway, while prolonged cultivation revealed additional metabolic plasticity in selected fungal cultures.

4′-hydroxy-4-methoxychalcone (**3**): ^1^H NMR (600 MHz, DMSO-*d*_6_) δ (ppm): 10.41 (s, 1H, C-4′-OH), 8.03–8.06 (m, 2H, H-2′ and H-6′), 7.79–7.83 (m, 2H, H-2 and H-6), 7.76 (d, 1H, *J* = 15.5 Hz, H-α), 7.65 (d, 1H, *J* = 15.5 Hz, H-β), 6.98–7.02 (m, 2H, H-3 and H-5), 6.87–6.91 (m, 2H, H-3′ and H-5′), 3.81 (s, 3H, C-4-OCH_3_).

4′-hydroxy-4-methoxydihydrochalcone (**3A**): ^1^H NMR (600 MHz, CDCl_3_) δ (ppm): 7.87–7.91 (m, 2H, H-2′ and H-6′), 7.13–7.17 (m, 2H, H-2 and H-6), 7.00 (s, 1H, C-4′-OH), 6.87–6.91 (m, 2H, H-3′ and H-5′), 6.82–6.85 (m, 2H, H-3 and H-5), 3.78 (s, 3H, C-4-OCH_3_), 3.20–3.24 (m, 2H, H-α), 2.92–3.02 (m, 2H, H-β). HRESI-MS: [M + H]^+^; *m*/*z* 257.1176 (calcd. 257.1172; mass error = +1.5 ppm).

4′-*O*-β-D-(4″-*O*-methylglucopyranosyl)-4-methoxydihydrochalcone (**3B**): ^1^H NMR (600 MHz, Acetone-*d*_6_) δ (ppm): 7.93–7.99 (m, 2H, H-2′ and H-6′), 7.18–7.21 (m, 2H, H-2 and H-6), 7.09–7.14 (m, 2H, H-3′ and H-5′), 6.81–6.85 (m, 2H, H-3 and H-5), 5.06 (d, 1H, *J* = 7.8 Hz, C-1″), 3.84 (dd, 1H, *J* = 12.0, 1.8 Hz, one of C-6″), 3.75 (s, 3H, C-4-OCH_3_), 3.67–3.71 (m, 1H, one of C-6″), 3.65 (t, 1H, *J* = 9.1 Hz, C-3″), 3.56 (s, 3H, C-4″-OCH_3_), 3.50–3.54 (m, 1H, C-5″), 3.50 (dd, 1H, *J* = 9.1, 7.8 Hz, C-2″), 3.24–3.28 (m, 2H, H-α), 3.22 (dd, 1H, *J* = 9.5, 9.1 Hz, C-4″), 2.91–2.95 (m, 2H, H-β).

4′-*O*-β-D-(4″-*O*-methylglucopyranosyl)-4-methoxychalcone (**3C**): ^1^H NMR (600 MHz, Acetone-*d*_6_) δ (ppm): 8.10–8.14 (m, 2H, H-2′ and H-6′), 7.76–7.80 (m, 2H, H-2 and H-6), 7.75 (d, 1H, J = 15.5 Hz, H-α), 7.74 (d, 1H, J = 15.5 Hz, H-β), 7.16–7.18 (m, 2H, H-3′ and H-5′), 6.99–7.02 (m, 2H, H-3 and H-5), 5.09 (d, 1H, *J* = 7.8 Hz, C-1″), 3.86 (s, 3H, C-4-OCH_3_), 3.84 (dd, 1H, *J* = 12.0, 1.8 Hz, one of C-6″), 3.67–3.71 (m, 1H, one of C-6″), 3.65 (t, 1H, *J* = 9.1 Hz, C-3″), 3.56 (s, 3H, C-4″-OCH_3_), 3.50–3.54 (m, 1H, C-5″), 3.50 (dd, 1H, *J* = 9.1, 7.8 Hz, C-2″), 3.22 (dd, 1H, *J* = 9.5, 9.1 Hz, C-4″). HR-ESI-QTOF-MS analysis of the enriched fraction containing **3B** and **3C** showed protonated molecular ions at *m*/*z* 433.1863 for **3B** (calcd. 433.1857; mass error = +1.4 ppm) and *m*/*z* 431.1706 for **3C** (calcd. 431.1700; mass error = +1.3 ppm), respectively.

4′-*O*-β-D-(4″-*O*-methylglucopyranosyl)-4-hydroxydihydrochalcone (**3D**): ^1^H NMR (600 MHz, Acetone-*d*_6_) δ (ppm): 7.94–8.00 (m, 2H, H-2′ and H-6′), 7.10–7.13 (m, 2H, H-3′ and H-5′), 7.08–7.11 (m, 2H, H-2 and H-6), 6.72–6.76 (m, 2H, H-3 and H-5), 5.06 (d, 1H, *J* = 7.8 Hz, C-1″), 3.83 (dd, 1H, *J* = 11.9, 1.9 Hz, one of C-6″), 3.68 (dd, 1H, *J* = 11.8, 4.8 Hz, one of C-6″), 3.64 (t, 1H, *J* = 9.0 Hz, C-3″), 3.56 (s, 3H, C-4″-OCH_3_), 3.52 (ddd, 1H, *J* = 11.3, 5.6, 2.7 Hz, C-5″), 3.49 (dd, 1H, *J* = 9.1, 7.8 Hz, C-2″), 3.21–3.25 (m, 2H, H-α), 3.22 (dd, 1H, *J* = 9.5, 9.1 Hz, C-4″), 2.86–2.91 (m, 2H, H-β).

4′-O-β-D-(4″-O-methylglucopyranosyl)-4-methoxydihydrochalc-1-ol (**3E**) or 1-(4′-O-β-D-(4″′-O-methylglucopyranosylphenyl)-3-(4″-methoxyphenyl)-propan-1-ol (**3E**): ^1^H NMR (600 MHz, Acetone-*d*_6_) δ (ppm): 7.26–7.29 (m, 2H, H-2′ and H-6′), 6.99–7.02 (m, 2H, H-2 and H-6), 6.99–7.02 (m, 2H, H-3′ and H-5′), 6.81–6.83 (m, 2H, H-3 and H-5), 4.91 (d, 1H, *J* = 7.8 Hz, C-1″), 4.61 (dd, 1H, *J* = 7.7, 5.2 Hz, C*H*-OH), 3.82 (dd, 1H, *J* = 12.0, 1.8 Hz, one of C-6″), 3.74 (s, 3H, C-4-OCH_3_), 3.67–3.71 (m, 1H, one of C-6″), 3.62 (t, 1H, *J* = 9.0 Hz, C-3″), 3.56 (s, 3H, C-4″-OCH_3_), 3.50–3.54 (m, 1H, C-5″), 3.44 (dd, 1H, *J* = 9.1, 7.8 Hz, C-2″), 3.21 (dd, 1H, *J* = 9.5, 9.1 Hz, C-4″), 2.66 (ddd, 1H, *J* = 13.8, 9.3, 4.7 Hz, one of H-β), 2.58 (ddd, 1H, *J* = 13.8, 9.8, 6.5 Hz, one of H-β), 1.94–2.01 (m, 1H, one of H-α), 1.87–1.93 (m, 1H, one of H-α).

4-*O*-β-D-(4″-*O*-methylglucopyranosyl)-4′-hydroxydihydrochalcone (**3F**): ^1^H NMR (600 MHz, Acetone-*d*_6_) δ (ppm): 7.88–7.94 (m, 2H, H-2′ and H-6′), 7.18–7.20 (m, 2H, H-2 and H-6), 6.94–6.97 (m, 2H, H-3 and H-5), 6.89–6.92 (m, 2H, H-3′ and H-5′), 4.87 (d, 1H, *J* = 7.8 Hz, C-1″), 3.82 (dd, 1H, *J* = 12.0, 1.8 Hz, one of C-6″), 3.67–3.71 (m, 1H, one of C-6″), 3.60 (t, 1H, *J* = 9.0 Hz, C-3″), 3.55 (s, 3H, C-4″-OCH_3_), 3.50–3.54 (m, 1H, C-5″), 3.43 (dd, 1H, *J* = 9.1, 7.8 Hz, C-2″), 3.21–3.24 (m, 2H, H-α), 3.20 (dd, 1H, *J* = 9.5, 9.1 Hz, C-4″), 2.91–2.95 (m, 2H, H-β). HR-ESI-QTOF-MS analysis of the enriched fraction containing **3D**–**3F** showed an ion at *m*/*z* 419.1702 ([M + H]^+^; calcd. 419.1700; mass error = +0.4 ppm), supporting the elemental composition shared by the proposed isomeric metabolites **3D** and **3F**. Compound **3E** was additionally supported by the sodium adduct [M + Na]^+^ at *m*/*z* 457.1838 (calcd. 457.1833; mass error = +1.1 ppm). Because **3D**–**3F** were obtained as an enriched multicomponent fraction, these HRMS data support the proposed elemental compositions but do not establish the individual purity of the metabolites or distinguish between the isomeric structures **3D** and **3F**.

The structures of the major products from the *para*-methoxy series were confirmed by NMR spectroscopy. The reduced derivative **3A** was identified by disappearance of the trans-olefinic proton signals and appearance of aliphatic H-α/H-β resonances. The methylglucosylated derivative **3B** showed an anomeric proton signal at δ 5.06 ppm and an additional methoxy signal assigned to the 4″-*O*-methylglucopyranosyl moiety. The chalcone glycoside **3C** retained the trans-olefinic proton system, indicating that glycosylation may also occur before reduction of the activated C=C bond.

In the screening experiment, further transformation of the methylglucosylated dihydrochalcone (**3B**) was observed only in selected fungal strains, whereas in others this metabolite remained the dominant late-stage product. This indicates that the ability to metabolize methylglucosylated dihydrochalcones further is strain-dependent and not a universal feature of all tested entomopathogenic fungi. To investigate the structural nature of these secondary products, an additional preparative experiment was performed using the culture of *Beauveria caledonica* KCh J3.3 after 10 days of incubation. The resulting enriched fraction contained three closely related components, tentatively assigned as **3D**, **3E**, and **3F**. These metabolites could not be separated as individual pure products under the applied preparative TLC conditions. Their tentative structural assignments were based on diagnostic ^1^H and ^13^C NMR signals supported by 2D NMR correlations. This metabolic sequence, involving initial *O*-demethylation followed by methylglucosylation, has previously been observed and described for a related substrate [19]. Compound **3D** retained the dihydrochalcone carbonyl system and lacked the aromatic C-4 methoxy group, indicating *O*-demethylation. Compound **3E** showed signals consistent with reduction of the carbonyl group to the corresponding dihydrochalc-1-ol. This transformation is plausible, as a similar reduction of the carbonyl group to the corresponding alcohol has previously been observed for other structurally related compounds [23]. Compound **3F** was assigned as an alternative glycosylated 4′-hydroxydihydrochalcone derivative based on the presence of sugar resonances and dihydrochalcone H-α/H-β signals. ^13^C NMR data is presented in Table 3.

Taken together, the biotransformation profile of the *para*-methoxy substrate confirms that ene-reduction followed by methylglucosylation represents the common core pathway for the investigated 4′-hydroxymethoxychalcones (**3**). At the same time, the strain-dependent secondary transformation of methylglucosylated dihydrochalcones demonstrates that these conjugates are not necessarily terminal metabolites in entomopathogenic fungal cultures. Depending on the fungal strain, they may either accumulate as dominant late-stage products or undergo further enzymatic modification, including *O*-demethylation, carbonyl reduction, and alternative glycosylation-related transformations. Thus, the *para*-methoxy derivative follows the same general transformation sequence as the other regioisomers, while prolonged cultivation reveals additional metabolic plasticity of selected fungal whole-cell systems.

Although the three regioisomeric substrates differed in the position of the methoxy group on ring B, their major biotransformation pathways were broadly similar. In all cases, reduction of the α,β-unsaturated bond constituted the predominant primary transformation and was frequently followed by glycosylation of the 4′-hydroxy group. Thus, the position of the methoxy substituent does not appear to determine the overall metabolic pathway, but rather to modulate the relative efficiency of individual steps and the formation of minor secondary metabolites.

The most direct effect may concern ene-reduction, because the methoxy group is located on ring B conjugated with the α,β-unsaturated carbonyl system. Consistent with this interpretation, measurable differences were observed in the NMR parameters of the enone moiety. In the ortho-methoxy substrate **1**, H-α and H-β resonated at δ 7.85 and 7.99 ppm, respectively, whereas the corresponding signals occurred at δ 7.92 and 7.65 ppm for the meta isomer **2**, and at δ 7.76 and 7.65 ppm for the para isomer **3**. Differences were also evident in the corresponding ^13^C NMR signals: C-β appeared at δ 137.29 ppm for **1**, compared with δ 142.75 and 142.80 ppm for **2** and **3**, respectively, while C-α resonated at δ 121.91, 122.37, and 119.64 ppm, respectively. All three substrates retained the characteristic trans coupling constants (J_αβ_ = 15.5–15.8 Hz), indicating that these differences reflect changes in the local electronic and/or conformational environment of the conjugated enone system rather than differences in alkene geometry. Such position-dependent effects of methoxy substitution on the electronic properties of conjugated chalcone systems have also been reported in structure–property studies [48,49].

These differences may influence substrate recognition and reduction by fungal ene-reductases; however, the present whole-cell experiments do not allow the contribution of electronic effects to be separated from conformational and enzyme-recognition effects. In contrast, the subsequent glycosylation occurs predominantly at the 4′-hydroxy group located on the opposite aromatic ring, and the present results do not indicate a pronounced direct effect of methoxy position on this transformation. More evident regioisomer-dependent differences were observed mainly in minor secondary pathways, including *O*-demethylation and formation of additional polar metabolites.

## 3. Materials and Methods

### 3.1. Substrates

Three 4′-hydroxymethoxychalcones differing solely in the position of the methoxy group on ring B were used as substrates: 4′-hydroxy-2-methoxychalcone (**1**), 4′-hydroxy-3-methoxychalcone (**2**), and 4′-hydroxy-4-methoxychalcone (**3**). The substrates were synthesized by Claisen–Schmidt condensation of 4′-hydroxyacetophenone with the corresponding methoxy-substituted benzaldehydes, following our previously reported procedure for 4′-hydroxychalcone [47]. Briefly, equimolar amounts of 4′-hydroxyacetophenone and the appropriate methoxy-substituted benzaldehyde were dissolved in methanol, followed by the addition of water and NaOH. The reaction mixture was heated under reflux, and reaction progress was monitored by TLC. After completion, the reaction mixture was poured into a beaker containing an appropriate volume of 0.1 M HCl to acidify the medium and precipitate the crude chalcone product. The precipitate was collected by filtration, washed with distilled water until neutral pH, dried, and purified by recrystallization from ethanol.

The structures and chemical integrity of substrates 1–3 were confirmed by ^1^H and ^13^C NMR spectroscopy, as reported previously [47]. The corresponding spectra were presented in the Supplementary Materials of that publication. The chromatographic purity of the substrates used in the present biotransformation experiments was verified by UHPLC-DAD prior to testing. Only compounds of confirmed identity and high purity were used for screening and preparative-scale biotransformations. Stock solutions of substrates were prepared in DMSO immediately before addition to fungal cultures. Substrate solutions were protected from direct light before addition to fungal cultures. All reagents were purchased from Sigma-Aldrich (St. Louis, MO, USA) unless otherwise stated.

### 3.2. Microorganisms

The entomopathogenic fungal strains *Beauveria caledonica* KCh J3.3, *Beauveria bassiana* KCh J1.5, *B. bassiana* KCh J1, *B. bassiana* KCh BBT, *Isaria farinosa* KCh KW1.1, *I. fumosorosea* KCh J2, *I. tenuipes* MU35, and *Metarhizium robertsii* MU4 were obtained from the collection of the Department of Food Chemistry and Biocatalysis, Wrocław University of Environmental and Life Sciences (Wrocław, Poland). The strains were maintained on Sabouraud agar slants at 4 °C and transferred to fresh medium before use.

### 3.3. Screening Procedure

Screening biotransformations were performed as an exploratory metabolic profiling step to compare the transformation tendencies of the selected entomopathogenic fungal strains and to select strain–substrate systems for preparative-scale experiments. Erlenmeyer flasks (300 mL), each containing 100 mL of sterile cultivation medium composed of 3% glucose and 1% Aminobac (BTL Sp. z.o.o., Łódź, Poland), were inoculated with a suspension of each entomopathogenic strain and incubated for 4 days at 25 °C on a rotary shaker at 130 rpm. After this pre-cultivation period, 10 mg of substrate dissolved in 1 mL of dimethyl sulfoxide (DMSO) was added to each culture. The final concentration of DMSO in the screening cultures was 1% (*v*/*v*). Samples were collected after 1, 3, 7, and 10 days of biotransformation.

The sampling schedule was designed for comparative screening of early, intermediate, and late metabolic profiles across multiple substrate–strain combinations rather than for detailed kinetic characterization of the initial ene-reduction step. Accordingly, 24 h was the earliest sampling point. The present experiments therefore allow us to conclude that dihydrochalcone formation was already evident at 24 h in many cultures, but they do not permit determination of the exact onset of reduction or the time at which the maximum concentration of the reduced intermediate was reached. Determination of the detailed kinetics of the initial reduction step would require a separate experiment with substantially shorter sampling intervals during the first 24 h. The samples were extracted with ethyl acetate, and the organic extracts were dried over anhydrous MgSO_4_, concentrated in vacuo, and analyzed by TLC and UHPLC-DAD.

The screening results were interpreted primarily qualitatively on the basis of substrate disappearance, chromatographic retention behavior, DAD spectral characteristics, and comparison with isolated NMR-characterized products when available. Metabolites showing retention times and DAD spectra consistent with those of isolated reference products were assigned accordingly, whereas additional signals were tentatively classified at the metabolite-class level. Because compound-specific calibration curves and response factors were not available for all detected metabolites, absolute substrate conversion and product yields could not be calculated from the UHPLC-DAD data. Semi-quantitative peak–area ratios were therefore used only for closely related metabolites displaying comparable DAD spectral profiles. The screening stage was designed for comparative metabolic profiling and selection of strain–substrate systems for preparative-scale experiments rather than for quantitative determination of biotransformation yields.

### 3.4. Preparative Separation of Biotransformation Products

Preparative-scale biotransformations were performed for selected strain–substrate systems in order to obtain sufficient amounts of major metabolites for structural identification. Erlenmeyer flasks (2000 mL), each containing 500 mL of the same cultivation medium composed of 3% glucose and 1% Aminobac, were inoculated as described above and incubated for 4 days at 25 °C on a rotary shaker at 130 rpm. After this pre-cultivation period, 100 mg of substrate dissolved in 2 mL of DMSO was added to each culture. The final concentration of DMSO in preparative cultures was 0.4% (*v*/*v*). Samples were periodically collected between days 3 and 7 to monitor metabolite formation by TLC and UHPLC-DAD. After completion of the process, the cultures were extracted three times with ethyl acetate. The combined organic extracts were dried over anhydrous MgSO_4_, concentrated in vacuo, and subjected to chromatographic separation. The obtained fractions were analyzed by UHPLC-DAD and NMR spectroscopy, including ^1^H NMR, ^13^C NMR, COSY, HSQC, and HMBC experiments.

The crude extracts obtained from preparative-scale biotransformations were separated using preparative TLC on 500 µm silica gel plates (Anatech, Gehrden, Germany). A chloroform:methanol mixture, 9:1 (*v*/*v*), was used as the mobile phase. After chromatographic development, the separated zones were visualized under UV light, scraped off, and extracted twice with ethyl acetate. The obtained fractions were concentrated in vacuo and analyzed by UHPLC-DAD and NMR spectroscopy. After solvent evaporation, the isolated fractions were used directly for spectroscopic characterization and were not subjected to crystallization or recrystallization. Accordingly, melting-point measurements were not included in the original experimental design, as the objective of the preparative experiments was structural elucidation of the metabolites and characterization of the biotransformation capacity of the fungal strains rather than optimization of product crystallization or solid-state properties. Fractions containing major metabolites were used for structural assignment when their chromatographic and spectroscopic profiles were sufficiently informative. Preparative TLC provided sufficient resolution for isolation of major metabolites when their polarity differences were pronounced. However, closely related glycosylated derivatives frequently exhibited very similar chromatographic mobility and could not always be completely resolved under the applied conditions. The purity and composition of the collected zones were therefore evaluated by UHPLC-DAD and NMR spectroscopy. Fractions containing a single predominant metabolite with sufficiently resolved chromatographic and spectroscopic characteristics were treated as individually isolated products, whereas fractions containing co-migrating closely related metabolites were explicitly described as enriched multicomponent fractions. In particular, metabolites **3D**–**3F** were obtained together in an enriched fraction and were not isolated as individual pure compounds.

### 3.5. UHPLC

UHPLC-DAD analyses were carried out using a Thermo Scientific Dionex Ultimate 3000 UHPLC+ system (Thermo Fisher Scientific, Waltham, MA, USA) equipped with a photodiode array detector. UV spectra were recorded in the range of 210–450 nm. Chromatographic separation was performed on a ZORBAX Eclipse XDB-C18 analytical column (5 µm, 4.6 × 250 mm; Agilent, Santa Clara, CA, USA). The mobile phases consisted of A: 0.1% HCOOH in H_2_O and B: 0.1% HCOOH in acetonitrile. The following gradient program was used: 0–0.5 min, 25% B; 4.0 min, 40% B; 7.5 min, 55% B; 8.0 min, 100% B; 9.7 min, 25% B; and 11.0 min, 25% B. The flow rate was 0.7 mL min^−1^. Metabolite classes were assigned on the basis of retention behavior, UV spectral characteristics, comparison with substrate controls, and NMR-confirmed structures of isolated products when available.

Because reduction of the α,β-unsaturated bond substantially changes the chromophore, the parent chalcones and the corresponding dihydrochalcones exhibited different DAD spectra and wavelength-dependent detector responses. Therefore, peak areas of compounds belonging to these two chromophore classes were not directly compared and were not used to calculate substrate conversion, absolute product distribution, or biotransformation yield. Substrate disappearance was evaluated from the absence of the corresponding *cis*- and *trans*-chalcone peaks under the applied chromatographic conditions, whereas metabolite formation was assessed on the basis of retention behavior, DAD spectral characteristics, and comparison with isolated NMR-characterized products where available. Semi-quantitative peak–area ratios were reported only for closely related metabolites displaying comparable DAD spectral profiles. These values represent uncorrected relative detector–response ratios and should not be interpreted as molar ratios or absolute concentrations.

### 3.6. NMR Data of Biotransformation Products

NMR spectra of isolated or enriched biotransformation products were recorded on a Bruker Avance spectrometer (Bruker, Rheinstetten, Germany) operating at 600 MHz for ^1^H NMR and 151 MHz for ^13^C NMR. Samples were dissolved in DMSO-d_6_, CDCl_3_, or acetone-d_6_, depending on solubility. Chemical shifts are reported in ppm relative to residual solvent signals. Structural assignments were based on ^1^H NMR, ^13^C NMR, COSY, HSQC, and HMBC experiments. Diagnostic NMR signals and 2D correlations were used to confirm reduction, glycosylation, methylglucosylation, and *O*-demethylation of the chalcone scaffold. NMR spectra of newly identified or newly characterized biotransformation products are provided in the Supplementary Materials (Figures S1–S30). Spectra of the parent substrates and previously reported simple dihydrochalcone products are not duplicated in the present Supplementary Materials, as they were reported in our previous publication [47]; their NMR data are included in the main text and comparative ^13^C NMR tables only to facilitate direct structural comparison with the newly obtained methylglucosylated, *O*-demethylated, and secondary metabolites.

### 3.7. UHPLC–HR-ESI-QTOF-MS Analysis

High-resolution mass spectrometric analyses were performed using a Waters ACQUITY UPLC H-Class system equipped with an autosampler and coupled to a Xevo G3 QTof mass spectrometer equipped with an electrospray ionization (ESI) source (Waters, Milford, MA, USA). Chromatographic separation was performed using an ACQUITY UPLC BEH C8 column (2.1 × 50 mm, 1.7 µm; Waters, Milford, MA, USA) maintained at 40 °C. The mobile phase consisted of 0.1% formic acid in water (solvent A) and 0.1% formic acid in acetonitrile (solvent B). The total analysis time was 6 min. The gradient elution program was as follows: 10% B from 0 to 1 min, a linear increase from 10% to 90% B from 1 to 3 min, 90% B from 3 to 4.5 min, followed by a decrease from 90% to 10% B from 4.50 to 4.51 min. The flow rate was 400 µL min^−1^, and the injection volume was 1 µL.

Mass spectrometric data were acquired in full-scan sensitivity mode using both positive and negative electrospray ionization. In positive-ion mode (ESI+), the capillary voltage was set to 3.0 kV and the sampling cone voltage to 40 V. The source temperature was 120 °C, the desolvation temperature was 250 °C, the desolvation gas flow rate was 600 L h^−1^, and the cone gas flow rate was 40 L h^−1^. The mass range was *m*/*z* 50–1800. Leucine enkephalin (50 pg µL^−1^) was used as the lock-mass compound and introduced through the LockSpray interface at a flow rate of 10 µL min^−1^, using *m*/*z* 556.2771 as the reference ion.

In negative-ion mode (ESI−), the capillary voltage was set to 2.5 kV and the sampling cone voltage to 40 V. The source temperature was 120 °C, the desolvation temperature was 250 °C, the desolvation gas flow rate was 600 L h^−1^, and the cone gas flow rate was 40 L h^−1^. Leucine enkephalin (50 pg µL^−1^) was introduced through the LockSpray interface at a flow rate of 10 µL min^−1^, using *m*/*z* 554.2615 as the reference ion. Data acquisition was performed using MassLynx software (version 4.1, Waters, Milford, MA, USA).

Accurate-mass measurements were used to support the elemental compositions assigned to the isolated products and components of enriched metabolite fractions. The corresponding HR-ESI-QTOF-MS data and spectra are provided in Table S1 and Figures S31–S39, respectively.

## 4. Conclusions

This study demonstrates that entomopathogenic filamentous fungi are versatile whole-cell biocatalysts capable of carrying out cascade biotransformations of 4′-hydroxymethoxychalcones. Although the tested substrates differed only in the position of the methoxy group on the B-ring, this structural difference had a moderate effect on pathway selectivity, particularly on the extent of ene-reduction and secondary metabolism, while the major biotransformation sequence remained similar across all three regioisomers. For all regioisomers, the main and most common transformation was reduction of the α,β-unsaturated carbonyl system, supporting the pronounced tendency of hydroxychalcones to undergo fungal ene-reduction. In contrast, further metabolic steps, including methylglucosylation, *O*-demethylation, and the formation of more polar secondary metabolites, depended strongly on both the substrate structure and the fungal strain used.

The *ortho*-methoxy derivative showed the highest tendency toward *O*-demethylation, accompanied by competing secondary transformations. These included the formation of demethylated dihydrochalcones and, in selected strains, putative chalcone glycosides in which the α,β-unsaturated system remained unchanged. The *meta*-methoxy analogue showed marked depletion of the parent substrate signal in most fungal cultures, but its metabolic profiles were more diverse, indicating competition between reduction, glycosylation, *O*-demethylation, and further secondary metabolism. In contrast, the *para*-methoxy isomer followed the most consistent metabolic pathway, mainly involving ene-reduction followed by methylglucosylation of the resulting dihydrochalcone, although some strains also promoted additional modifications of glycosylated intermediates.

Overall, these results indicate that methoxy position acts as a modulating rather than pathway-determining structural factor: the major reduction–glycosylation sequence remained broadly conserved, whereas the extent of ene-reduction and secondary transformations varied among the regioisomers and fungal strains. The study also highlights the metabolic flexibility of entomopathogenic fungi, especially strains belonging to the genera *Beauveria*, *Isaria*, and *Metarhizium*, in producing structurally diverse dihydrochalcone, methylglucosylated, and *O*-demethylated derivatives. These findings provide a useful basis for selecting fungal strains for the targeted synthesis of functionalized chalcone metabolites and support the use of entomopathogenic fungi as sustainable whole-cell biocatalysts.

## Figures and Tables

**Table 1:** Comparative ^13^C NMR data supporting structural changes during cascade biotransformation of 4′-hydroxy-2-methoxychalcone (**1**).

Carbon	1 *	1A *	1B *	1C **
C=O	187.31	197.56	197.96	198.74
α	121.91	37.59	37.79	39.15
β	137.29	24.94	24.84	25.97
1	123.16	128.21	128.88	128.80
2	158.14	157.11	157.11	156.00
3	111.78	110.55	110.56	116.12
4	132.01	129.61	129.59	128.04
5	120.71	120.24	120.23	120.50
6	128.37	127.36	127.38	131.13
1′	129.26	129.01	130.53	130.07
2′,6′	131.12	130.45	130.02	131.40
3′,5′	115.42	115.22	115.85	116.01
4′	162.14	161.95	160.92	162.77
C-2-OCH_3_	55.72	55.25	55.24	-
1″	-	-	99.45	-
2″	-	-	73.34	-
3″	-	-	76.22	-
4″	-	-	78.88	-
5″	-	-	75.66	-
6″	-	-	60.14	-
C-4″-OCH_3_	-	-	59.69	-

* spectrum obtained in DMSO-*d*_6_; ** spectrum obtained in acetone-*d*_6_.

**Table 2:** Comparative ^13^C NMR data supporting structural changes during cascade biotransformation of 4′-hydroxy-3-methoxychalcone.

Carbon	2 *	2A *	2B *	2C **
C=O	187.15	197.27	197.70	197.64
α	122.37	38.88	40.14	40.25
β	142.75	29.83	30.62	30.91
1	136.32	143.07	142.93	144.29
2	113.20	114.09	114.09	116.22
3	159.66	159.28	159.28	158.31
4	116.45	111.28	111.31	113.65
5	129.92	129.26	129.27	130.14
6	121.55	120.64	120.64	120.37
1′	129.10	128.24	129.58	130.17
2′,6′	131.27	130.51	130.08	131.30
3′,5′	115.40	115.23	115.83	116.00
4′	162.25	162.06	160.95	162.71
C-3-OCH_3_	55.31	54.91	54.92	-
1″	-	-	99.41	-
2″	-	-	73.34	-
3″	-	-	76.22	-
4″	-	-	78.88	-
5″	-	-	75.65	-
6″	-	-	60.13	-
C-4″-OCH_3_	-	-	59.72	-

* spectrum obtained in DMSO-*d*_6_; ** spectrum obtained in acetone-*d*_6_.

**Table 3:** Comparative ^13^C NMR data supporting structural changes during cascade biotransformation of 4′-hydroxy-4-methoxychalcone (**3**).

Carbon	3 *	3A ***	3B **	3C **	3D **	3E **	3F **
C=O	187.18	199.46	198.11	188.11	198.18	73.03	197.70
α	119.64	40.50	40.72	120.29	40.89	42.57	40.50
β	142.80	29.78	29.99	142.18	30.13	31.89	30.35
1	127.58	133.40	134.39	128.73	133.15	135.13	135.82
2	130.64	129.47	130.20	131.23	130.20	130.08	130.14
3	115.41	114.10	114.56	115.23	116.84	116.97	117.32
4	161.20	158.03	158.97	162.15	156.49	158.82	157.09
5	115.41	114.10	114.56	115.23	116.84	116.97	117.32
6	130.64	129.47	130.20	131.23	130.20	130.08	130.14
1′	129.41	130.97	132.18	133.35	132.24	140.68	130.22
2′,6′	131.12	130.96	130.84	131.27	130.85	127.73	131.29
3′,5′	114.46	115.63	116.84	116.93	115.99	114.54	116.03
4′	162.07	161.01	162.21	162.62	162.22	157.74	162.73
C-4-OCH_3_	55.43	55.45	55.52	55.79	-	55.40	-
1″	-	-	101.01	101.04	101.04	101.75	101.85
2″	-	-	74.81	74.83	74.84	74.84	74.97
3″	-	-	77.87	77.89	77.91	77.91	78.00
4″	-	-	79.98	80.03	80.00	80.00	80.11
5″	-	-	77.10	77.15	77.14	76.95	76.96
6″	-	-	61.98	62.01	61.99	62.02	62.09
C-4″-OCH_3_	-	-	60.56	60.57	60.56	60.52	60.52

* spectrum obtained in DMSO-*d*_6_; ** spectrum obtained in acetone-*d*_6_; *** spectrum obtained in CDCl_3_.

## Data Availability

Data are contained within the article and Supplementary Materials.

## Supplementary Materials

The following supporting information can be downloaded at: https://www.mdpi.com/article/10.3390/ijms27167463/s1.
