# Supplementary material for: Influence of Methoxy Substitution Pattern on Cascade Biotransformation of 4′-Hydroxychalcones by Entomopathogenic Fungi
Source: Int J Mol Sci. 2026 Aug 20;27(16):7463. doi: 10.3390/ijms27167463 (PMC13513232; doi:10.3390/ijms27167463)
Supplement: Supplementary file 1 [file ijms-27-07463-s001.zip › ijms-4465924-supplementary.pdf]

Supporting Information for

Influence of Methoxy Substitution Pattern on Cascade Biotransformation of 4′-Hydroxychalcones by Entomopathogenic Fungi

Paweł Chlipała[1*], Julia Bienia[1], Tomasz Tronina[1], Jerzy Ł. Wiśniewski[2], and Tomasz Janeczko[1*]

[1]Department of Food Chemistry and Biocatalysis, Faculty of Biotechnology and Food Science, Wrocław University of Environmental and Life Sciences, 50-375 Wrocław, Poland; 125375@student.upwr.edu.pl (J.B.), tomasz.tronina@upwr.edu.pl (T.T.).

[2] Department of Biochemistry, Molecular Biology and Biotechnology, Faculty of Chemistry, Wrocław University of Science and Technology, Łukasiewicza 2, 50-371 Wrocław, Poland, jerzy.wisniewski@pwr.edu.pl (J.Ł.W)

*Correspondence: pawel.chlipala@upwr.edu.pl (P.C.) and tomasz.janeczko@upwr.edu.pl (T.J.)

Contents:

Figure S1. ^{1}H NMR spectra of 4′-*O*-β-D-(4″-*O*-methylglucopyranosyl)-2-methoxydihydrochalcone (**1B**) (DMSO-d_6, 600 MHz)

Figure S2. ^{13}C NMR spectra of 4′-*O*-β-D-(4″-*O*-methylglucopyranosyl)-2-methoxydihydrochalcone (**1B**) (DMSO-d_6, 151 MHz)

Figure S3. COSY spectrum of 4′-*O*-β-D-(4″-*O*-methylglucopyranosyl)-2-methoxydihydrochalcone (**1B**) (DMSO-d_6, 600 MHz)

Figure S4. HSQC spectrum of 4′-*O*-β-D-(4″-*O*-methylglucopyranosyl)-2-methoxydihydrochalcone (**1B**) (DMSO-d_6, 600/151 MHz)

Figure S5. HMBC spectrum of 4′-*O*-β-D-(4″-*O*-methylglucopyranosyl)-2-methoxydihydrochalcone (**1B**) (DMSO-d_6, 600/151 MHz)

Figure S6. ^{1}H NMR spectra of 2,4′-dihydroxydihydrochalcone (**1C**) (Acetone-d_6, 600 MHz)

Figure S7. ^{13}C NMR spectra of 2,4′-dihydroxydihydrochalcone (**1C**) (Acetone-d_6, 151 MHz)

Figure S8. COSY spectrum of 2,4′-dihydroxydihydrochalcone (**1C**) (Acetone-d_6, 600 MHz)

Figure S9. HSQC spectrum of 2,4′-dihydroxydihydrochalcone (**1C**) (Acetone-d_6, 600/151 MHz)

Figure S10. HMBC spectrum of 2,4′-dihydroxydihydrochalcone (**1C**) (Acetone-d_6, 600/151 MHz)

Figure S11. ^{1}H NMR spectra of 4′-*O*-β-D-(4″-*O*-methylglucopyranosyl)-3-methoxydihydrochalcone (**2B**) (DMSO-d_6, 600 MHz)

Figure S12. ^{13}C NMR spectra of 4′-*O*-β-D-(4″-*O*-methylglucopyranosyl)-3-methoxydihydrochalcone (**2B**) (DMSO-d_6, 151 MHz)

Figure S13. COSY spectrum of 4′-*O*-β-D-(4″-*O*-methylglucopyranosyl)-3-methoxydihydrochalcone (**2B**) (DMSO-d_6, 600 MHz)

Figure S14. HSQC spectrum of 4′-*O*-β-D-(4″-*O*-methylglucopyranosyl)-3-methoxydihydrochalcone (**2B**) (DMSO-d_6, 600/151 MHz)

Figure S15. HMBC spectrum of 4′-*O*-β-D-(4″-*O*-methylglucopyranosyl)-3-methoxydihydrochalcone (**2B**) (DMSO-d_6, 600/151 MHz)

Figure S16. ^{1}H NMR spectra of 3,4′-dihydroxydihydrochalcone (**2C**) (Acetone-d_6, 600 MHz)

Figure S17. ^{13}C NMR spectra of 3,4′-dihydroxydihydrochalcone (**2C**) (Acetone-d_6, 151 MHz)

Figure S18. COSY spectrum of 3,4′-dihydroxydihydrochalcone (**2C**) (Acetone-d_6, 600 MHz)

Figure S19. HSQC spectrum of 3,4′-dihydroxydihydrochalcone (**2C**) (Acetone-d_6, 600/151 MHz)

Figure S20. HMBC spectrum of 3,4′-dihydroxydihydrochalcone (**2C**) (Acetone-d_6, 600/151 MHz)

Figure S21. ^{1}H NMR spectra of 4′-*O*-β-D-(4″-*O*-methylglucopyranosyl)-4-methoxydihydrochalcone (**3B**) and 4′-*O*-β-D-(4″-*O*-methylglucopyranosyl)-4-methoxychalcone (**3C**) (Acetone-d_6, 600 MHz)

Figure S22. ^{13}C NMR spectra of 4′-*O*-β-D-(4″-*O*-methylglucopyranosyl)-4-methoxydihydrochalcone (**3B**) and 4′-*O*-β-D-(4″-*O*-methylglucopyranosyl)-4-methoxychalcone (**3C**) (Acetone-d_6, 151 MHz)

Figure S23. COSY spectrum of 4′-*O*-β-D-(4″-*O*-methylglucopyranosyl)-4-methoxydihydrochalcone (**3B**) and 4′-*O*-β-D-(4″-*O*-methylglucopyranosyl)-4-methoxychalcone (**3C**) (Acetone-d_6, 600 MHz)

Figure S24. HSQC spectrum of 4′-*O*-β-D-(4″-*O*-methylglucopyranosyl)-4-methoxydihydrochalcone (**3B**) and 4′-*O*-β-D-(4″-*O*-methylglucopyranosyl)-4-methoxychalcone (**3C**) (Acetone-d_6, 600/151 MHz)

Figure S25. HMBC spectrum of 4′-*O*-β-D-(4″-*O*-methylglucopyranosyl)-4-methoxydihydrochalcone (**3B**) and 4′-*O*-β-D-(4″-*O*-methylglucopyranosyl)-4-methoxychalcone (**3C**) (Acetone-d_6, 600/151 MHz)

Figure S26. ^{1}H NMR spectra of 4′-*O*-β-D-(4″-*O*-methylglucopyranosyl)-4-hydroxydihydrochalcone (**3D**), 4′-*O*-β-D-(4″-*O*-methylglucopyranosyl)-4-methoxydihydrochalc-1-ol (**3E**) and 4-*O*-β-D-(4″-*O*-methylglucopyranosyl)-4′-hydroxydihydrochalcone (**3F**) (Acetone-d_6, 600 MHz)

Figure S27. ^{13}C NMR spectra of 4′-*O*-β-D-(4″-*O*-methylglucopyranosyl)-4-hydroxydihydrochalcone (**3D**), 4′-*O*-β-D-(4″-*O*-methylglucopyranosyl)-4-methoxydihydrochalc-1-ol (**3E**) and 4-*O*-β-D-(4″-*O*-methylglucopyranosyl)-4′-hydroxydihydrochalcone (**3F**) (Acetone-d_6, 151 MHz)

Figure S28. COSY spectrum of 4′-*O*-β-D-(4″-*O*-methylglucopyranosyl)-4-hydroxydihydrochalcone (**3D**), 4′-*O*-β-D-(4″-*O*-methylglucopyranosyl)-4-methoxydihydrochalc-1-ol (**3E**) and 4-*O*-β-D-(4″-*O*-methylglucopyranosyl)-4′-hydroxydihydrochalcone (**3F**) (Acetone-d_6, 600 MHz)

Figure S29. HSQC spectrum of 4′-*O*-β-D-(4″-*O*-methylglucopyranosyl)-4-hydroxydihydrochalcone (**3D**), 4′-*O*-β-D-(4″-*O*-methylglucopyranosyl)-4-methoxydihydrochalc-1-ol (**3E**) and 4-*O*-β-D-(4″-*O*-methylglucopyranosyl)-4′-hydroxydihydrochalcone (**3F**) (Acetone-d_6, 600/151 MHz)

Figure S30. HMBC spectrum of 4′-*O*-β-D-(4″-*O*-methylglucopyranosyl)-4-hydroxydihydrochalcone (**3D**), 4′-*O*-β-D-(4″-*O*-methylglucopyranosyl)-4-methoxydihydrochalc-1-ol (**3E**) and 4-*O*-β-D-(4″-*O*-methylglucopyranosyl)-4′-hydroxydihydrochalcone (**3F**) (Acetone-d_6, 600/151 MHz)

Table S1. High-resolution ESI-QTOF-MS data for isolated or enriched biotransformation products.

Figure S31. HR-ESI-QTOF-MS spectrum of 4′-hydroxy-2-methoxydihydrochalcone (**1A**).

Figure S32. HR-ESI-QTOF-MS spectrum of 4′-*O*-β-D-(4″-*O*-methylglucopyranosyl)-2-methoxydihydrochalcone (**1B**).

Figure S33. HR-ESI-QTOF-MS spectrum of 4′-hydroxy-3-methoxydihydrochalcone (**2A**).

Figure S34. HR-ESI-QTOF-MS spectrum of 4′-*O*-β-D-(4″-*O*-methylglucopyranosyl)-3-methoxydihydrochalcone (**2B**).

Figure S35. HR-ESI-QTOF-MS spectrum of 3,4′-dihydroxydihydrochalcone (**2C**).

Figure S36. HR-ESI-QTOF-MS spectrum of 4′-hydroxy-4-methoxydihydrochalcone (**3A**).

Figure S37. HR-ESI-QTOF-MS spectra of the enriched fraction containing compounds **3B** and **3C**.

Figure S38. HR-ESI-QTOF-MS spectra supporting the elemental composition assigned to the isomeric compounds **3D** and **3F** in the enriched fraction.

Figure S39. HR-ESI-QTOF-MS analysis supporting the elemental composition assigned to compound **3E** in the enriched fraction.

Table S2. Time-resolved UPLC-DAD profiles of the biotransformation of compounds **1–3** by entomopathogenic filamentous fungi.

Figure S40. Screening of biotransformation of 4′-hydroxy-2-methoxychalcone (**1**) in time (1D–10D).

Figure S41. 3D chromatogram of biotransformation of 4′-hydroxy-2-methoxychalcone (**1**) on day 1.

Figure S42. Screening of biotransformation of 4′-hydroxy-3-methoxychalcone (**2**) in time (1D–10D).

Figure S43. 3D chromatogram of biotransformation of 4′-hydroxy-3-methoxychalcone (**2**) on day 1.

Figure S44. Screening of biotransformation of 4′-hydroxy-4-methoxychalcone (**3**) in time (1D–10D).

Figure S45. 3D chromatogram of biotransformation of 4′-hydroxy-4-methoxychalcone (**3**) on day 1.

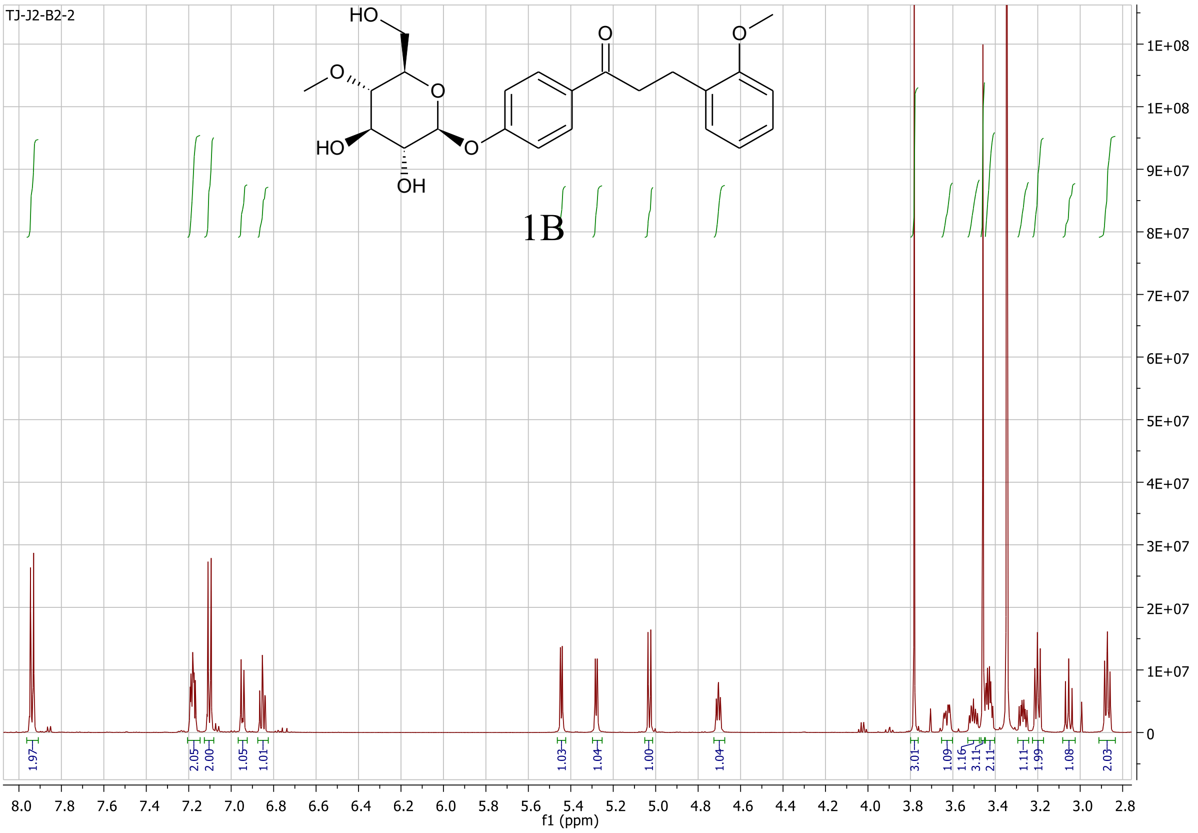

Figure S1. ¹H NMR spectra of 4′-*O*-β-D-(4″-*O*-methylglucopyranosyl)-2-methoxydihydrochalcone (**1B**) (DMSO-*d*₆, 600 MHz)

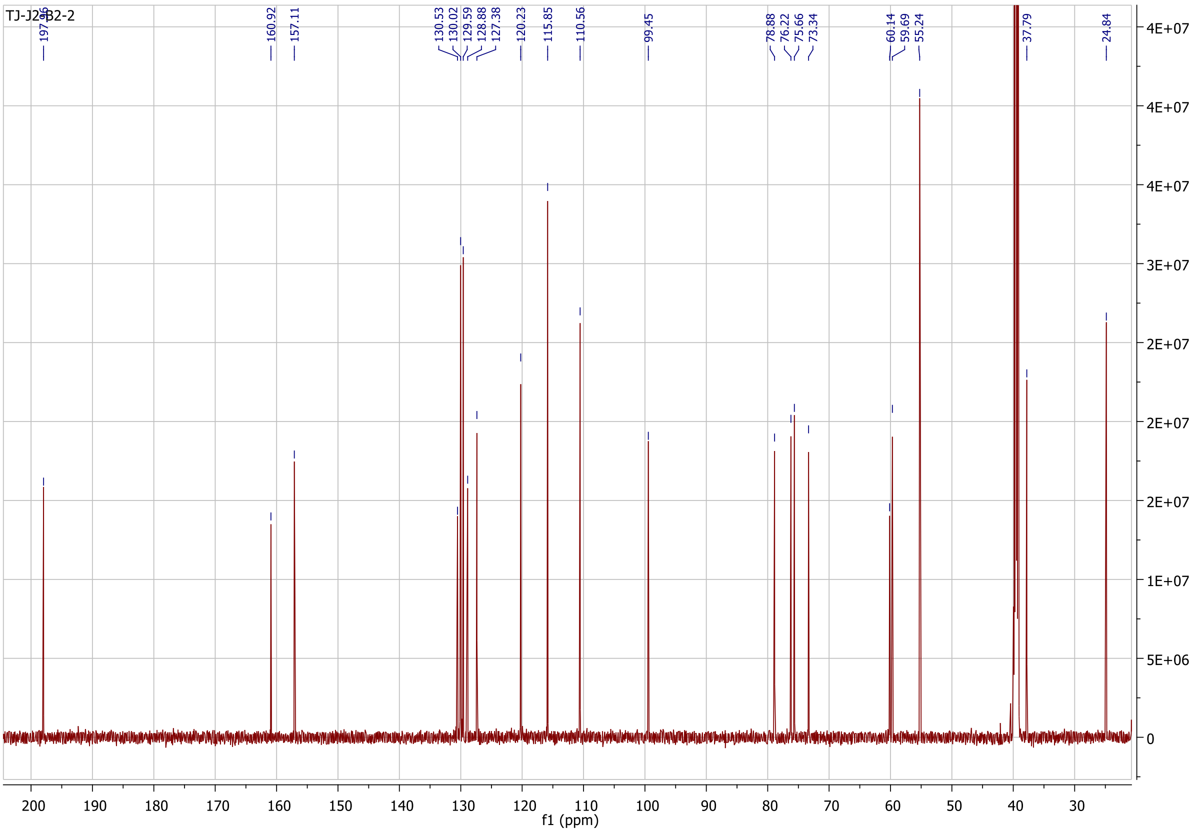

Figure S2. ¹³C NMR spectra of 4′-*O*-β-D-(4″-*O*-methylglucopyranosyl)-2-methoxydihydrochalcone (**1B**) (DMSO-*d*₆, 151 MHz)

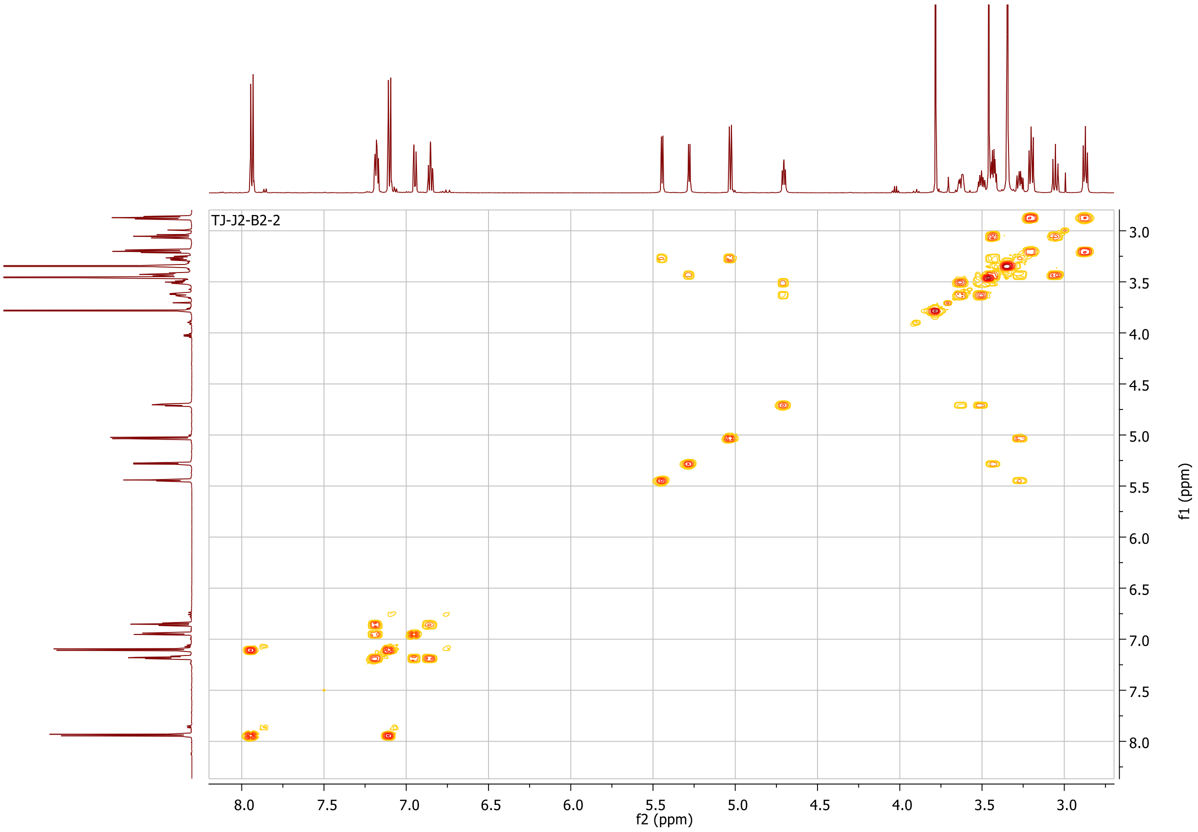

Figure S3. COSY spectrum of 4′-*O*-β-D-(4″-*O*-methylglucopyranosyl)-2-methoxydihydrochalcone (**1B**) (DMSO-d_6, 600 MHz)

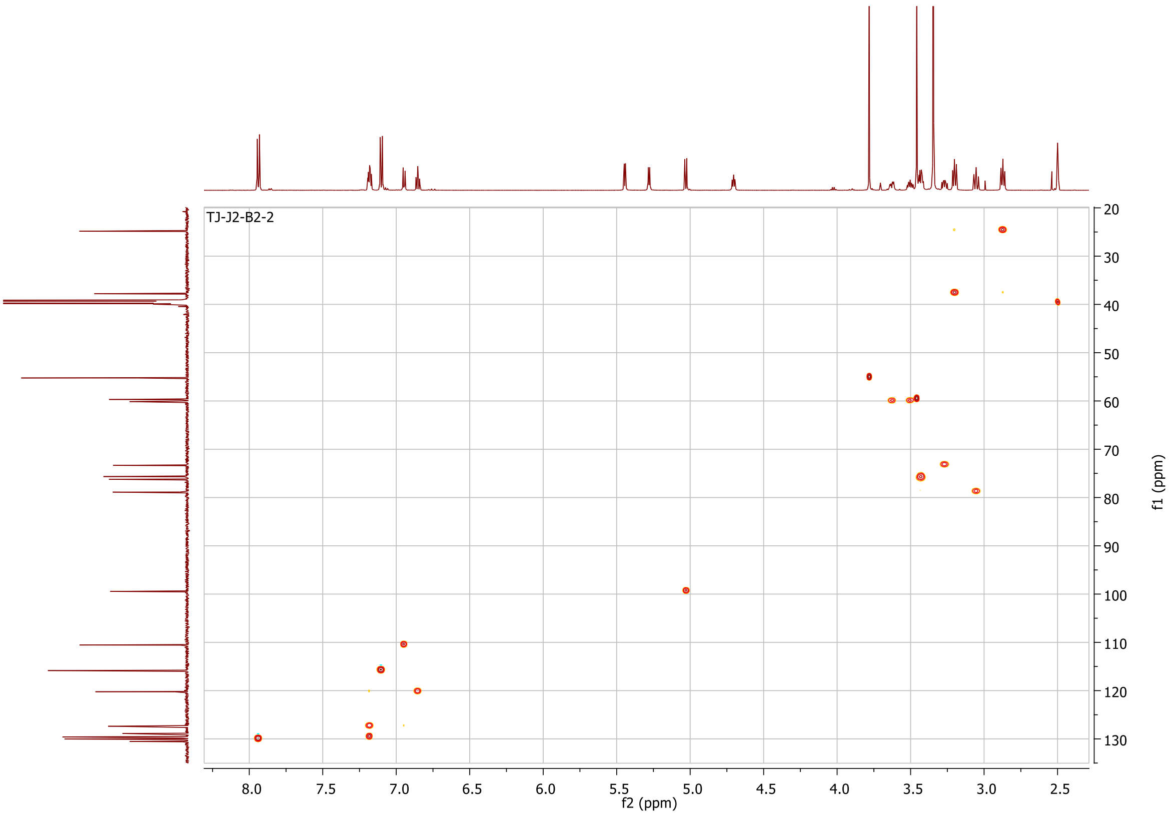

Figure S4. HSQC spectrum of 4′-*O*-β-D-(4″-*O*-methylglucopyranosyl)-2-methoxydihydrochalcone (**1B**) (DMSO-d_6, 600/151 MHz)

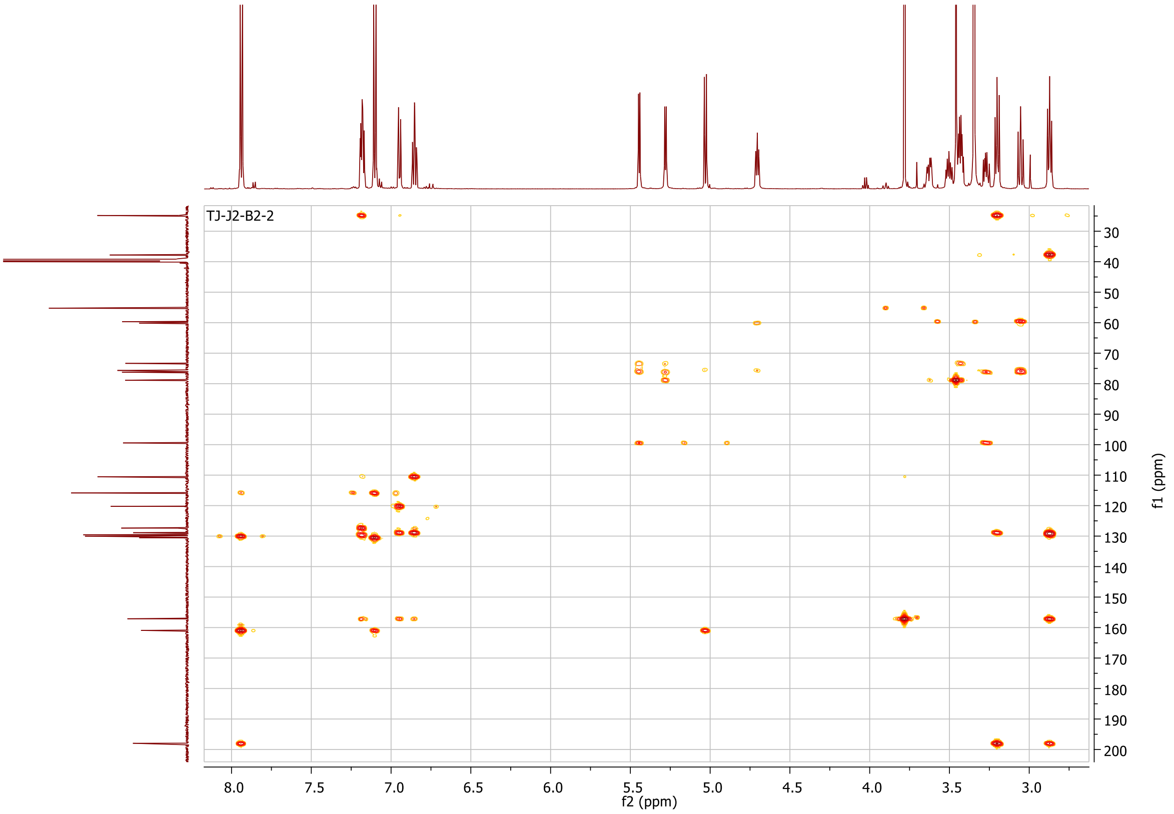

Figure S5. HMBC spectrum of 4′-*O*-β-D-(4″-*O*-methylglucopyranosyl)-2-methoxydihydrochalcone (**1B**) (DMSO-d_6, 600/151 MHz)

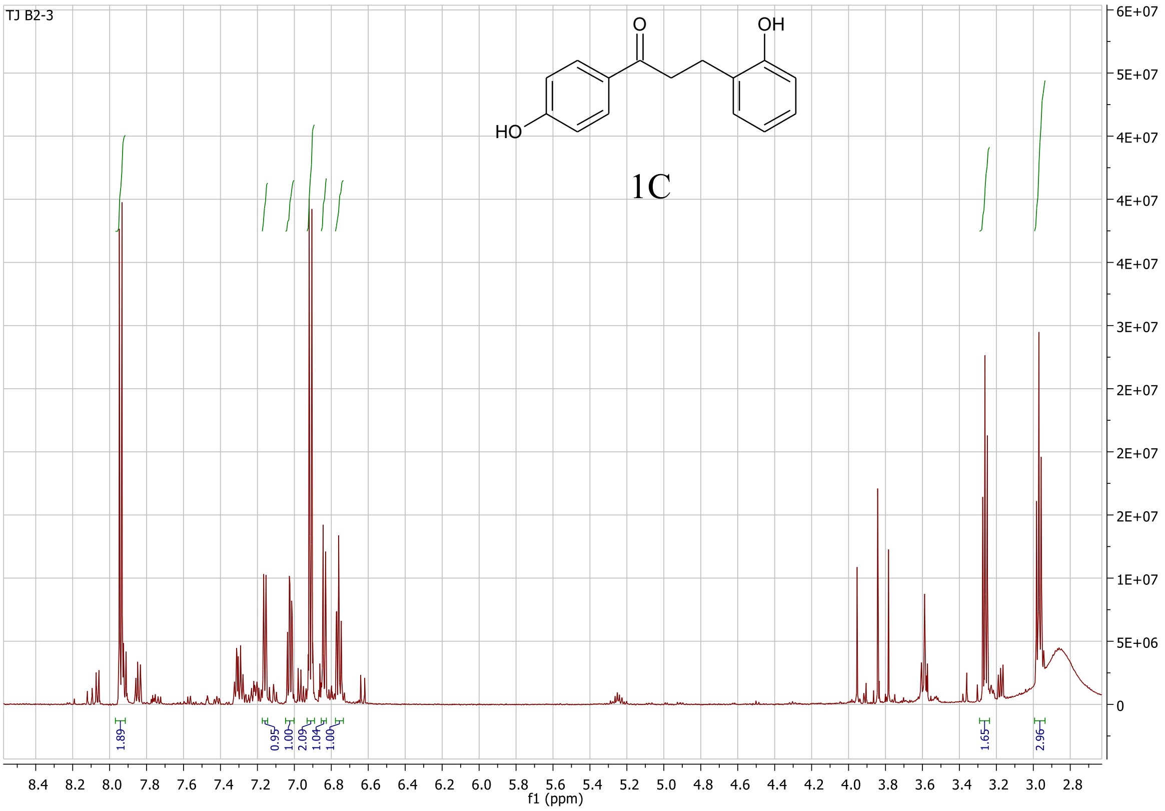

Figure S6. ^{1}H NMR spectra of 2,4′-dihydroxydihydrochalcone (**1C**) (Acetone-d_6, 600 MHz)

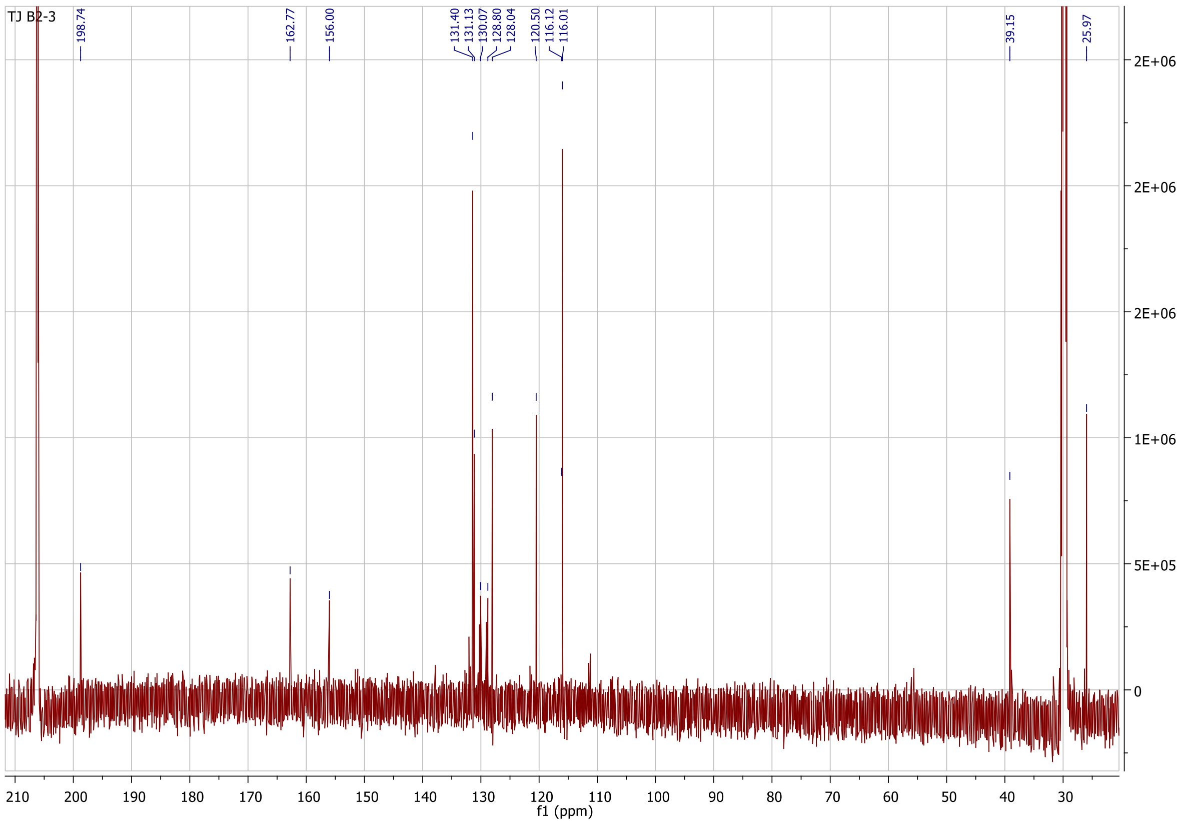

Figure S7. ^{13}C NMR spectra of 2,4′-dihydroxydihydrochalcone (**1C**) (Acetone-*d*$_6$, 151 MHz)

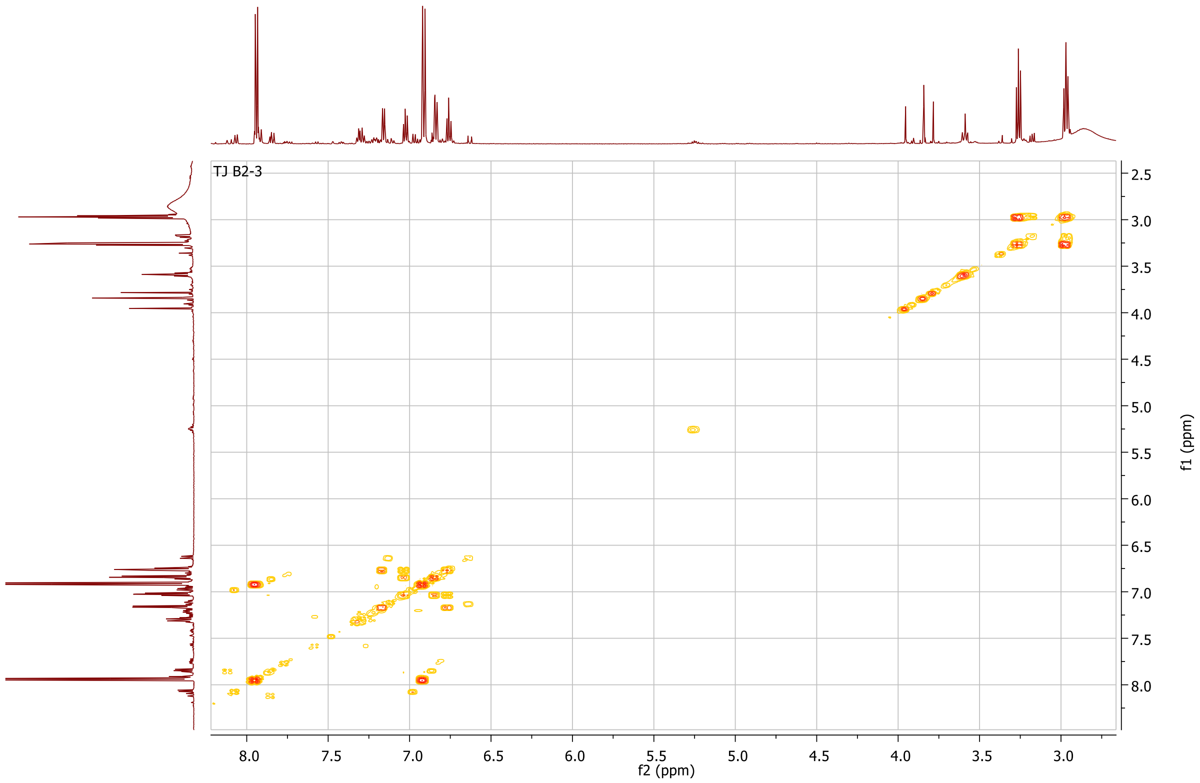

Figure S8. COSY spectrum of 2,4′-dihydroxydihydrochalcone (**1C**) (Acetone-*d*$_6$, 600 MHz)

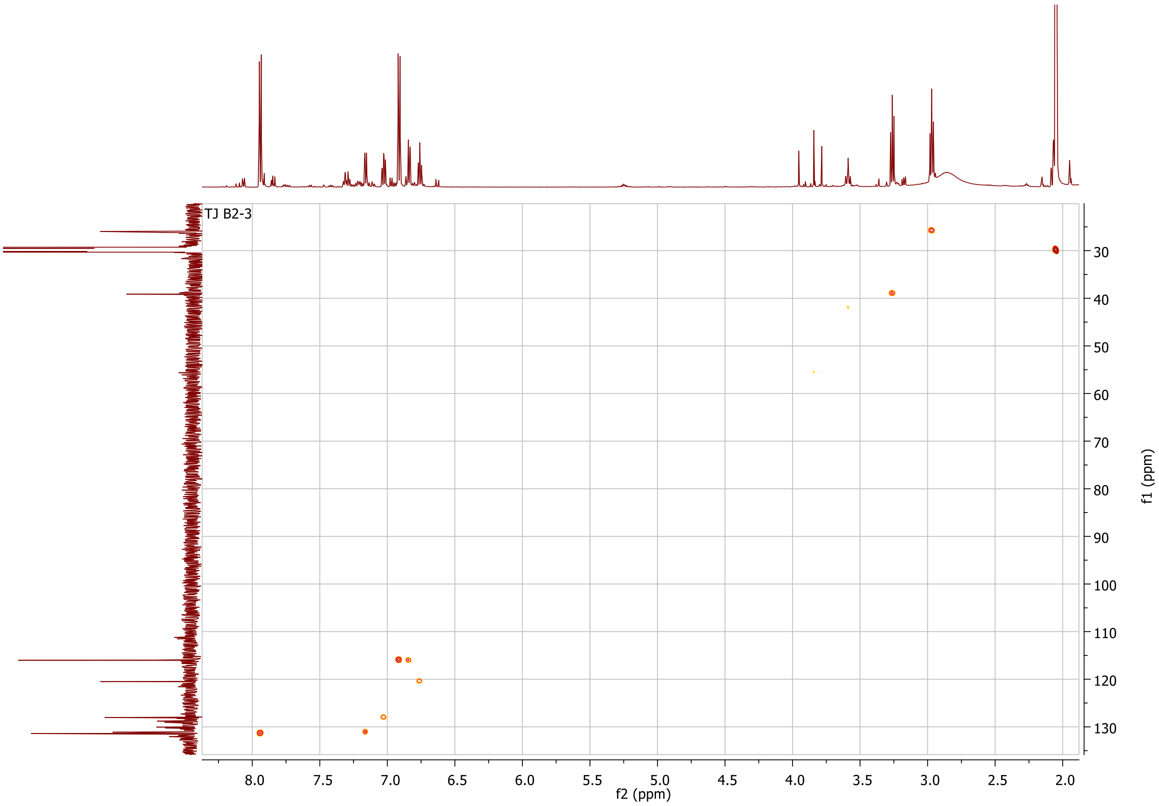

Figure S9. HSQC spectrum of 2,4′-dihydroxydihydrochalcone (**1C**) (Acetone-d_6, 600/151 MHz)

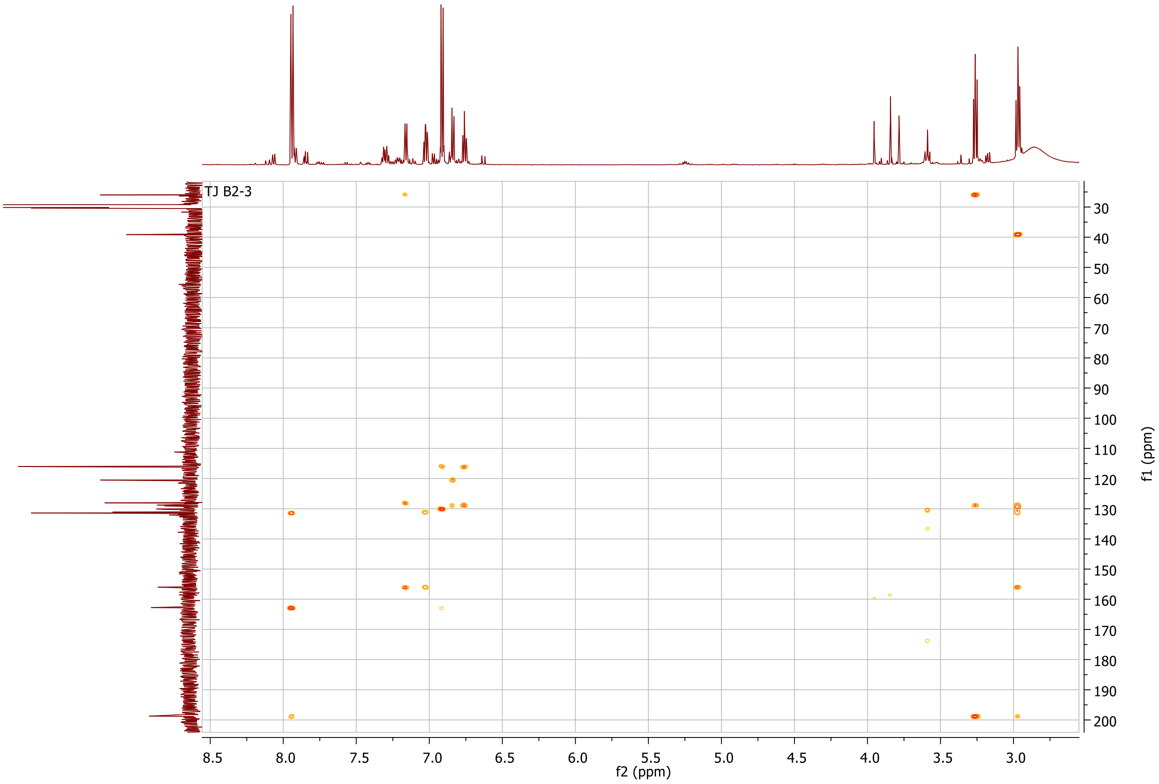

Figure S10. HMBC spectrum of 2,4′-dihydroxydihydrochalcone (**1C**) (Acetone-d_6, 600/151 MHz)

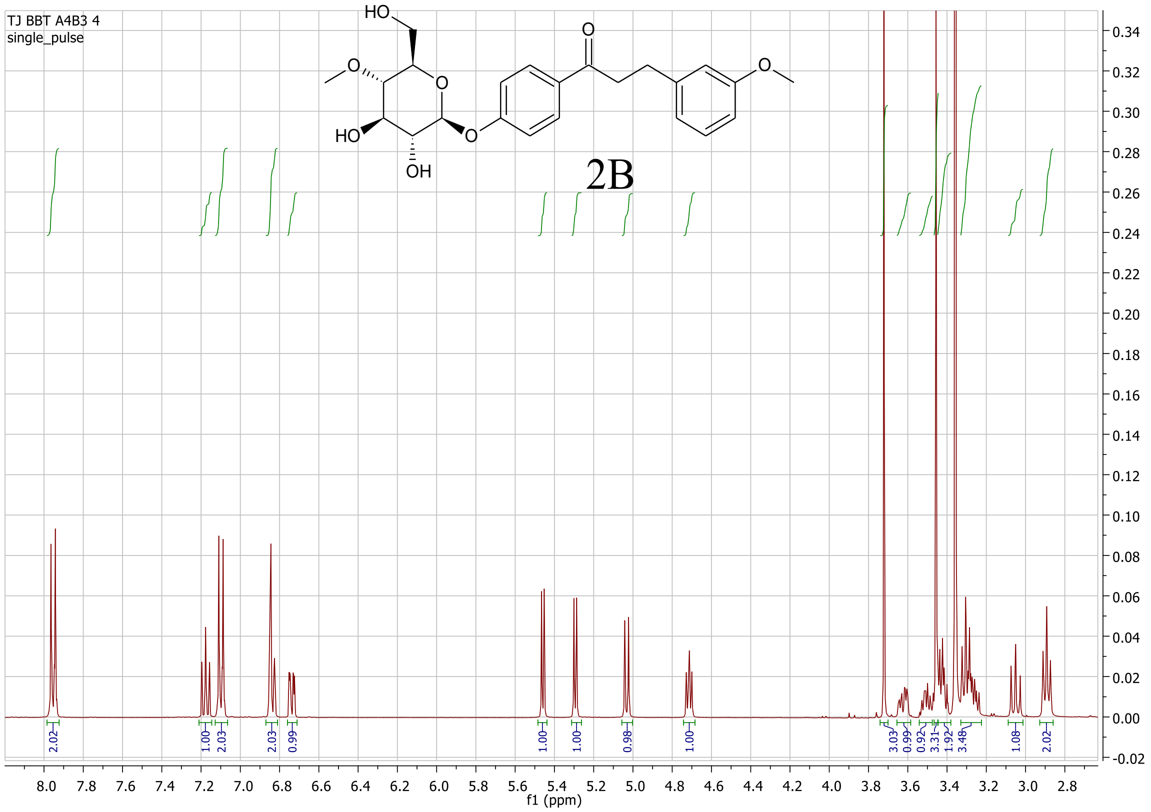

Figure S11. ^{1}H NMR spectra of 4′-*O*-β-D-(4″-*O*-methylglucopyranosyl)-3-methoxydihydrochalcone (**2B**) (DMSO-*d*$_6$, 600 MHz)

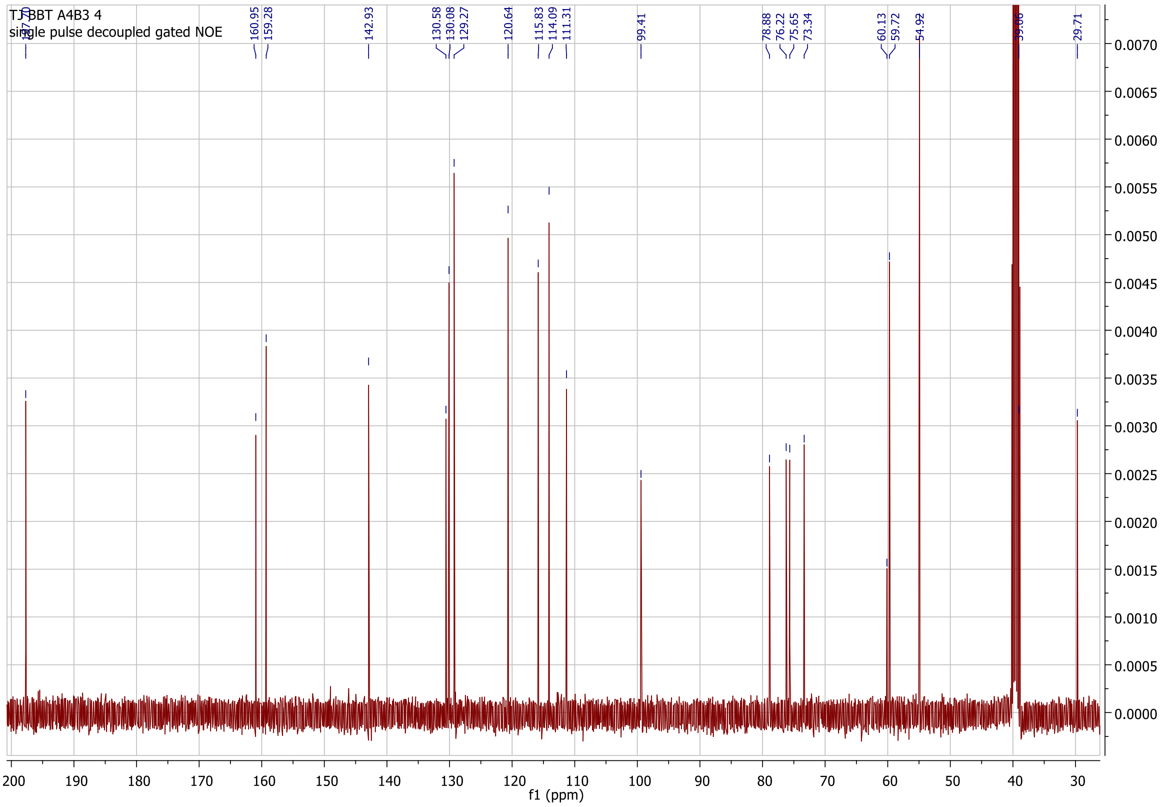

Figure S12. ^{13}C NMR spectra of 4′-*O*-β-D-(4″-*O*-methylglucopyranosyl)-3-methoxydihydrochalcone (**2B**) (DMSO-*d*$_6$, 151 MHz)

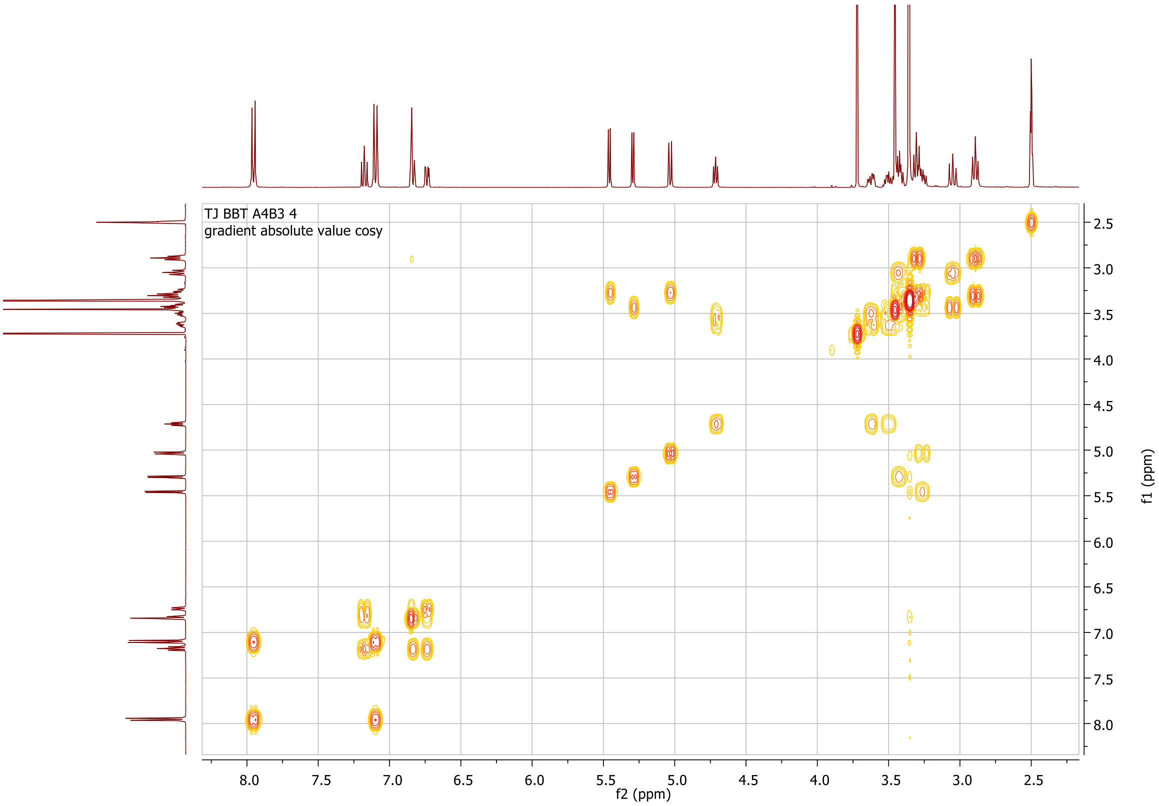

Figure S13. COSY spectrum of 4′-*O*-β-D-(4″-*O*-methylglucopyranosyl)-3-methoxydihydrochalcone (**2B**) (DMSO-*d*$_6$, 600 MHz)

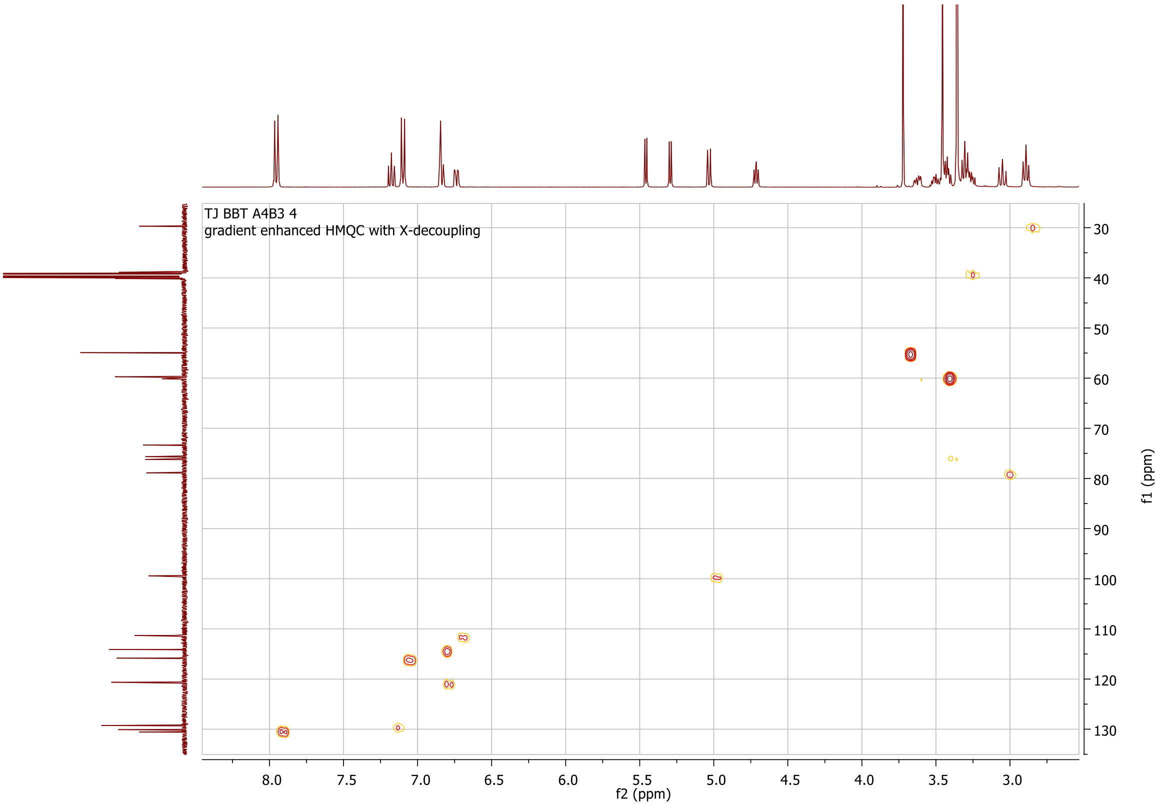

Figure S14. HSQC spectrum of 4′-*O*-β-D-(4″-*O*-methylglucopyranosyl)-3-methoxydihydrochalcone (**2B**) (DMSO-*d*$_6$, 600/151 MHz)

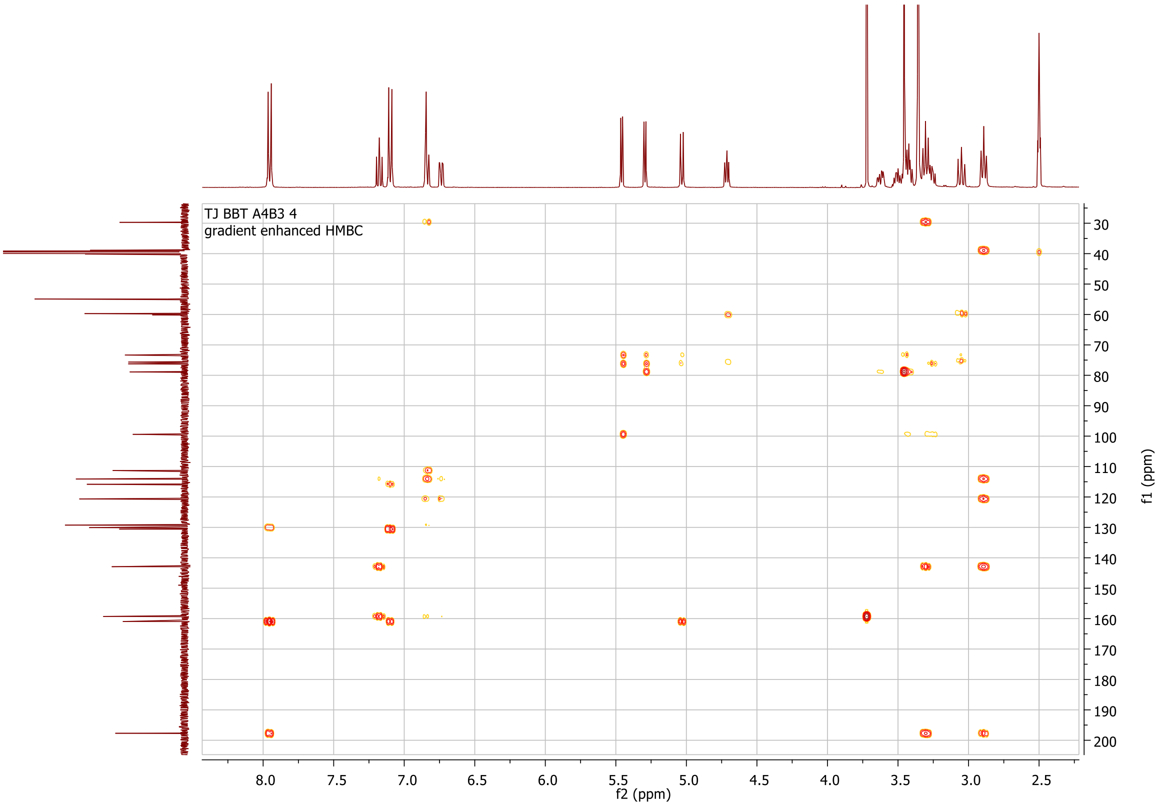

Figure S15. HMBC spectrum of 4′-*O*-β-D-(4″-*O*-methylglucopyranosyl)-3-methoxydihydrochalcone (**2B**) (DMSO-*d*6, 600/151 MHz)

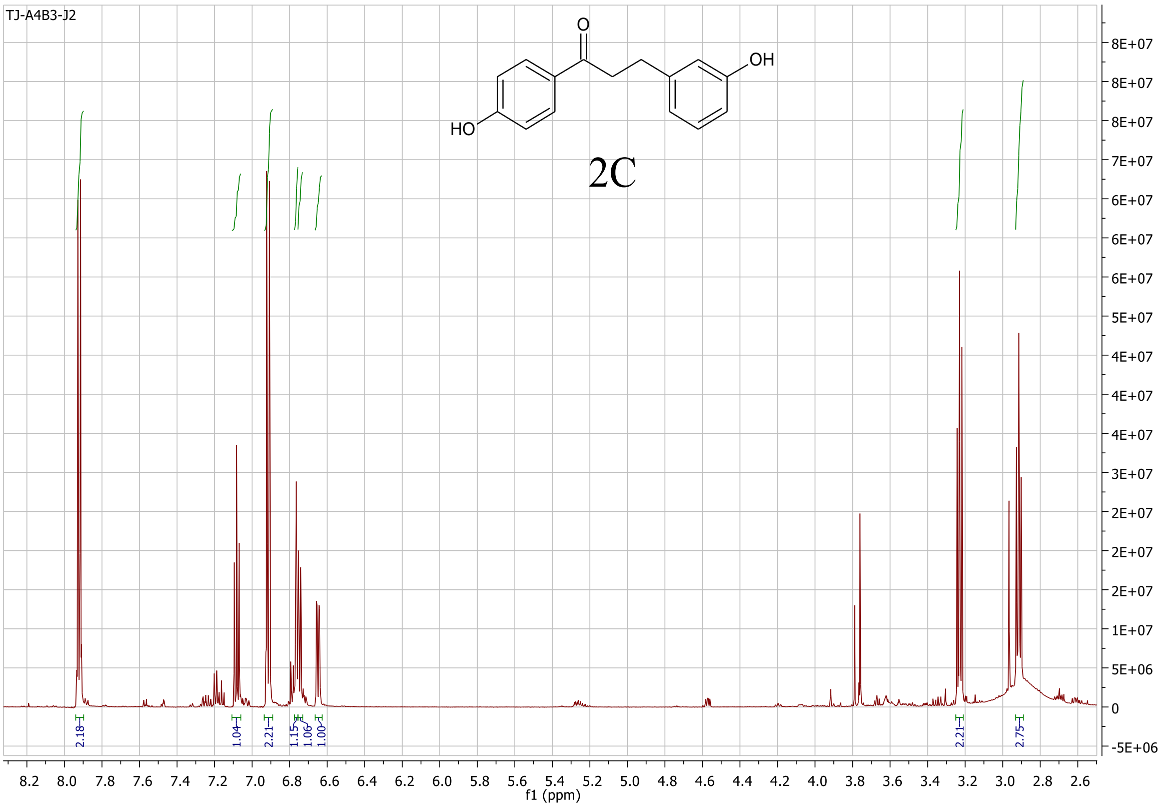

Figure S16. ^{1}H NMR spectra of 3,4′-dihydroxydihydrochalcone (**2C**) (Acetone-*d*6, 600 MHz)

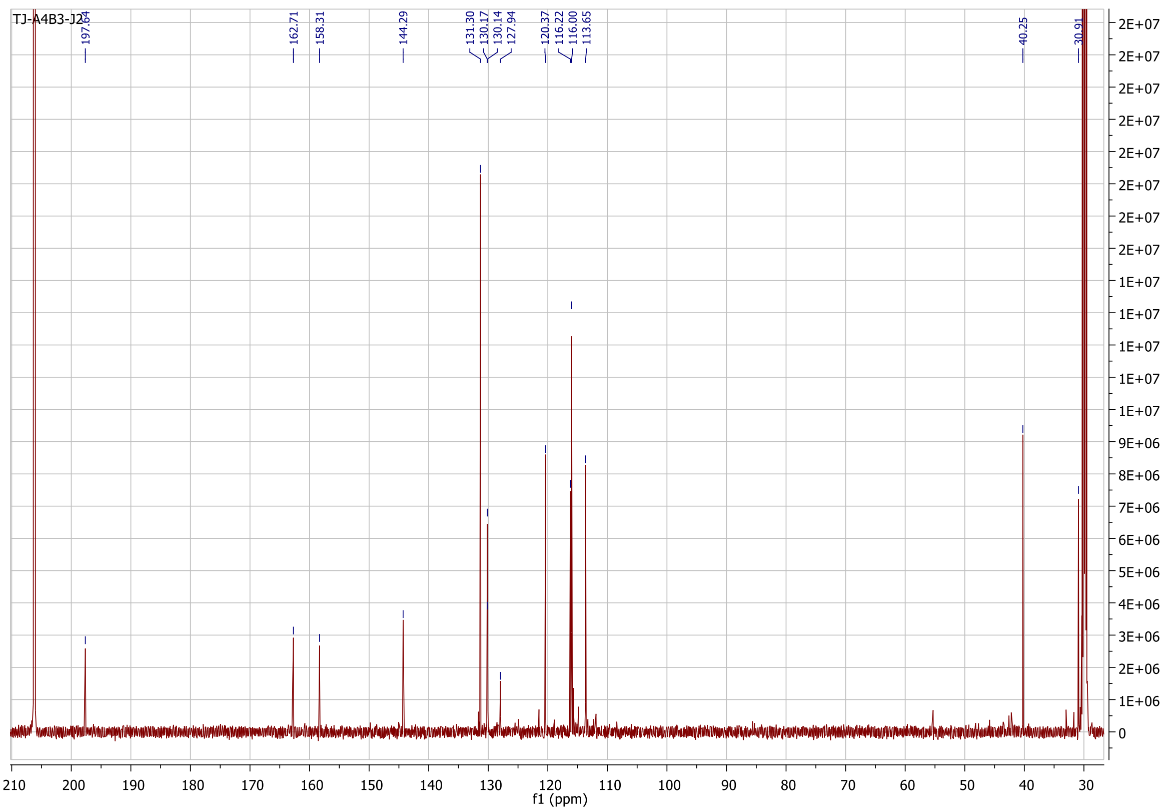

Figure S17. ^{13}C NMR spectra of 3,4′-dihydroxydihydrochalcone (**2C**) (Acetone-d_6, 151 MHz)

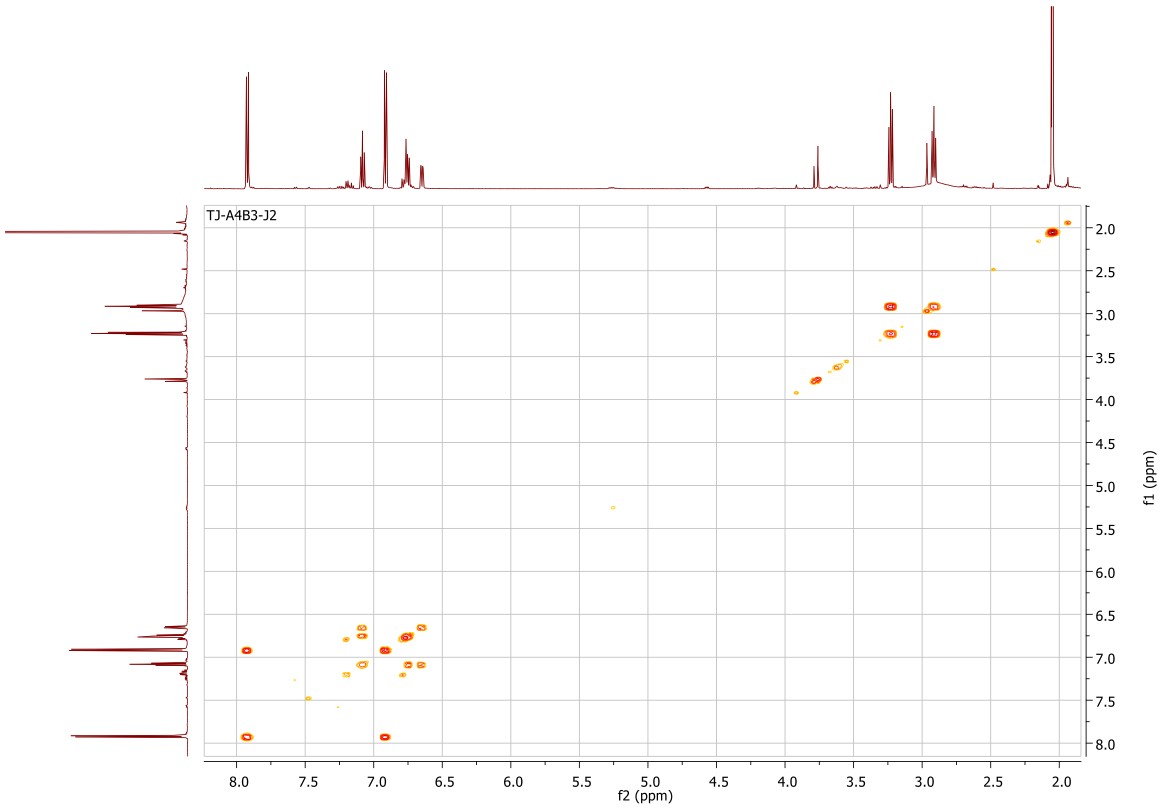

Figure S18. COSY spectrum of 3,4′-dihydroxydihydrochalcone (**2C**) (Acetone-d_6, 600 MHz)

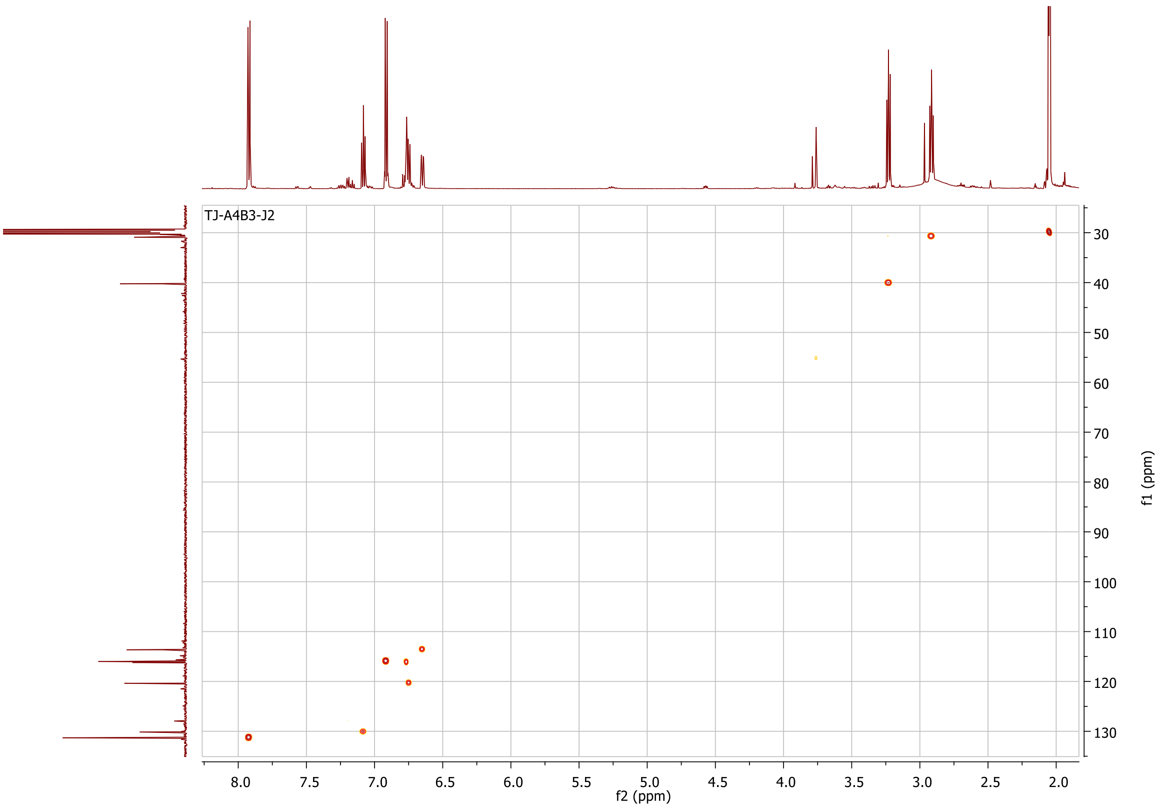

Figure S19. HSQC spectrum of 3,4′-dihydroxydihydrochalcone (**2C**) (Acetone-d_6, 600/151 MHz)

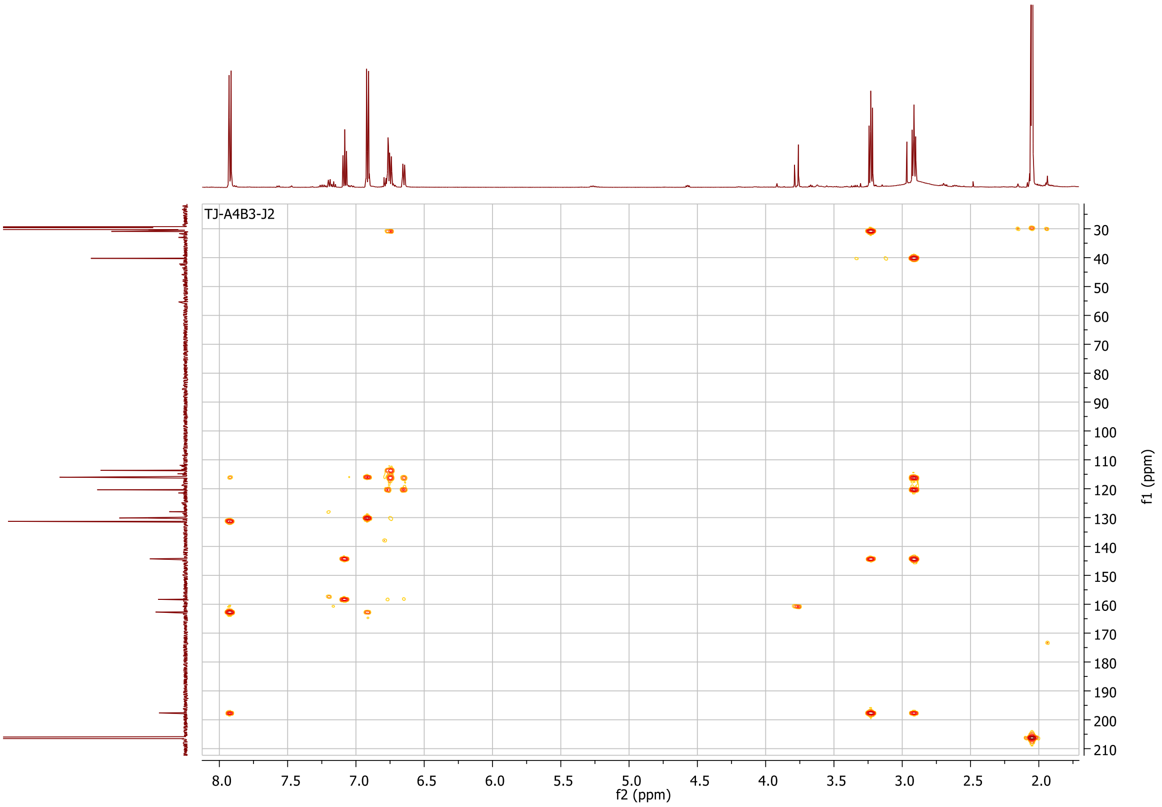

Figure S20. HMBC spectrum of 3,4′-dihydroxydihydrochalcone (**2C**) (Acetone-d_6, 600/151 MHz)

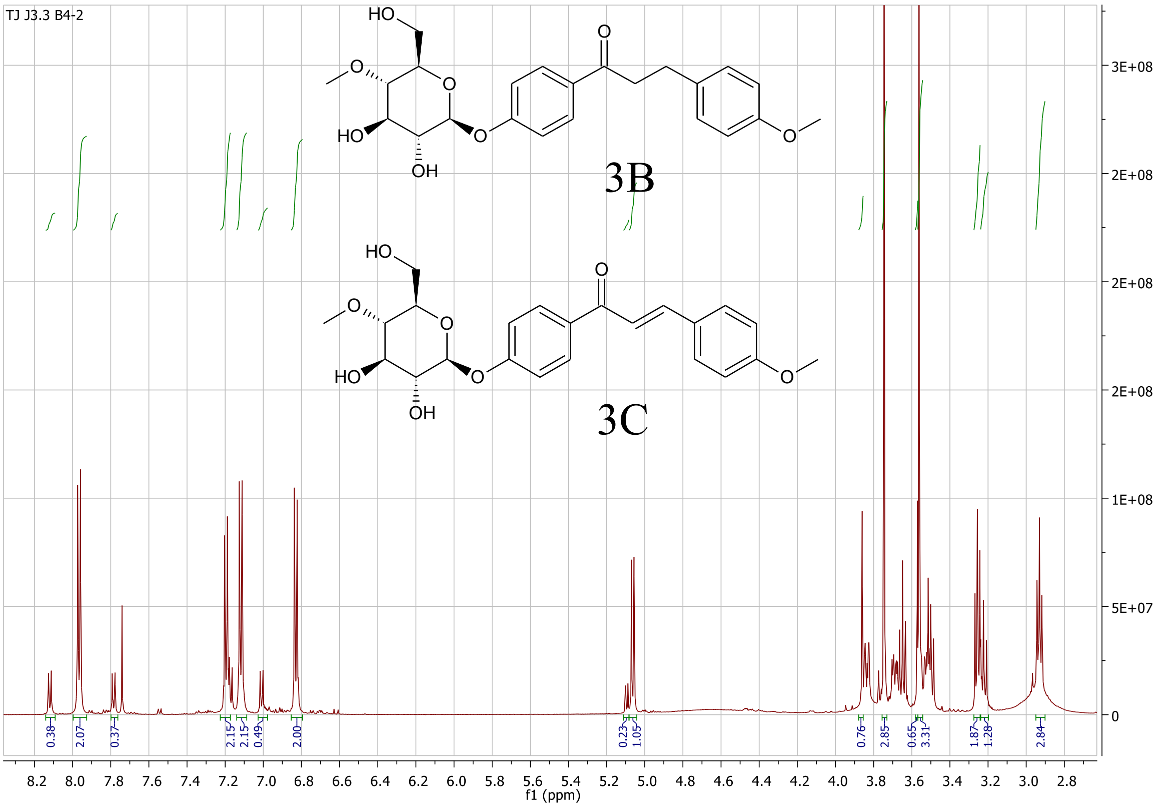

Figure S21. ^{1}H NMR spectra of 4′-*O*-β-D-(4″-*O*-methylglucopyranosyl)-4-methoxydihydrochalcone (**3B**) and 4′-*O*-β-D-(4″-*O*-methylglucopyranosyl)-4-methoxychalcone (**3C**) (Acetone-d_6, 600 MHz)

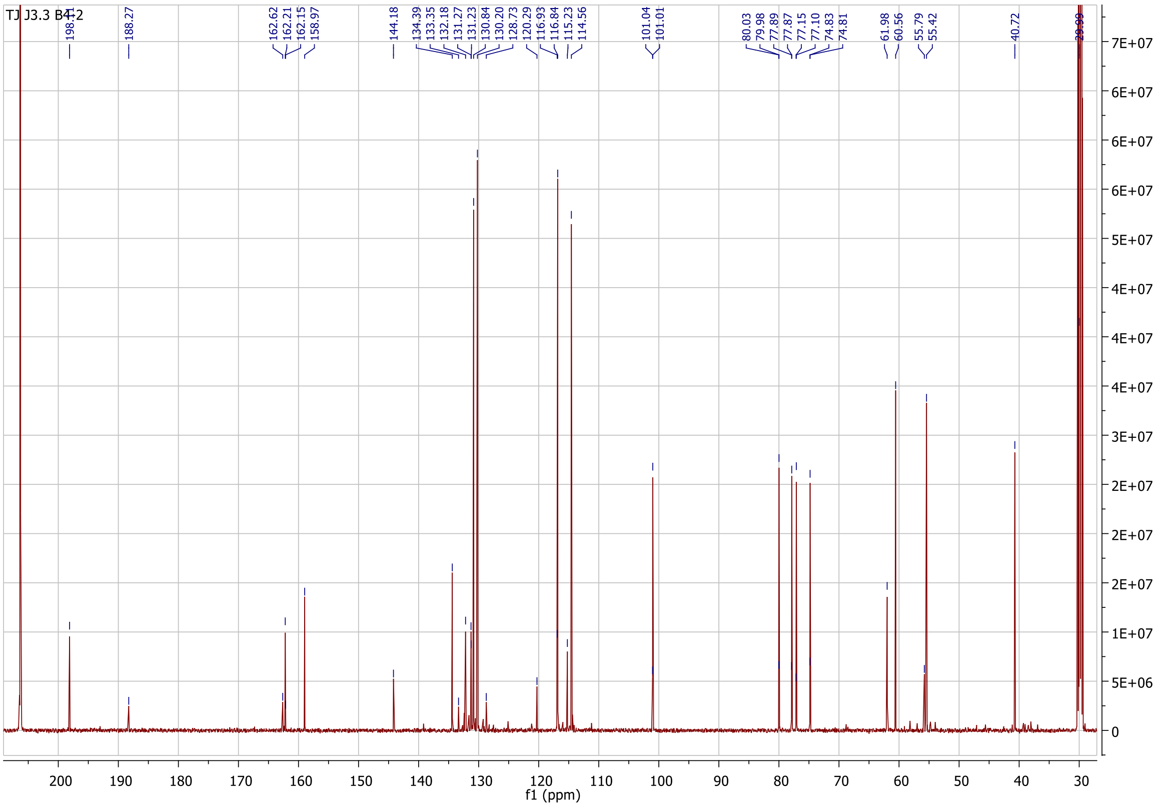

Figure S22. ^{13}C NMR spectra of 4′-*O*-β-D-(4″-*O*-methylglucopyranosyl)-4-methoxydihydrochalcone (**3B**) and 4′-*O*-β-D-(4″-*O*-methylglucopyranosyl)-4-methoxychalcone (**3C**) (Acetone-d_6, 151 MHz)

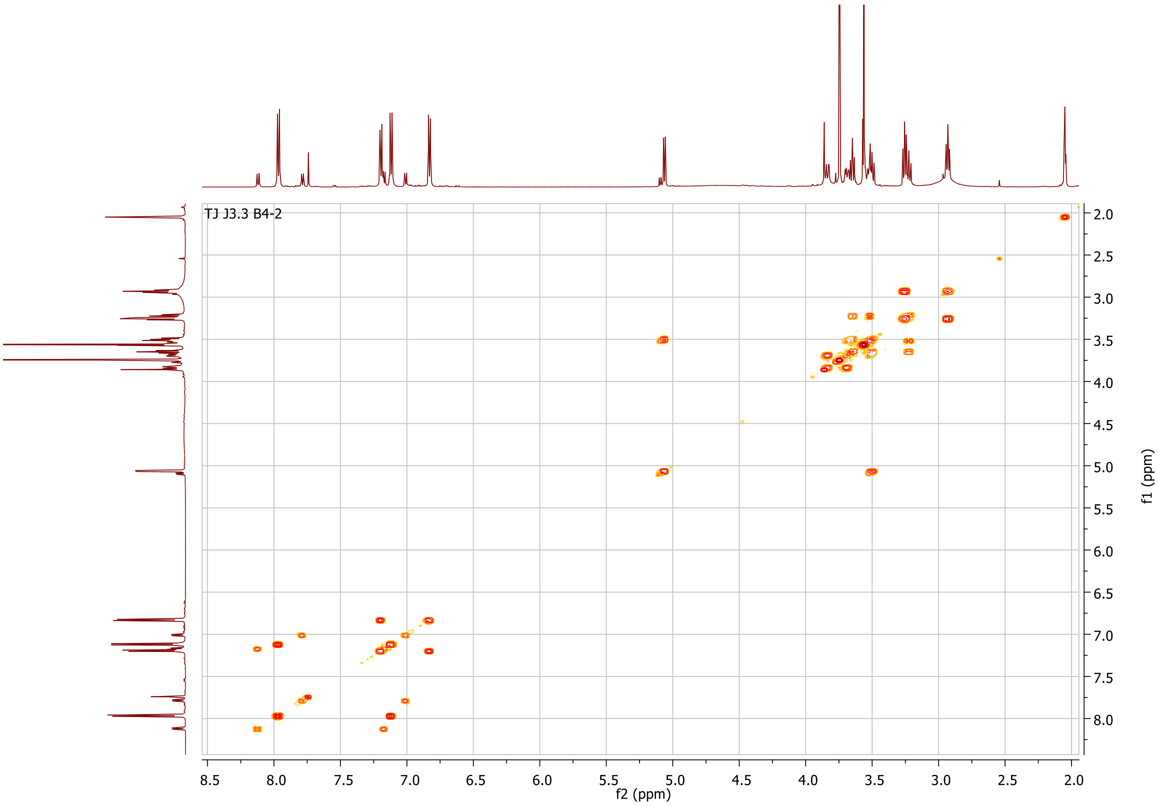

Figure S23. COSY spectrum of 4′-*O*-β-D-(4″-*O*-methylglucopyranosyl)-4-methoxydihydrochalcone (**3B**) and 4′-*O*-β-D-(4″-*O*-methylglucopyranosyl)-4-methoxychalcone (**3C**) (Acetone-d_6, 600 MHz)

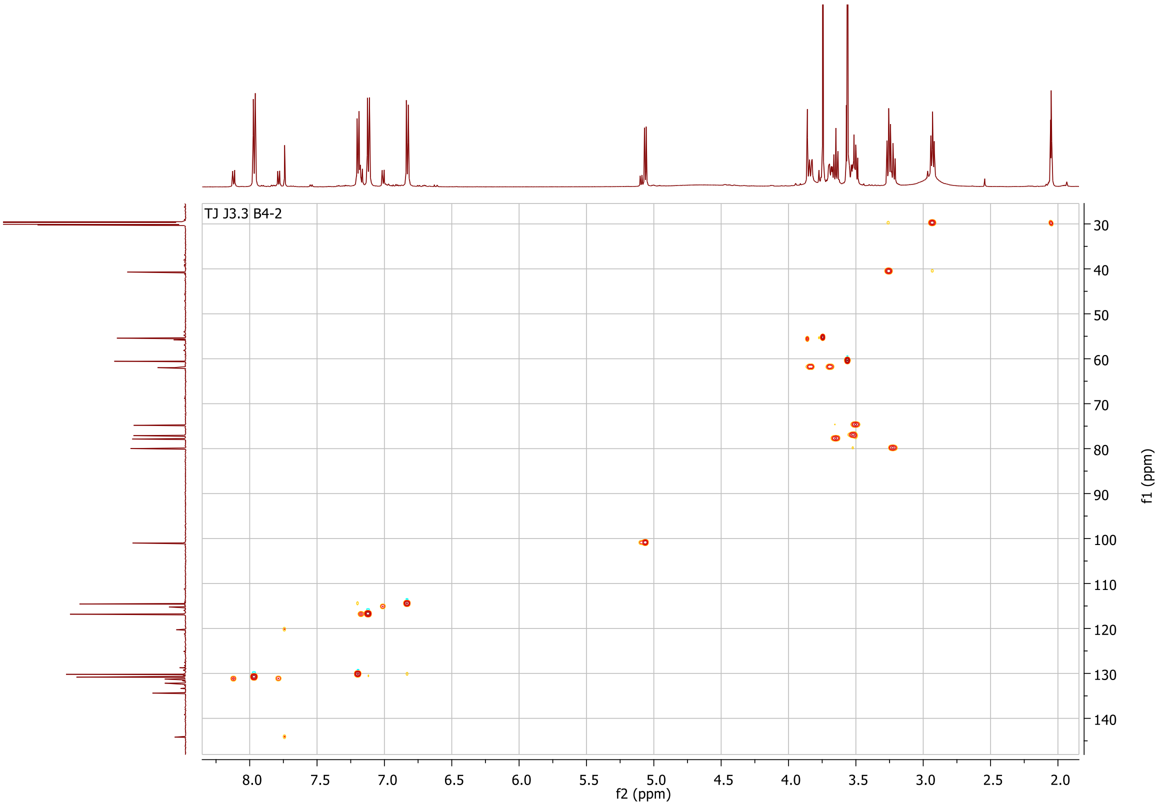

Figure S24. HSQC spectrum of 4′-*O*-β-D-(4″-*O*-methylglucopyranosyl)-4-methoxydihydrochalcone (**3B**) and 4′-*O*-β-D-(4″-*O*-methylglucopyranosyl)-4-methoxychalcone (**3C**) (Acetone-d_6, 600/151 MHz)

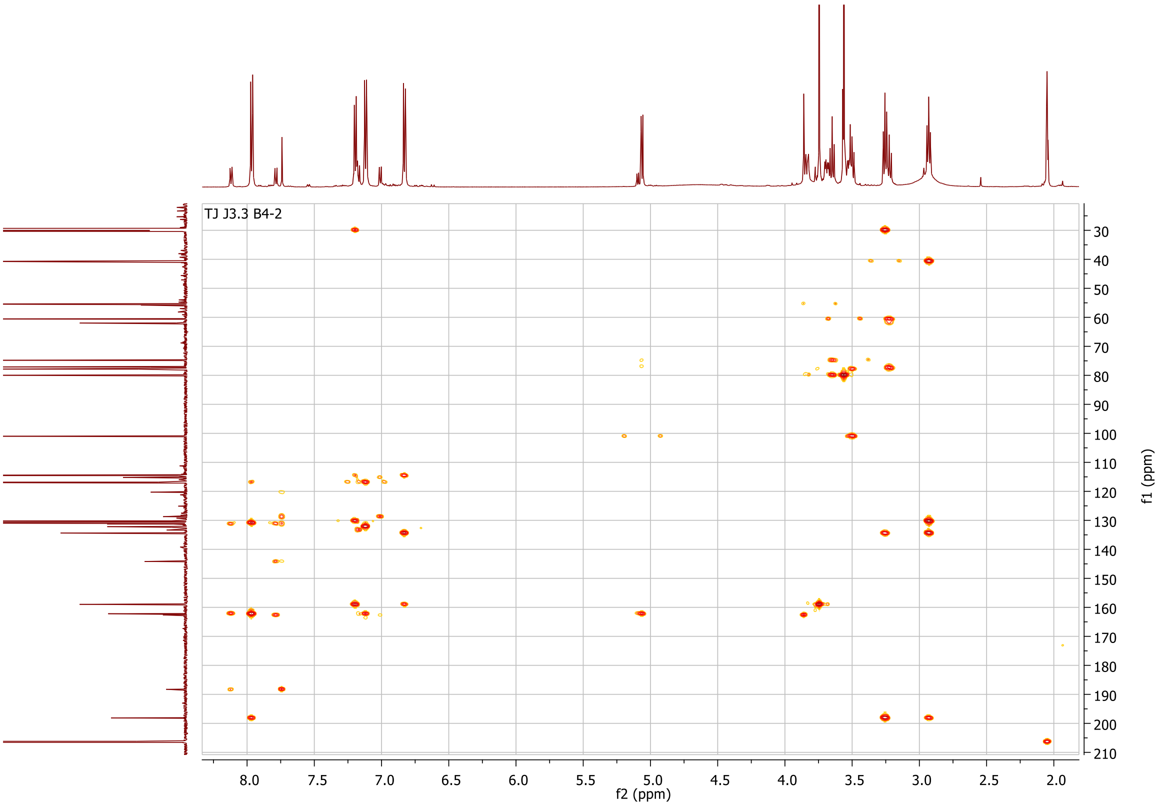

Figure S25. HMBC spectrum of 4′-*O*-β-D-(4″-*O*-methylglucopyranosyl)-4-methoxydihydrochalcone (**3B**) and 4′-*O*-β-D-(4″-*O*-methylglucopyranosyl)-4-methoxychalcone (**3C**) (Acetone-d_6, 600/151 MHz)

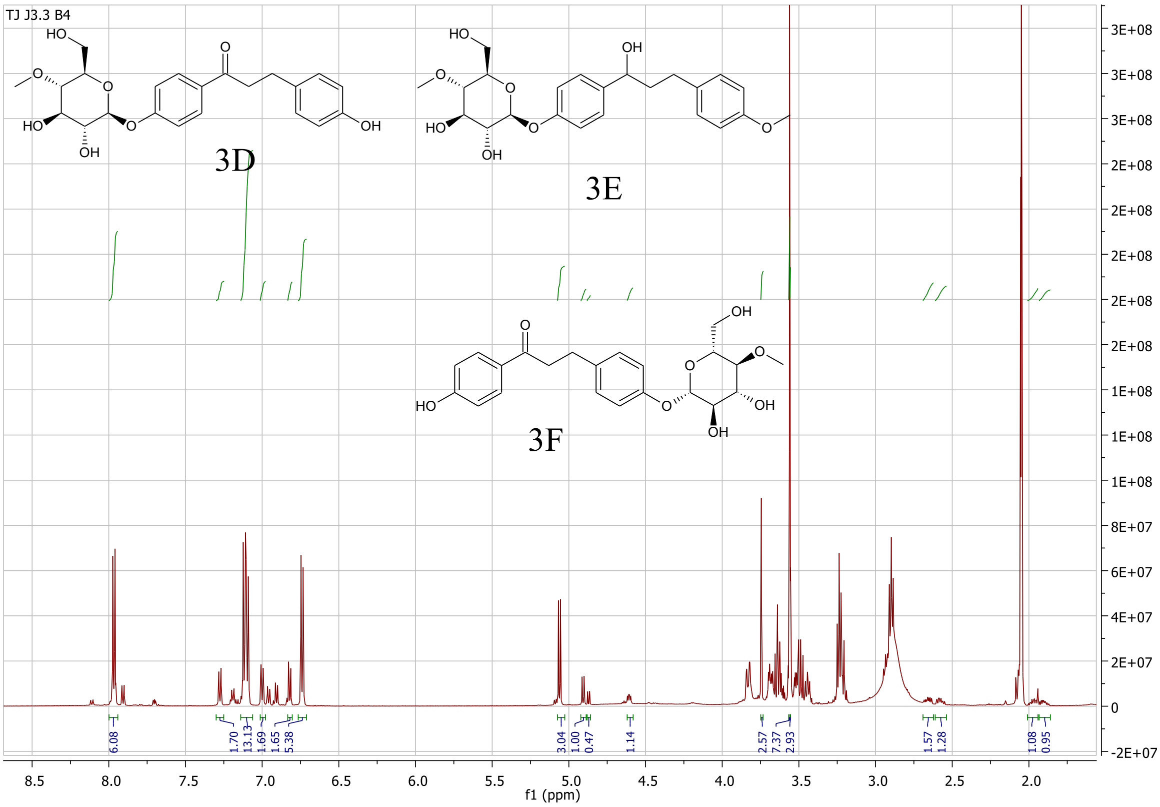

Figure S26. ^{1}H NMR spectra of 4′-*O*-β-D-(4″-*O*-methylglucopyranosyl)-4-hydroxydihydrochalcone (**3D**), 4′-*O*-β-D-(4″-*O*-methylglucopyranosyl)-4-methoxydihydrochalc-1-ol (**3E**) and 4-*O*-β-D-(4″-*O*-methylglucopyranosyl)-4′-hydroxydihydrochalcone (**3F**) (Acetone-d_6, 600 MHz)

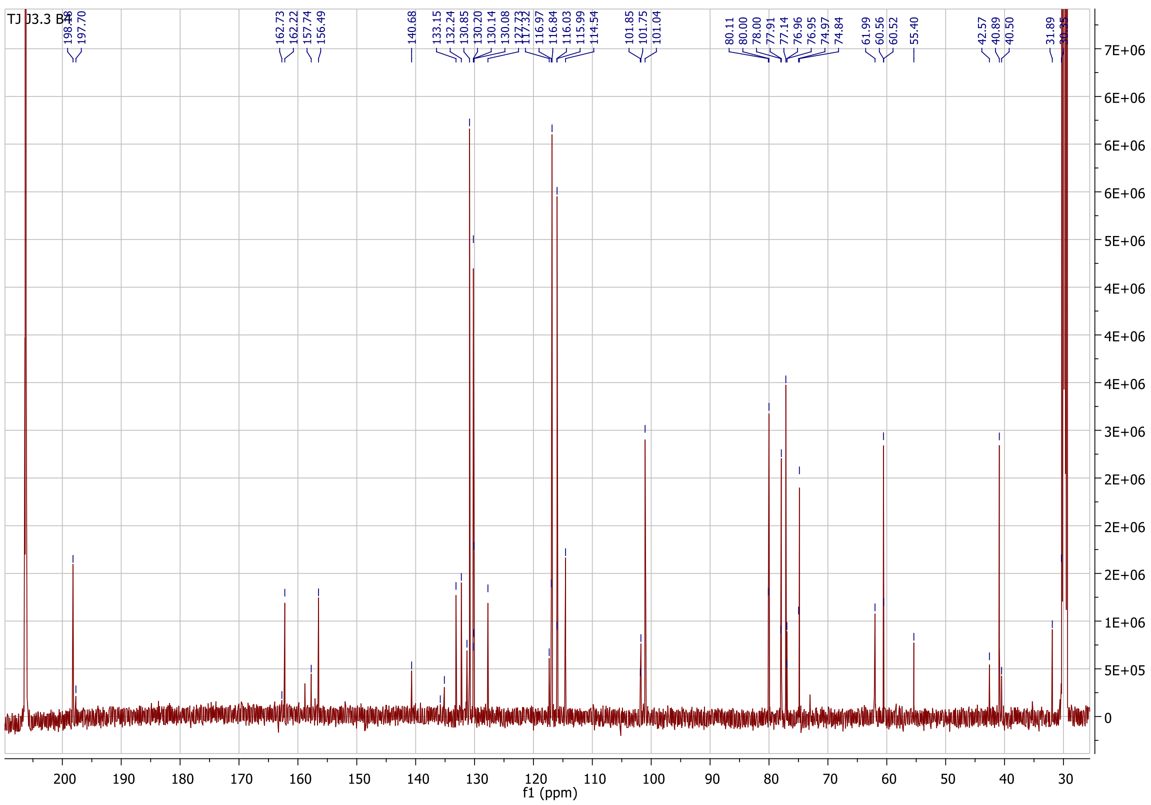

Figure S27. ^{13}C NMR spectra of 4′-*O*-β-D-(4″-*O*-methylglucopyranosyl)-4-hydroxydihydrochalcone (**3D**), 4′-*O*-β-D-(4″-*O*-methylglucopyranosyl)-4-methoxydihydrochalc-1-ol (**3E**) and 4-*O*-β-D-(4″-*O*-methylglucopyranosyl)-4′-hydroxydihydrochalcone (**3F**) (Acetone-d_6, 151 MHz)

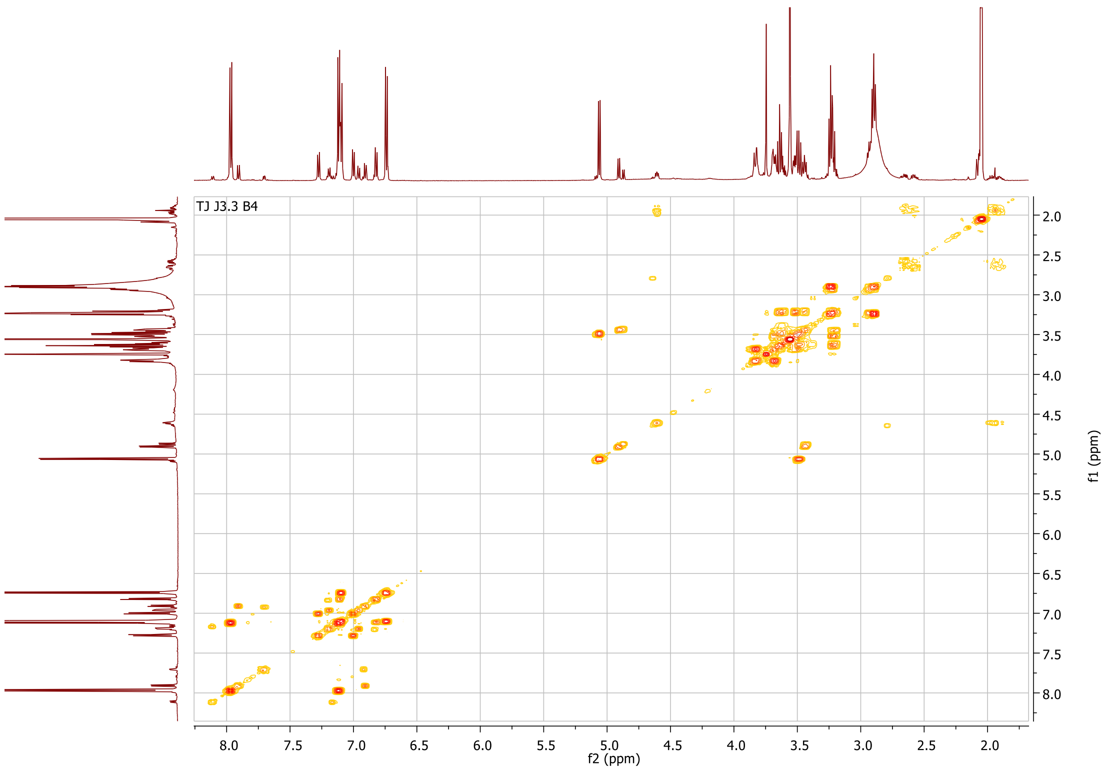

Figure S28. COSY spectrum of 4′-*O*-β-D-(4″-*O*-methylglucopyranosyl)-4-hydroxydihydrochalcone (**3D**), 4′-*O*-β-D-(4″-*O*-methylglucopyranosyl)-4-methoxydihydrochalc-1-ol (**3E**) and 4-*O*-β-D-(4″-*O*-methylglucopyranosyl)-4′-hydroxydihydrochalcone (**3F**) (Acetone-d_6, 600 MHz)

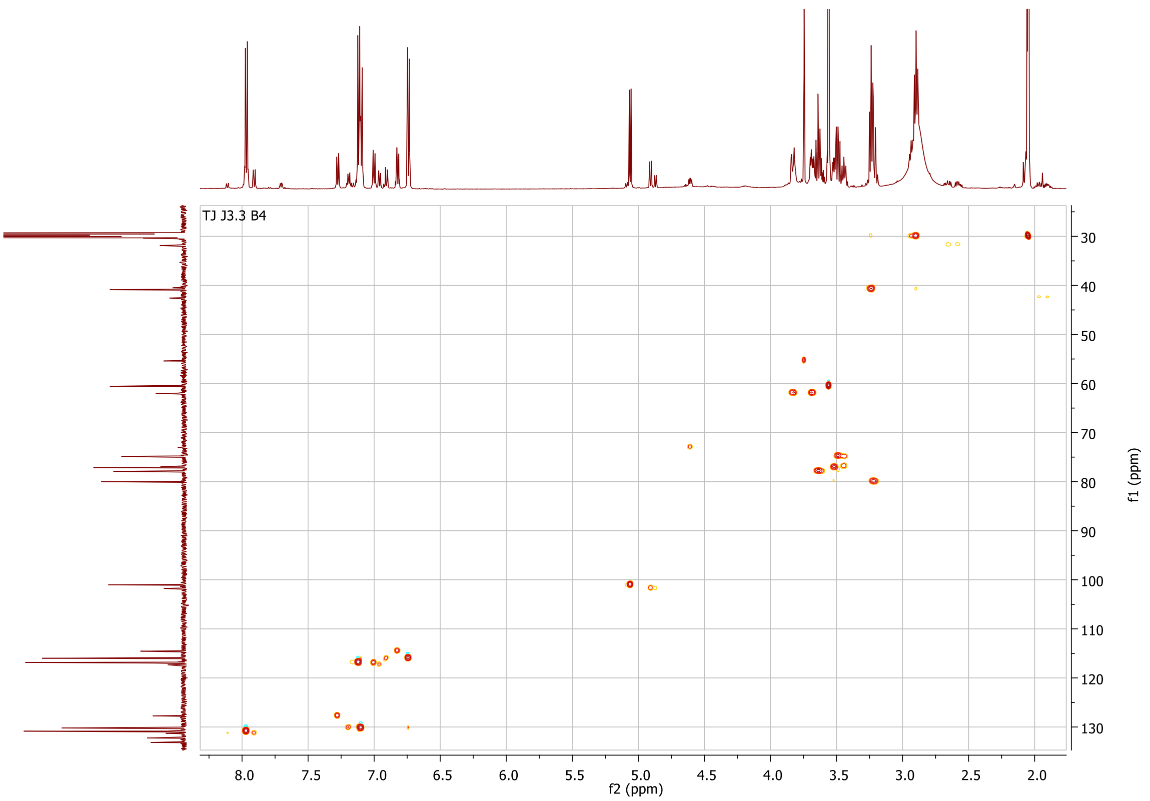

Figure S29. HSQC spectrum of 4′-*O*-β-D-(4″-*O*-methylglucopyranosyl)-4-hydroxydihydrochalcone (**3D**), 4′-*O*-β-D-(4″-*O*-methylglucopyranosyl)-4-methoxydihydrochalc-1-ol (**3E**) and 4-*O*-β-D-(4″-*O*-methylglucopyranosyl)-4′-hydroxydihydrochalcone (**3F**) (Acetone-d_6, 600/151 MHz)

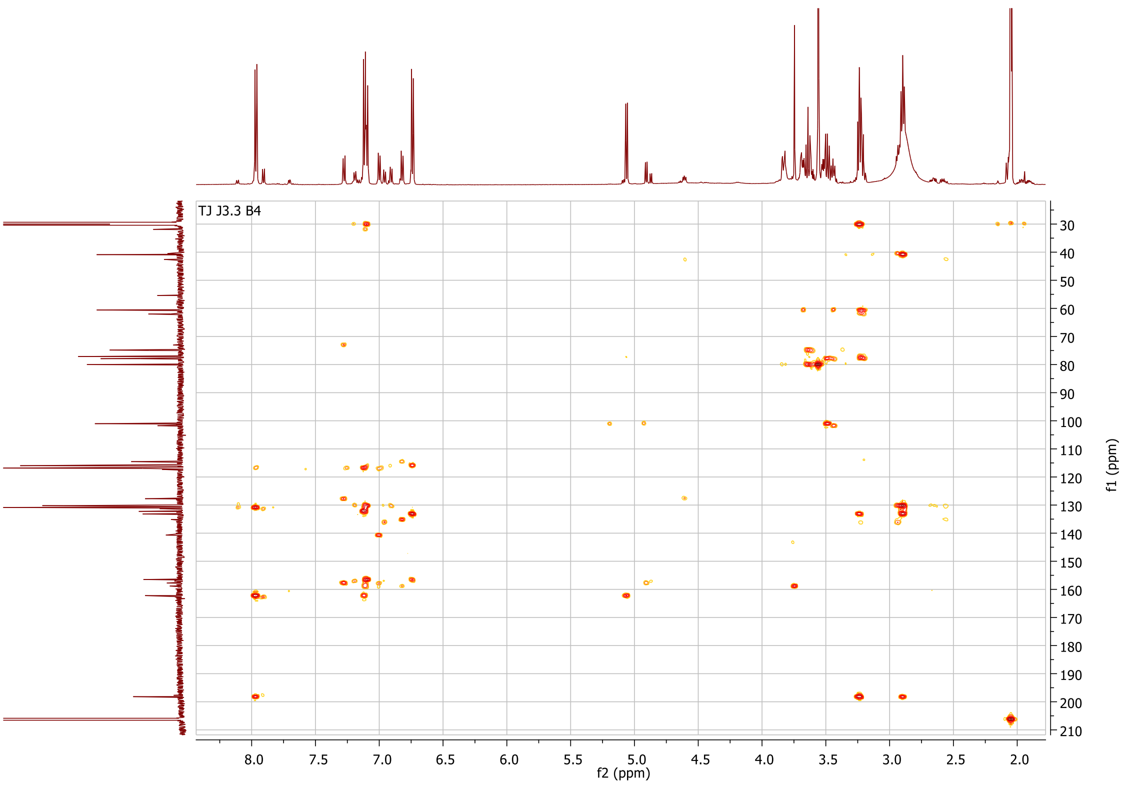

Figure S30. HMBC spectrum of 4′-*O*-β-D-(4″-*O*-methylglucopyranosyl)-4-hydroxydihydrochalcone (**3D**), 4′-*O*-β-D-(4″-*O*-methylglucopyranosyl)-4-methoxydihydrochalc-1-ol (**3E**) and 4-*O*-β-D-(4″-*O*-methylglucopyranosyl)-4′-hydroxydihydrochalcone (**3F**) (Acetone-d_6, 600/151 MHz)

Table S1. High-resolution ESI-QTOF-MS data for isolated or enriched biotransformation products.

Compound	Molecular formula	Ion	Ionization mode	Calculated *m/z*	Found *m/z*	Δ ppm
**1A**	C16H16O3	[M+H]+	ESI+	257.1172	257.1193	+8.1
**1B**	C23H28O8	[M+H]+	ESI+	433.1857	433.1870	+3.0
**2A**	C16H16O3	[M+H]+	ESI+	257.1172	257.1181	+3.4
**2B**	C23H28O8	[M+H]+	ESI+	433.1857	433.1863	+1.4
**2C**	C15H14O3	[M+H]+	ESI+	243.1016	243.1018	+0.9
**3A**	C16H16O3	[M+H]+	ESI+	257.1172	257.1176	+1.5
**3B**	C23H28O8	[M+H]+	ESI+	433.1857	433.1863	+1.4
**3C**	C23H26O8	[M+H]+	ESI+	431.1700	431.1706	+1.3
**3D/3F**	C22H26O8	[M+H]+	ESI+	419.1700	419.1702	+0.4
**3E**	C23H30O8	[M+Na]+	ESI+	457.1833	457.1838	+1.1

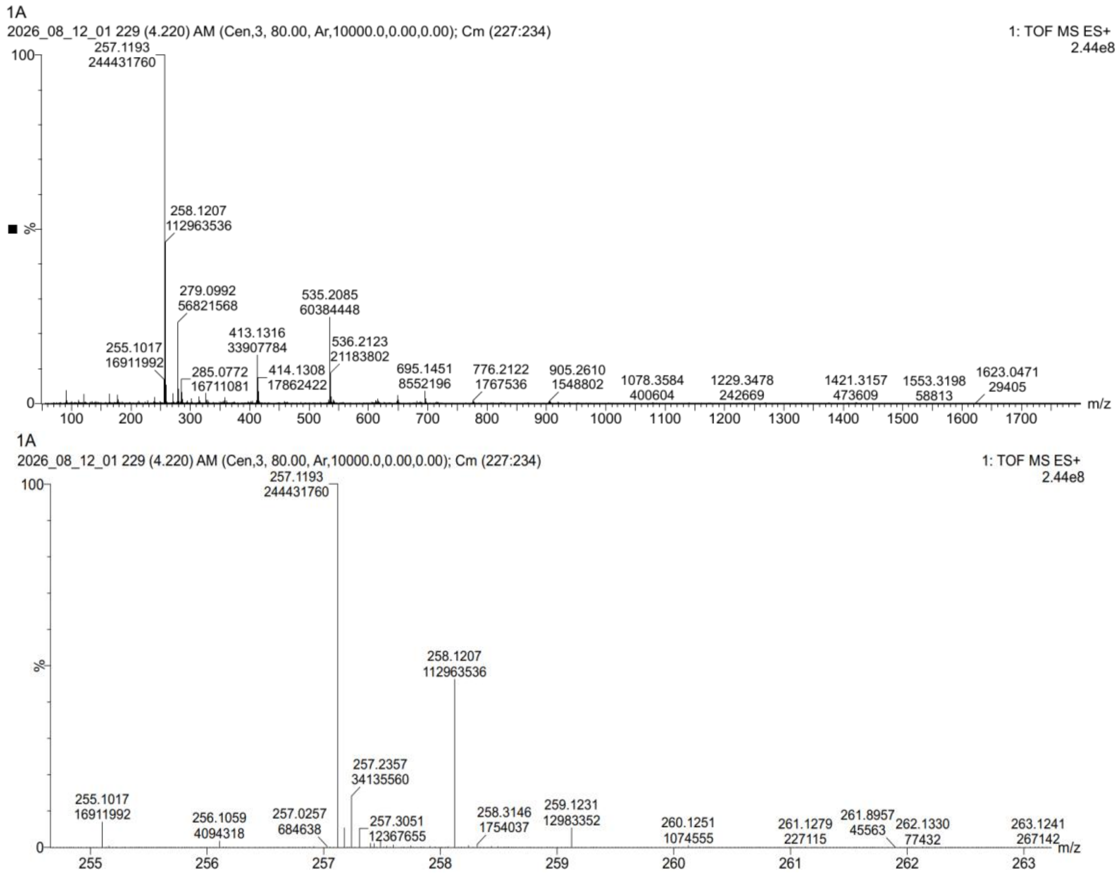

Figure S31. HR-ESI-QTOF-MS spectrum of 4′-hydroxy-2-methoxydihydrochalcone (**1A**) acquired in positive-ion mode (ESI+). $[M + H]^+$ at *m/z* 257.1193 (calcd. 257.1172; mass error = +8.1 ppm).

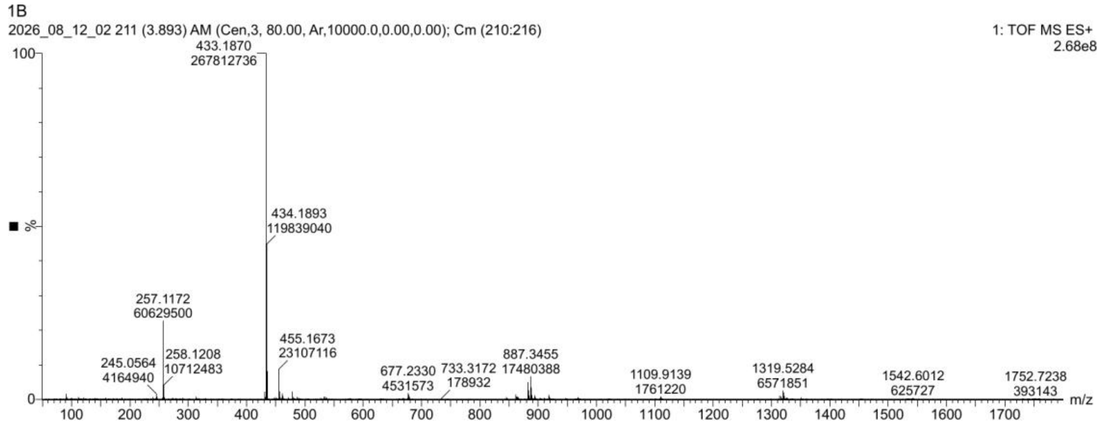

Figure S32. HR-ESI-QTOF-MS spectrum of 4′-*O*-β-D-(4″-*O*-methylglucopyranosyl)-2-methoxydihydrochalcone (**1B**) acquired in positive-ion mode (ESI+). $[M + H]^+$ at *m/z* 433.1870 (calcd. 433.1857; mass error = +3.0 ppm).

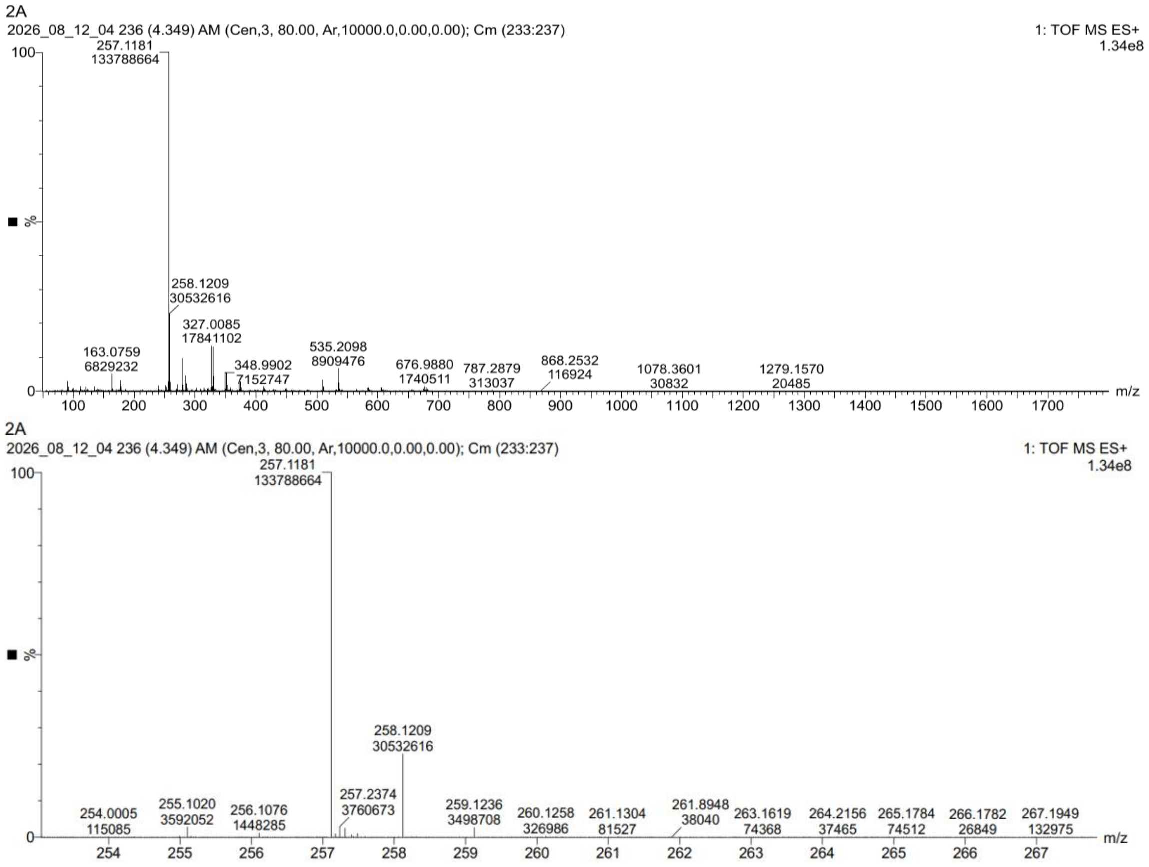

Figure S33. HR-ESI-QTOF-MS spectrum of 4′-hydroxy-3-methoxydihydrochalcone (**2A**) acquired in positive-ion mode (ESI+). $[M + H]^+$ at *m/z* 257.1181 (calcd. 257.1172; mass error = +3.4 ppm).

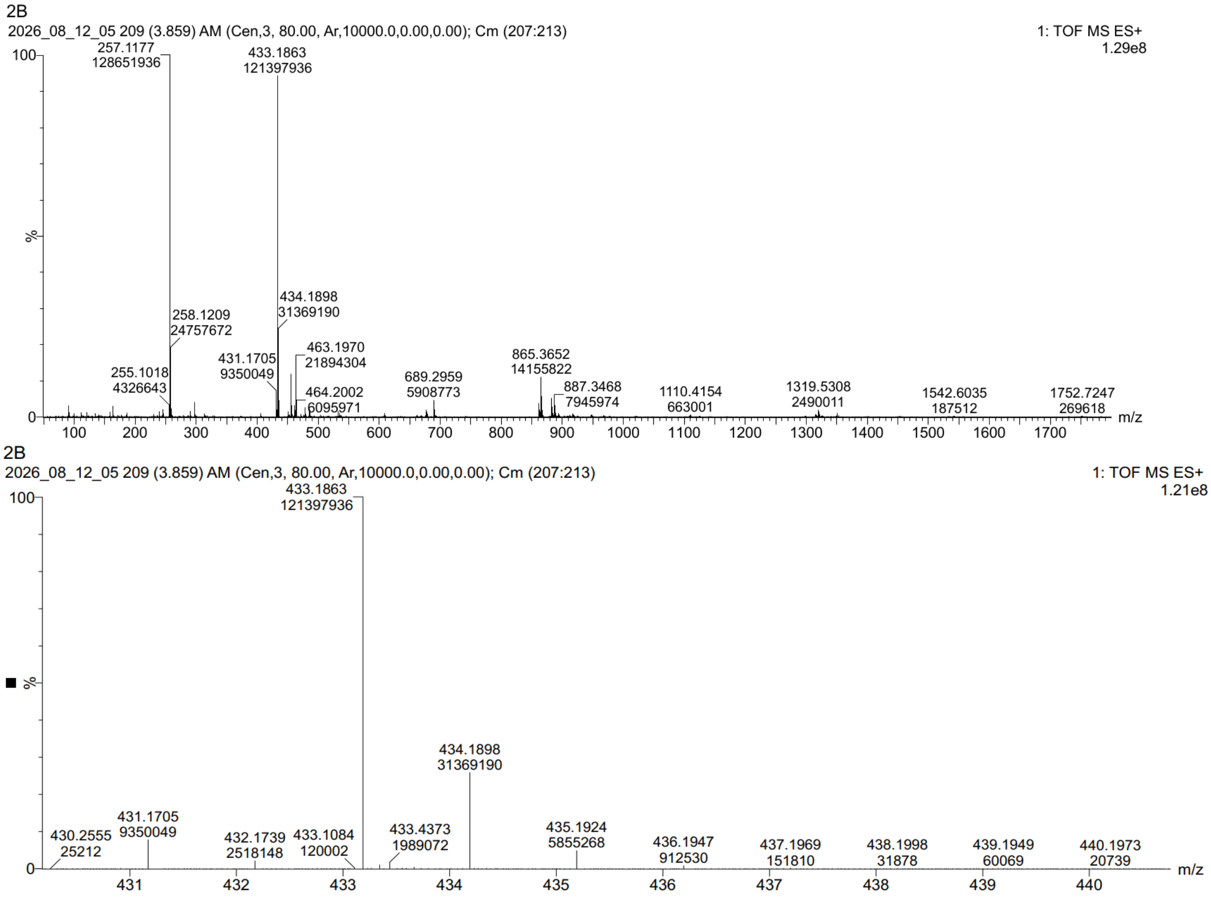

Figure S34. HR-ESI-QTOF-MS spectrum of 4′-*O*-β-D-(4″-*O*-methylglucopyranosyl)-3-methoxydihydrochalcone (**2B**) acquired in positive-ion mode (ESI+). $[M + H]^+$ at *m/z* 433.1863 (calcd. 433.1857; mass error = +1.4 ppm).

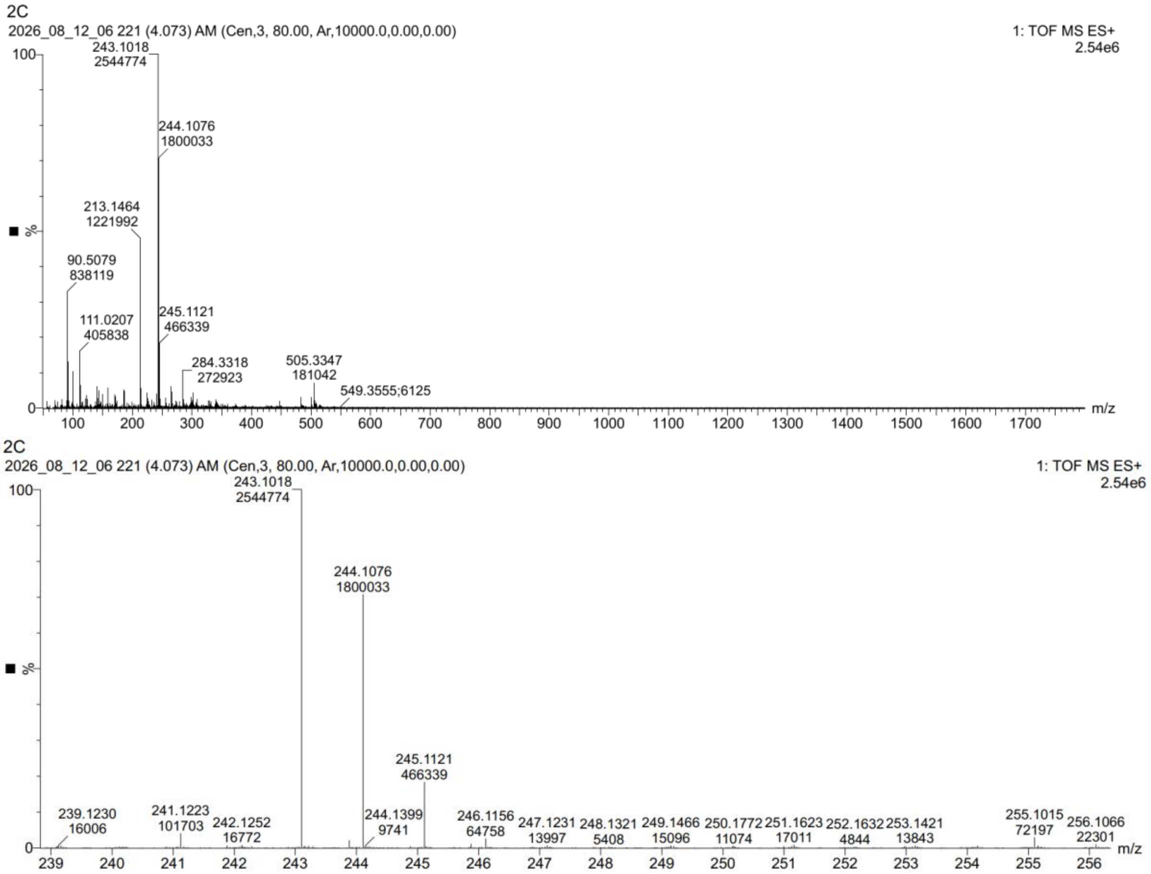

Figure S35. HR-ESI-QTOF-MS spectrum of 3,4′-dihydroxydihydrochalcone (**2C**) acquired in positive-ion mode (ESI+). $[M + H]^+$ at *m/z* 243.1018 (calcd. 243.1016; mass error = +0.9 ppm).

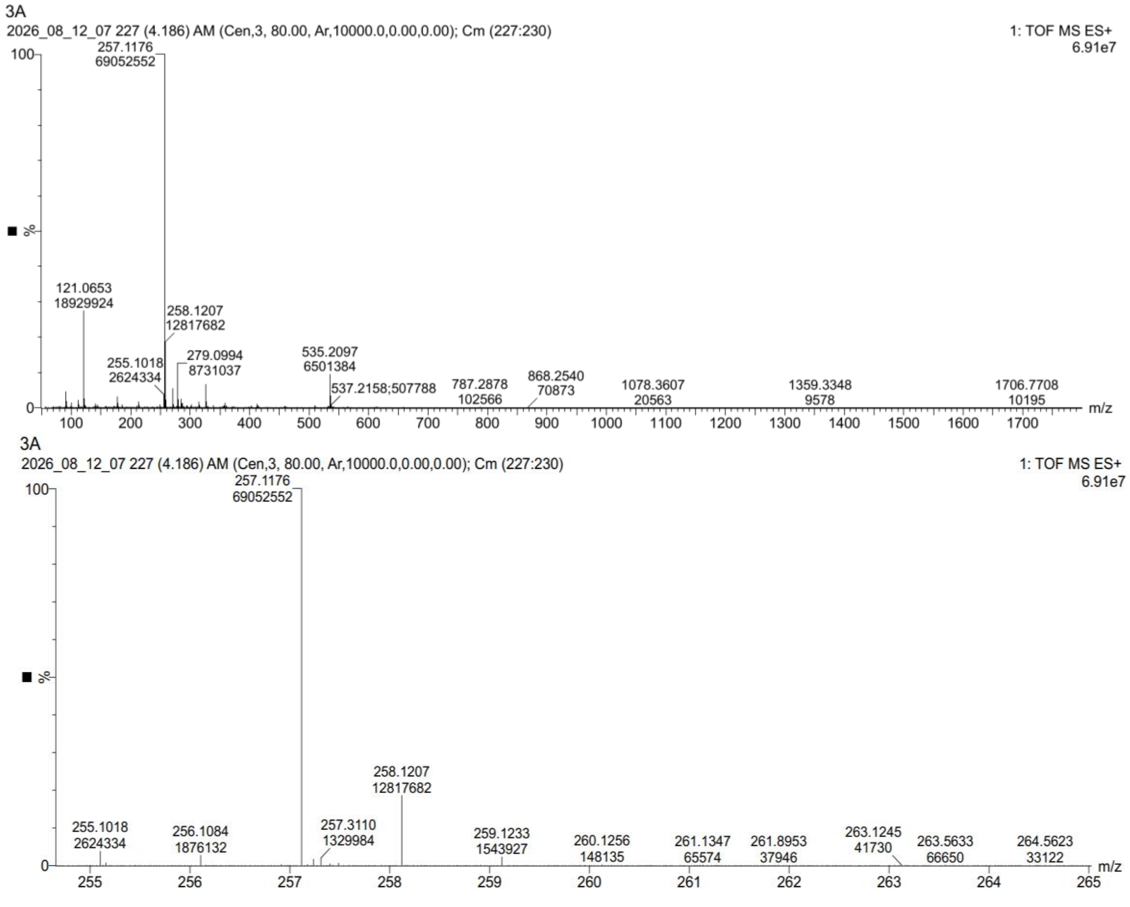

Figure S36. HR-ESI-QTOF-MS spectrum of 4′-hydroxy-4-methoxydihydrochalcone (**3A**) acquired in positive-ion mode (ESI+). $[M + H]^+$ at *m/z* 257.1176 (calcd. 257.1172; mass error = +1.5 ppm).

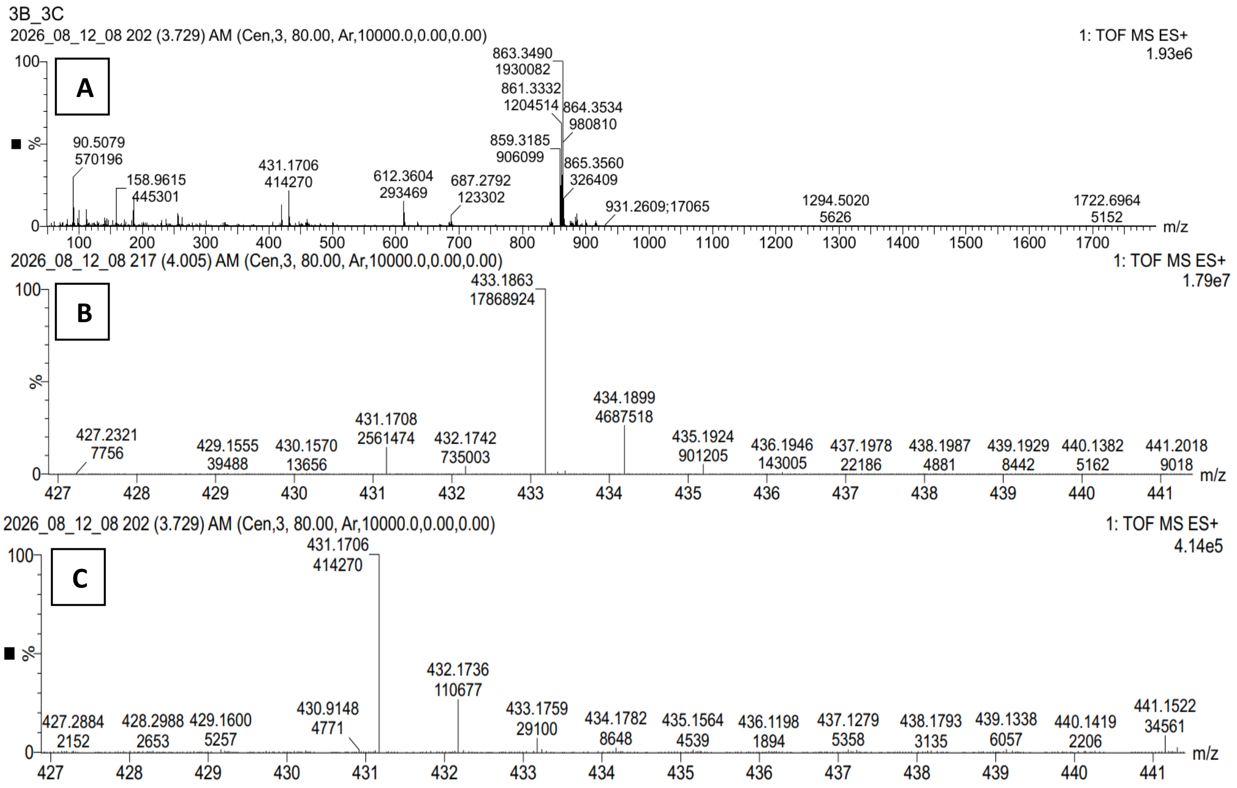

Figure S37. HR-ESI-QTOF-MS analysis of the enriched fraction containing compounds **3B** and **3C**, acquired in positive-ion mode (ESI+). (A) Full-scan mass spectrum of the enriched fraction. (B) Enlarged region showing the protonated molecular ion of 3B, $[M + H]^+$ at *m/z* 433.1863 (calcd. 433.1857; mass error = +1.4 ppm). (C) Enlarged region showing the protonated molecular ion of **3C**, $[M + H]^+$ at *m/z* 431.1706 (calcd. 431.1700; mass error = +1.3 ppm).

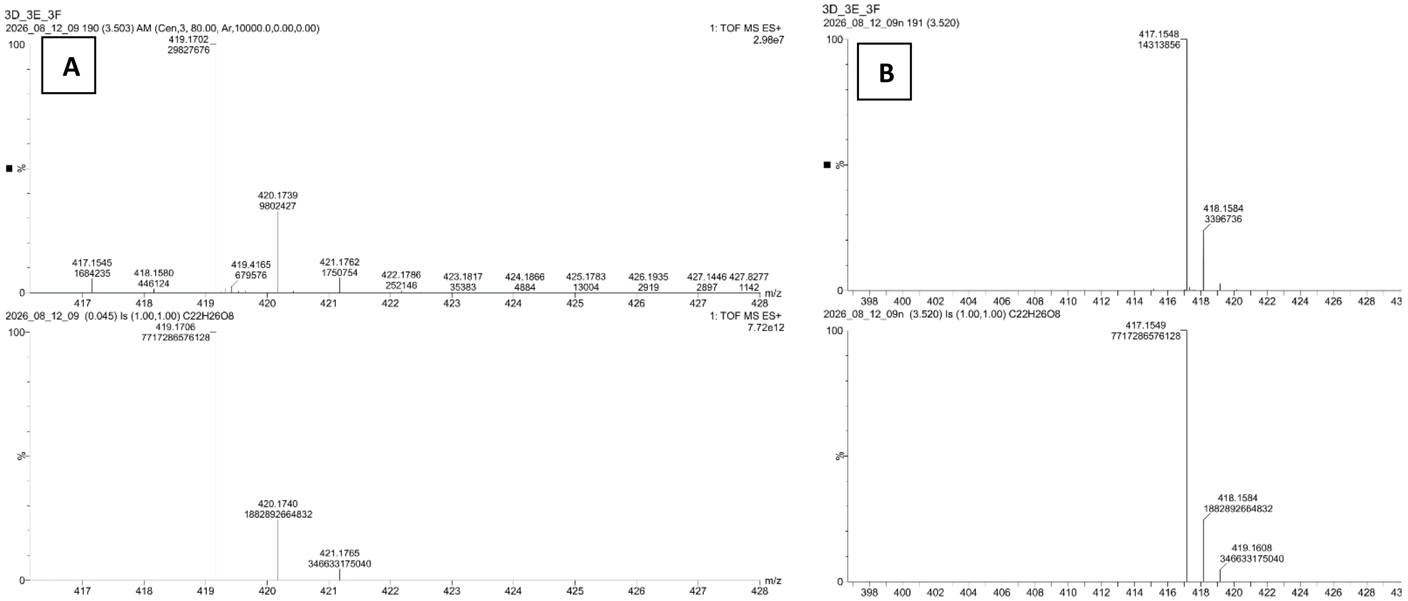

Figure S38. HR-ESI-QTOF-MS spectra supporting the elemental composition assigned to the isomeric compounds **3D** and **3F** in the enriched fraction. (A) Positive-ion mode (ESI+) spectrum showing [M + H]+ at *m/z* 419.1702; the calculated isotope distribution for C22H26O8 is shown in the lower panel of the analytical report. (B) Negative-ion mode (ESI−) spectrum showing [M − H]− at *m/z* 417.1548; the corresponding calculated isotope distribution is shown below. Because **3D** and **3F** are isomers with the same elemental composition, HRMS does not distinguish between the two proposed structures.

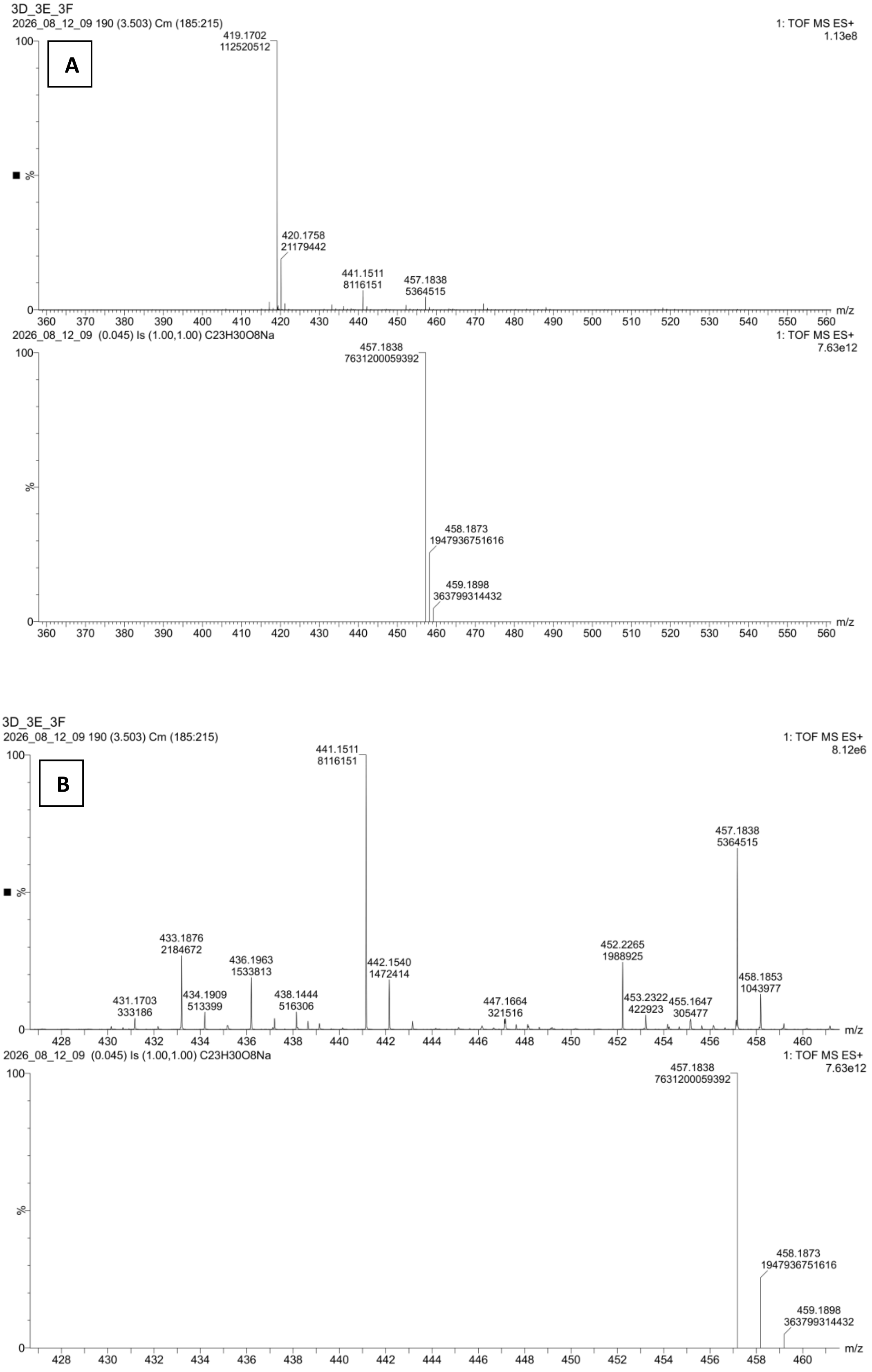

Figure S39. HR-ESI-QTOF-MS analysis supporting the elemental composition assigned to compound **3E** in the enriched fraction. (A) Positive-ion mode (ESI+) spectrum showing the sodium adduct [M + Na]+ at *m/z* 457.1838; the calculated isotope distribution for C23H30O8Na is shown in the lower panel. (B) Enlarged ESI+ region around the sodium adduct, showing the signal at *m/z* 457.1838 and its isotope pattern. The sodium-adduct measurement provides additional support for the proposed elemental composition of **3E**; however, **3E** was characterized as a component of an enriched multicomponent fraction rather than as an individually purified metabolite.

Table S2. Time-resolved UPLC-DAD profiles of the biotransformation of compounds **1–3** by entomopathogenic filamentous fungi

Substrate	Strain	Day 1	Day 3	Day 7	Day 10
1	*B. caledonica* KCh J3.3	**1B** dominant, traces of **1** + **1A**	**1B** only	**1B** only	**1B** only
	I. tenuipes MU35	**1A** dominant, traces of **1** + **1B**	**1B** dominant; traces of **1/1A**	**1B** only	**1B** only
	B. bassiana KCh J1.5	**1B**; traces of **1**; putative **1D**	**1B** + **1D** + polar u.c.	Similar to day 3	Similar to day 3
	B. bassiana KCh J1	**1** + **1A**	**1A** dominant + traces of **1C**	**1A** dominant + traces of **1C**	**1A** dominant + traces of **1C**
	M. robertsii MU4	**1** + **1B**	**1B** dominant; traces of **1C** + polar u.c.	**1B** + increase of polar u.c.	Similar to day 7
	I. fumosorosea KCh J2	**1** dominant; traces of **1A** + **1B**	**1** dominant; **1B** + **1D**	**1** + polar u.c.	**1** + increase of polar u.c.
	B. bassiana KCh BBT	**1B** dominant; traces of **1**	**1B** only	**1B** only	**1B** only
	I. farinosa KCh KW1.1	**1** dominant; **1B**	**1B** + **1C** + **1D** + polar u.c.	**1B** dominant + polar u.c.	**1B** dominant + polar u.c.
2	*B. caledonica* KCh J3.3	**2A** dominant; traces **2** + **2B**	**2B** only	**2B** only	**2B** only
	I. tenuipes MU35	**2A** dominant; traces of **2**	**2A** dominant; traces of **2B** + **2C**	**2B** + **2C** + Polar u.c.	**2B** + **2C** + Polar u.c.
	B. bassiana KCh J1.5	**2A** dominant; **2B** + **2C**	**2B + Polar u.c.**	Similar to day 3	Similar to day 3
	B. bassiana KCh J1	**2A** only	**2A + traces of 2C**	**2A:2C = 78:22**	**2A:2C = 59:41**
	M. robertsii MU4	**2A** dominant; **2B** + **2C**	**2B** dominant; **2C** + polar u.c.	**2B** + polar u.c.	**2B** + polar u.c.
	I. fumosorosea KCh J2	**2A** dominant; traces of **2B** + **2C**	**2B** dominant	**2B** dominant + increase of polar u.c.	**2B** dominant + increase of polar u.c.
	B. bassiana KCh BBT	**2A** dominant; traces of **2B**	**2B** dominant	**2B** dominant	**2B** dominant
	I. farinosa KCh KW1.1	**2A** dominant; traces of **2** + **2C**	**2A** + **2C**; traces of **2B**	**2B** dominant + polar u.c.	**2B** dominant + polar u.c.
3	*B. caledonica* KCh J3.3	**3A** dominant; traces of **3** + **3B**	**3B** dominant	Similar to day 3	Similar to day 3
	I. tenuipes MU35	**3A** dominant; traces of **3**	**3A** + traces of **3B** and **3D**	increase of polar u.c.	further increase
	B. bassiana KCh J1.5	**3B** + **3D** = 66:34	**3B** + **3D** = 59:41	**3B+3D** = 59:41	**3B+3D** = 59:41
	B. bassiana KCh J1	**3A** only	**3A:** demethylated dihydrochalcone = **96:4**	**3A:** demethylated dihydrochalcone = 96:4	**3A:** demethylated dihydrochalcone **= 71:29**
	M. robertsii MU4	**3B** dominant; traces of **3A** + polar u.c.	**3B** dominant + polar u.c. + traces of **3A**	**3B:**polar u.c. **= 78:22**	**3B:** polar u.c. **= 78:22**
	I. fumosorosea KCh J2	**3A** dominant; traces of **3** + **3B**	**3B** dominant	**3B** dominant + polar u.c.	**3B** dominant + increase of polar u.c.
	B. bassiana KCh BBT	**3A** dominant, traces of **3B**	**3B** dominant + polar u.c.	Similar to day 3	Similar to day 3
	I. farinosa KCh KW1.1	**3A** dominant; **3**	**3A** dominant + **3B** + polar u.c.	**3B** dominant; traces of putative **3C**	**3B** dominant; traces of putative **3C**

The table summarizes qualitative and semi-quantitative UPLC-DAD observations. Numerical values are given only for pairs of closely related metabolites displaying comparable DAD spectral profiles and represent uncorrected relative peak-area ratios at the selected detection wavelength. Peak areas of parent chalcones and the corresponding dihydrochalcones were not directly compared because reduction of the α,β-unsaturated bond substantially changes the chromophore and wavelength-dependent detector response. Accordingly, the data should not be interpreted as molar product distributions, conversion values, absolute concentrations, or biotransformation yields. u.c. – unidentified compound(s)

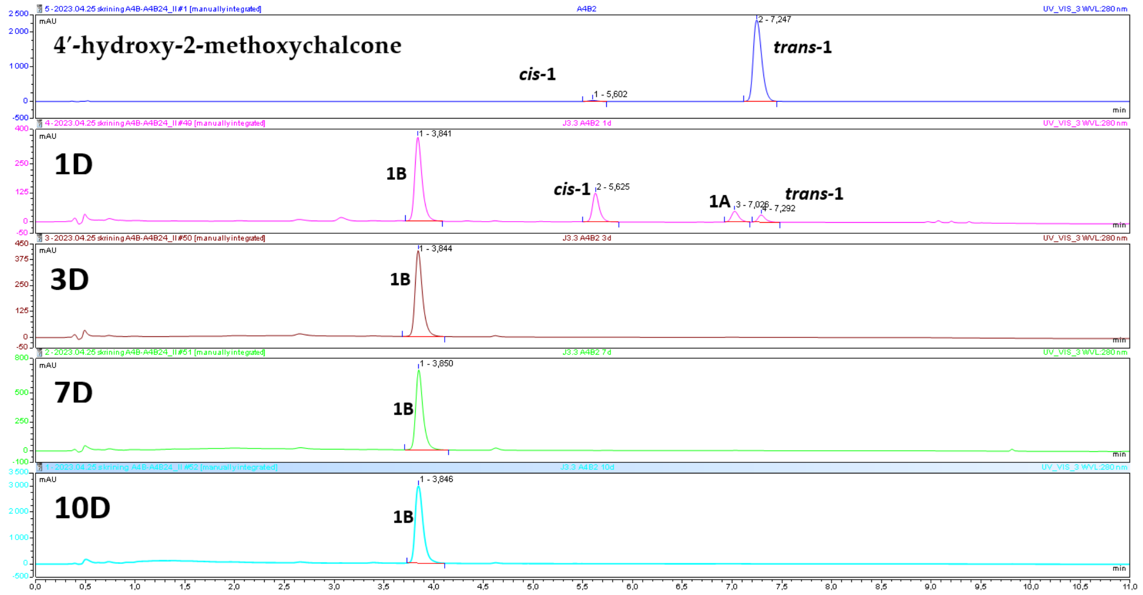

Figure S40. Time-course UPLC-DAD chromatograms of the biotransformation of 4′-hydroxy-2-methoxychalcone (**1**) recorded at 280 nm after 1, 3, 7, and 10 days.

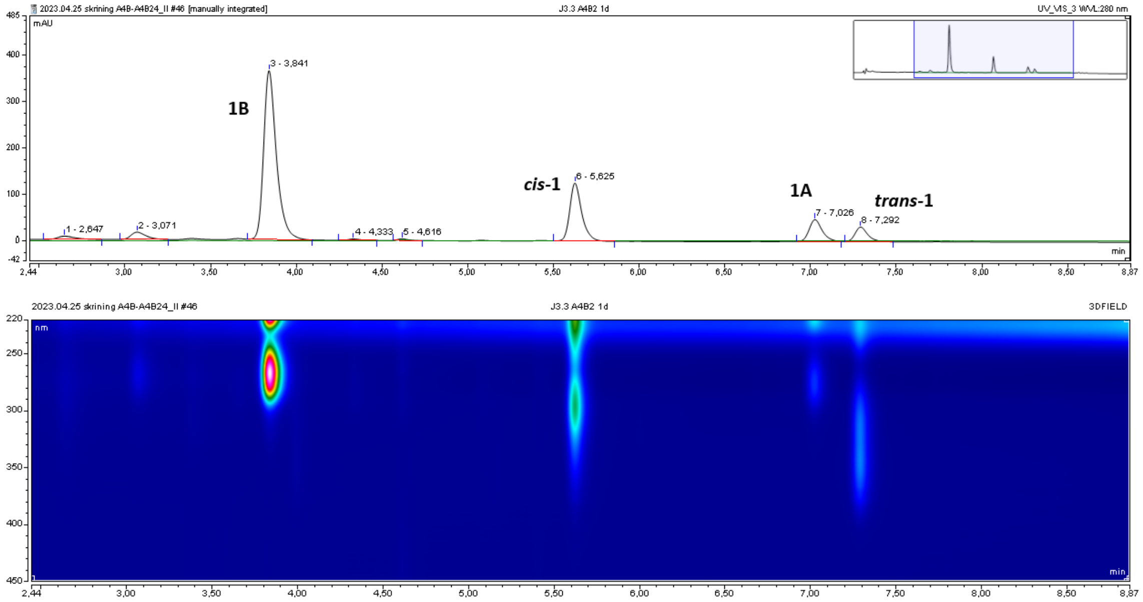

Figure S41. 3D chromatogram of biotransformation of 4′-hydroxy-2-methoxychalcone (**1**) in the 1st day. Horizontal axis: time; vertical axis: λ [nm].

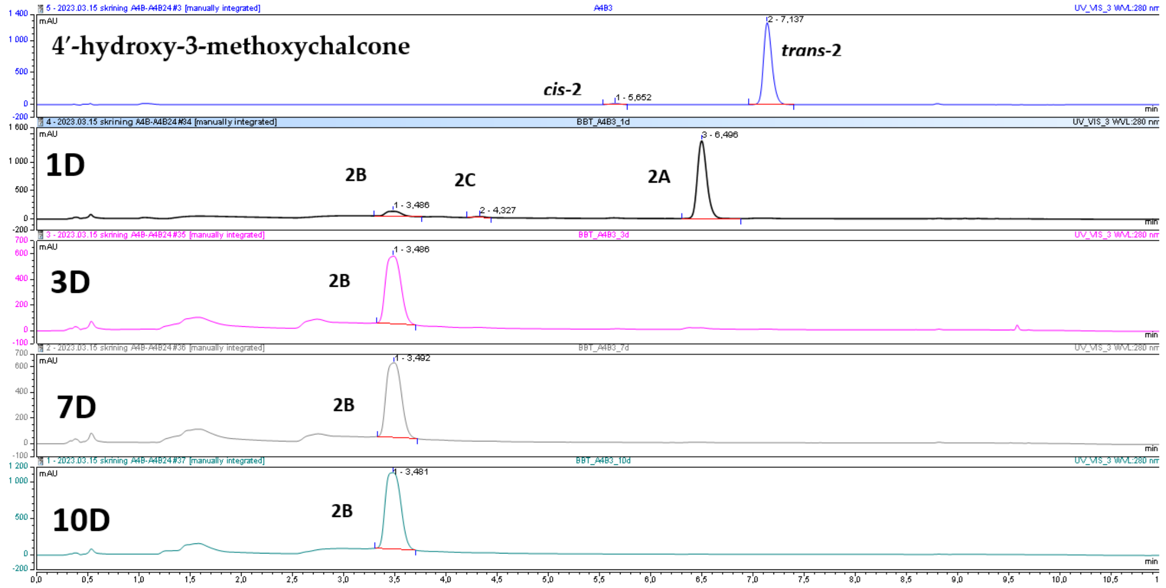

Figure S42. Time-course UPLC-DAD chromatograms of the biotransformation of 4′-hydroxy-3-methoxychalcone (**2**) recorded at 280 nm after 1, 3, 7, and 10 days.

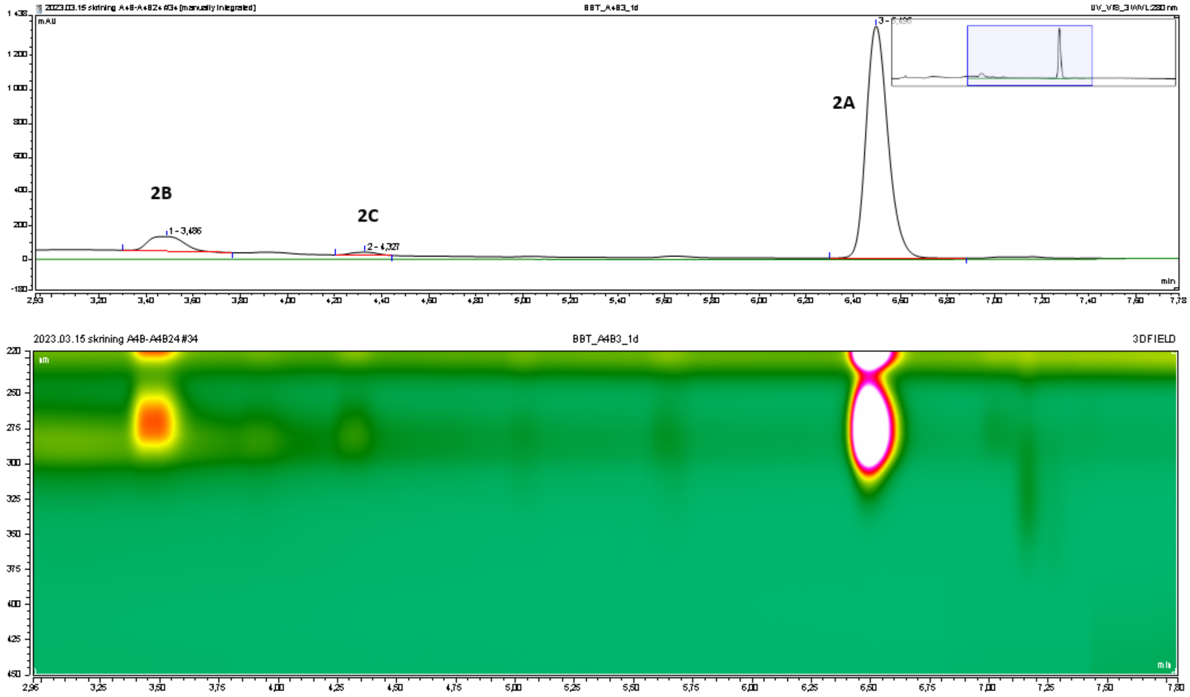

Figure S43. 3D chromatogram of biotransformation of 4′-hydroxy-3-methoxychalcone (**2**) in the 1st day. Horizontal axis: time; vertical axis: λ [nm].

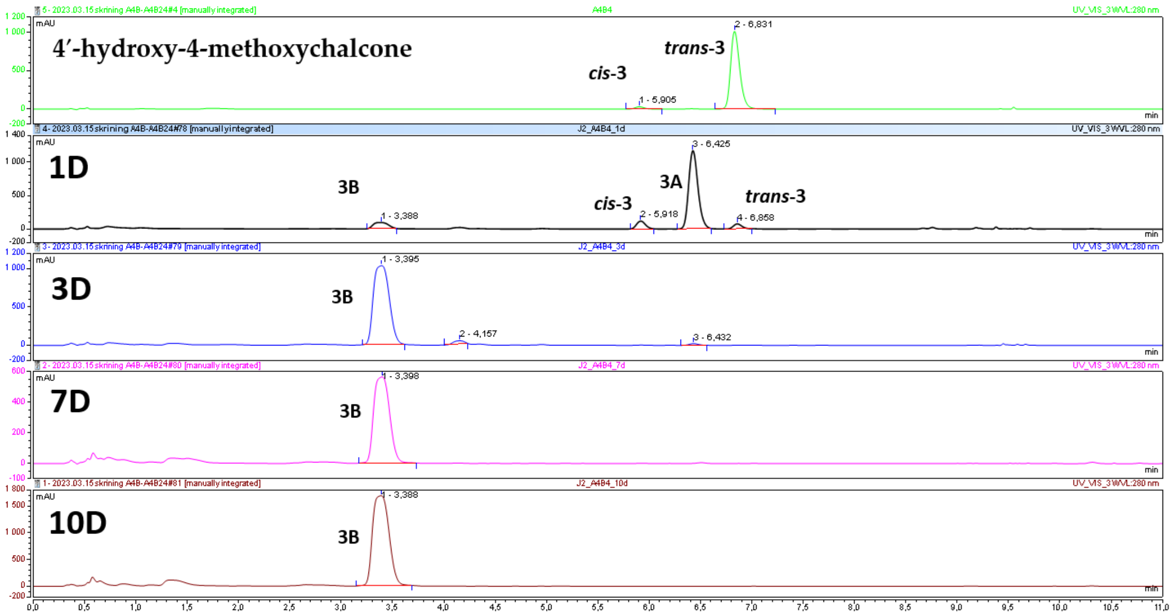

Figure S44. Time-course UPLC-DAD chromatograms of the biotransformation of 4′-hydroxy-4-methoxychalcone (**3**) recorded at 280 nm after 1, 3, 7, and 10 days.

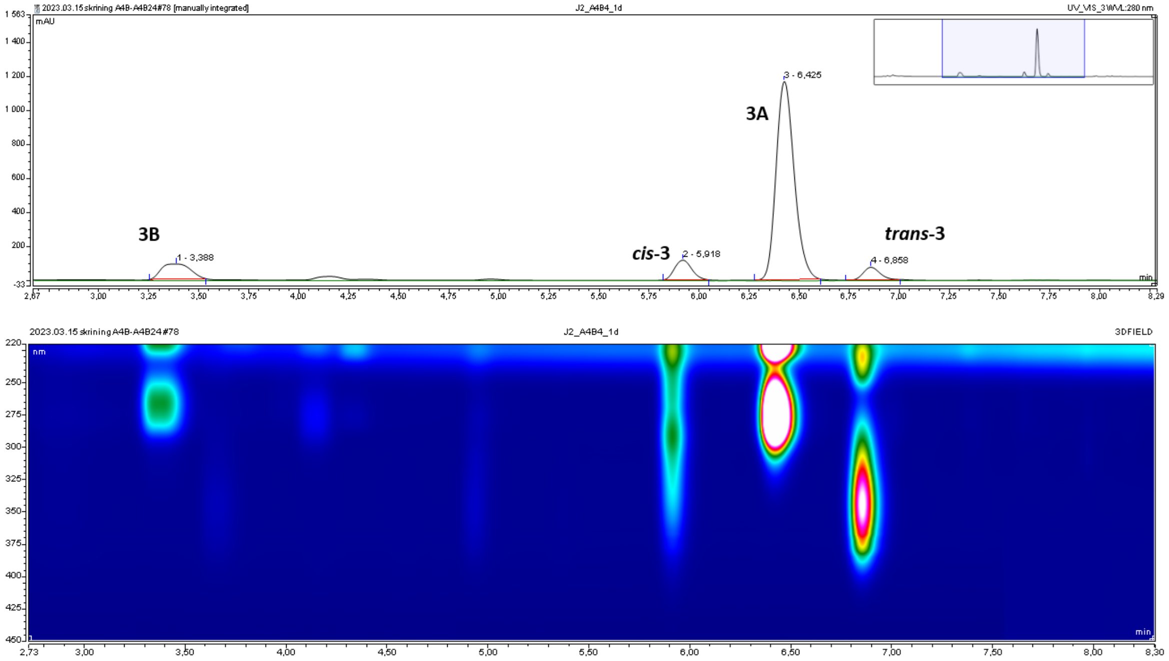

Figure S45. 3D chromatogram of biotransformation of 4′-hydroxy-4-methoxychalcone (**3**) in the 1st day. Horizontal axis: time; vertical axis: λ [nm].
